# Progression of irradiated mesenchymal stromal cells from early to late senescence: Changes in SASP composition and anti‐tumour properties

**DOI:** 10.1111/cpr.13401

**Published:** 2023-03-22

**Authors:** Nicola Alessio, Mustafa Burak Acar, Tiziana Squillaro, Domenico Aprile, Şerife Ayaz‐Güner, Giovanni Di Bernardo, Gianfranco Peluso, Servet Özcan, Umberto Galderisi

**Affiliations:** ^1^ Department of Experimental Medicine Luigi Vanvitelli Campania University Naples Italy; ^2^ Genome and Stem Cell Center (GENKÖK) Erciyes University Kayseri Turkey; ^3^ Department of Molecular Biology and Genetics, Faculty of Life and Natural Science Abdullah Gül University Kayseri Turkey; ^4^ Department of Molecular Biology and Genetics Izmir Institute of Technology Izmir Turkey; ^5^ The Interuniversity Consortium “Istituto Nazionale Biostrutture e Biosistemi” (INBB – Biostructures and Biosystems National Institute) Rome Italy; ^6^ UniCamillus Rome Italy; ^7^ Department of Biology, Faculty of Science Erciyes University Kayseri Turkey; ^8^ Sbarro Institute for Cancer Research and Molecular Medicine, Center for Biotechnology Temple University Philadelphia Pennsylvania USA

## Abstract

Genotoxic injuries converge on senescence‐executive program that promotes production of a senescence‐specific secretome (SASP). The study of SASP is particularly intriguing, since through it a senescence process, triggered in a few cells, can spread to many other cells and produce either beneficial or negative consequences for health. We analysed the SASP of quiescent mesenchymal stromal cells (MSCs) following stress induced premature senescence (SIPS) by ionizing radiation exposure. We performed a proteome analysis of SASP content obtained from early and late senescent cells. The bioinformatics studies evidenced that early and late SASPs, besides some common ontologies and signalling pathways, contain specific factors. In spite of these differences, we evidenced that SASPs can block in vitro proliferation of cancer cells and promote senescence/apoptosis. It is possible to imagine that SASP always contains core components that have an anti‐tumour activity, the progression from early to late senescence enriches the SASP of factors that may promote SASP tumorigenic activity only by interacting and instructing cells of the immune system. Our results on Caco‐2 cancer cells incubated with late SASP in presence of peripheral white blood cells strongly support this hypothesis. We evidenced that quiescent MSCs following SIPS produced SASP that, while progressively changed its composition, preserved the capacity to block cancer growth by inducing senescence and/or apoptosis only in an autonomous manner.

## Graphical abstract

SASP content may be classified into two main components, one acts in an autonomous manner and the other through crosstalk with immune cells. The progression from early to late SASP enriches the secretome in the factors that interact with immune cells (pro‐inflammatory factors). The autonomous SASP component may block the growth of cancer cells and may induce senescence and/or apoptosis. The pro‐inflammatory factors may overcome the activity of autonomous components only in the presence of immune cells (PBMCs) by instructing them and promoting cancer cell survival and growth.

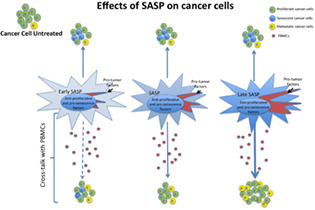

## 1 BACKGROUND

In multicellular organisms, many cell types, including stem cells, are in quiescent state, which is a reversible exit from the cell cycle with an absence of proliferation. This temporary cell cycle exit is a fundamental event for regulation of tissue development and homeostasis, indeed, an impaired control of cell cycle with aberrant proliferation or permanent cell cycle arrest may promote cancer and aging.^1^, ^2^ Quiescence delays cell senescence by reducing damage to macromolecules deriving from DNA replication, metabolic activity, gene transcription and translation. Quiescent cells are, however, highly susceptible to extrinsic genotoxic damage, since they have reduced expression of repair factors and may live for a long time, thus accumulating multiple injuries.^2^ Following an extrinsic genotoxic stimulus, cells, including those in quiescence, may either properly repair the DNA damage, or alternately unrepaired/misrepaired DNA may trigger apoptosis or senescence.

Senescent cells permanently exit the cell cycle, losing their primary functions and acquiring new ones. Senescent cells can contribute to organismal aging but also play a role in tissue development and wound healing. Senescence may counteract cancer but can also promote tumorigenesis.^3^, ^4^ The pleiotropic activities of senescent cells are due to an intrinsic dynamic state of senescence, which does not have a static endpoint. Following noxious stimuli, a cell entering senescence will have genetic and epigenetic changes that initially lead to stable cell‐cycle arrest, sustained by P53 and retinoblastoma pathways (early senescence). Senescent cells change their metabolism and lysosomal activity and secrete a plethora of factors, collectively indicated as senescence‐associated secretory phenotype (SASP). Senescent cells accomplish their divergent tasks by SASP autocrine/paracrine activity. In our body, senescent cells are cleared by the immune system, but some of them may escape this patrolling phenomenon and may remain for a long time in tissues. As time goes by, senescent cells enter into a late stage (late/deep senescence) and modify the SASP composition, which will be enriched in pro‐inflammatory factors.^4^, ^5^ Generally speaking, the beneficial effects of senescence (anti‐cancer properties, wound healing, contribution to tissue development) are due to SASP produced by senescent cells not into their final stage, while negative senescence outcomes (cancer promotion, aging) are mainly due to pro‐inflammatory SASP activity. It is then important to evaluate as SASP changes its composition over time and we need to see how these modifications affect its biological activities.

Mesenchymal stromal cells (MSCs) are a heterogeneous population containing stromal cells, fibroblasts, progenitor cells and stem cells that can differentiate into mesodermal derivatives. MSCs are present in the stroma of many tissues and through secretion of cytokines, growth and survival factors may contribute to tissue healing and homeostasis.^6^

The tumour microenvironment mimics an injured tissue and hence tumour growth often appears associated with several types of stromal cells in a manner that overlaps wound‐healing and tissue‐repair processes. In tumours, MSCs and other stromal cells are recruited to establish a supportive stroma.^7^ Any senescence phenomenon affecting MSCs may have a role in tumour growth and survival.

In this context, we wanted to evaluate the effects of SASP released by early and late senescent MSCs on cancer cell biology.

Several findings on senescence have been performed on proliferating cells that exit cell cycle and become senescent cells following a stressful stimulus. Nevertheless, in our body many cells are in a quiescent state and hence it is important to evaluate senescence of cells that were already resting when exposed to genotoxic stimuli.

In this context, in quiescent MSCs we induced senescence by x‐ray irradiation and evaluated changes in SASP composition through progression from early to late senescence. We also evaluated the SASP anti‐tumour properties on several colon cancer cell lines, chosen for differences in their genetic and epigenetic background, which affect their biological properties.

## 2 MATERIALS AND METHODS

### 2.1 Cultivation of mesenchymal stromal cell (MSCs) and quiescence induction

Human bone marrow MSCs (Catalog PT‐2501) were obtained (Lonza Bioscience, Rome, Italy) and cultivated according to the manufacturer's instructions. In detail, the cells after thawing were cultivated in proliferating medium: alpha‐MEM (HiMedia Labs, Einhausen, Germany) supplemented with 10% ES‐FBS (EuroClone SpA, Pero, Italy) 3 ng/ml bFGF2 (PeproTech, London, UK). The cells were incubated at 37°C in a humidified, 5% CO_2_ atmosphere until fourth passage. When cells reached the maximum confluence (100%), the proliferating medium was substituted with alpha‐MEM without ES‐FBS and bFGF2 for 2 days for quiescence induction.

### 2.2 Induction of stress‐induced premature senescence (SIPS) with x‐ray exposure

Quiescent cells were exposed to 2Gy x‐ray by using a Mevatron machine (Siemens, Milan, Italy) operating at 6 MeV at room temperature (RT). Following irradiation, cells were further cultivated in alpha‐MEM with 0.5% ES‐FBS for 10, 30 and 60 days (10D, 30D and 60D, respectively).

The non‐irradiated quiescent sample (control) was incubated for 10 days in alpha‐MEM with 0.5% ES‐FBS.

### 2.3 Harvest of conditioned media

Conditioned media (CM) from irradiated MSC cultures were harvested 10, 30 and 60 days post‐x‐ray exposure. To this end, cells were extensively washed with PBS1X and plated into a chemically defined serum‐free culture medium for overnight (ON) incubation. Afterwards, CM containing the MSC secretomes were collected and filtered through 0.2 micron Nalgene sterile syringe filters (Thermo Fisher, Waltham, MA, USA). Then samples were centrifuged at 10,000 rpm for 10 min at RT. After this, we discarded the pellets, and collected CM. This procedure was performed to eliminate cellular debris and apoptotic bodies. The collected supernatants were carefully evaluated by Leica DM IL inverted phase contrast microscope (Leica, Wetzlar, Germany) to exclude the presence of residual debris. After this, the collected CM were stored at −80°C until use. As control, we collected CM from unirradiated quiescent MSCs grown for 10 days in alpha‐MEM with 0.5% ES‐FBS.

#### 2.3.1 Cancer cell line cultures and incubation with CM

Caco‐2 (HTB‐37), HCT116 (CCL‐247) and SW480 (CCL‐228) cell lines were obtained (American Type Culture Collection, Manassas, VA, USA) and cultivated according to the manufacturer's instructions.

For evaluation of secretome effects on cancer cell biology, the cancer cells were incubated in the proper culture media supplemented with 50% CM obtained from irradiated MSCs (10D, 30D and 60D) or from control cells (CT) for 10 days at 37°C in a humidified, 5% CO_2_ atmosphere. The media were replaced three times during this incubation period.

### 2.4 Peripheral blood mononuclear cells (PBMCs) isolation

PBMCs were obtained from healthy voluntary male donors (25–40 years of age) after informed consent. The procedure was approved by Campania University Ethical Committee (Prot. 0029471/i 14/10/2021). Cells were isolated according to our previously published protocol.^8^ In detail, the cells were separated on a Ficoll density gradient (GE Healthcare, Milan, Italy), and the mononuclear cell fraction was collected and washed in PBS. The resulting cell pellet (PBMCs) was resuspended in a culture medium containing DMEM‐HAM F12 (HiMedia, Einhausen, Germany) supplemented with 1% FBS (EuroClone, Pero, Italy) with or without CM (60D) and incubated at 37°C in a humidified, 5% CO_2_ atmosphere O.N. before their use in Caco‐2 cultures.

### 2.5 Caco‐2 and PBMCs co‐cultures

Caco‐2 cells were plated in a T25 flask at 4000 cells/cm^2^ and O.N. incubated with their growth medium. Then, the culture medium was carefully aspirated, and the Caco‐2 cells were cultivated in fresh medium containing PBMCs (40,000 cells/cm^2^), in this way the PBMCs/Caco‐2 cells ratio was 10:1. This ratio was selected according to previous findings.^9^ The co‐cultures were incubated for 5 days at 37°C with or without 50% D60 secretome supplementation.

### 2.6 Soft agar assay

We evaluated the morphological transformation of cells with soft agar assay for colony formation, which is an anchorage‐independent growth assay in soft agar, according to Wallert and Provost Labs.^10^ In detail, the cells were seeded in 0.5 ml DMEM supplemented with 0.35% agarose (Sigma–Aldrich, St. Louis, MO, USA) and 20% FBS (EuroClone, Pero, Italy) in 35 mm Petri dishes pre‐treated with 0.5% agar (Sigma–Aldrich) in DMEM with 20% FBS. After 21 days of incubation, the cells were centrifuged at 2000 rpm. Then the pellets were fixed in 100% methanol for 10 min at −20°C. Colonies were then stained with 0.01% (w/v) crystal violet (Sigma–Aldrich) in 25% methanol for 30 min. Subsequently, the cells were washed with PBS three times and resuspended in 100% methanol for 30 min. Colony number was determined by manual inspection.

### 2.7 Migration/invasion assay

The migration experiment was carried out according to Zhu and co‐workers.^11^ In detail, 4 × 10^4^ cells were seeded into the upper chamber of a Transwell insert (8‐mm pore size; Corning Inc., Corning, NY, USA) in a serum‐free medium. A medium containing 20% FBS was added in the lower chamber as a chemoattractant. Forty‐eight hours later, the Transwell chamber was discarded, cells were fixed with methanol for 30 min and stained with 0.1% crystal violet for 20 min. The dye that was absorbed by cells was then released in a 100% methanol solution. The released dye was quantified at 595 nm optical density (OD) with an Infinite 200 microplate reader (Tecan, Männedorf, Switzerland).

### 2.8 Cell cycle analysis

For each analysis, 5 × 10^4^ cells were collected by trypsin treatment, and then cells were fixed in 70% ethanol O.N. at −20°C. The samples were then washed with PBS and subsequently dissolved in a hypotonic buffer containing propidium iodide (Sigma–Aldrich) and RNase A (Thermo Fisher, Waltham, MA, USA). The samples were analysed using a Guava Muse Cell Analyzer (Millipore, Burlington, MA, USA), following the manufacturer's instructions.

### 2.9 Flow cytometry analysis of PBMCs

The isolated PBMCs were analysed on a BD FACSLyric Flow Cytometry System (BD Biosciences, Franklin Lakes, NJ, USA) with BD Multitest TM 6‐Colour TBNK, according to the manufacturer's instructions.

### 2.10 Annexin V assay

Apoptotic cells were detected using a fluorescent Annexin V kit (Dojindo Molecular Technologies, Rockville, MD, USA) on a Guava Muse Cell Analyzer flow cytometer following the manufacturer's instructions.

### 2.11 EdU staining

DNA synthesis monitoring was performed using an EdU Click 488 kit assay (Base Click, Neuried, Germany), according to the manufacturer's instructions. Nuclear staining was performed using DAPI mounting medium (ab104139, Abcam, Cambridge, UK), and micrographs were performed with a Leica DM2000 fluorescence microscope and a DMC5400 camera (Leica, Wetzlar, Germany).

### 2.12 Immunocytochemistry (ICC) and senescence‐associated beta‐galactosidase

We carried out a beta‐galactosidase assay with ICC against Ki67 and pRPS6, as previously described.^12^ Briefly, 2 × 10^4^ cells per well were seeded in 24 wells with glass coverslips. The cells were then fixed in a 2% formaldehyde solution for 10 min. Then, cells were incubated at 37°C O.N. with a beta‐galactosidase staining solution.^12^ Cells were then permeabilized with 0.3% Triton X‐100 (Roche, Basel, Switzerland) and incubated with the antibodies against pRPS6 (1:1000, 4858, Cell Signaling, Danvers, MA, USA) and Ki67 (1:200, sc7846, Santa Cruz Biotechnology, Santa Cruz, CA, USA) at 4°C O.N. Nuclear staining was performed using DAPI mounting medium (ab104139, Abcam, Cambridge, UK), and micrographs were produced with a fluorescence microscope and camera (Leica, Wetzlar, Germany). For every marker we analysed, the percentage of positive cells was calculated by the number of cells that expressed the specific marker stain out of at least 500 cells in different microscope fields.

### 2.13 ICC against pH2A.X and pATM

2 × 10^4^ cells per well were seeded in 24 wells with glass coverslips. Cells were then fixed in a solution of 4% formaldehyde for 10 min and permeabilized with 0.3% Triton X‐100. Samples were subsequently incubated with the antibodies against pH2A.X (Ser139) (1:600, 2577, Cell Signaling, Danvers, MA, USA) and pATM (S1981) (1:1000, ab36810, Abcam, Cambridge, UK) at 4°C O.N. in buffer solution (PBS1X, 5% BSA and 0.1% Triton X‐100). Nuclear staining was performed using DAPI mounting medium (ab104139), and micrographs were produced with a fluorescence microscope and camera (Leica, Wetzlar, Germany). For the pATM we analysed, the percentage of positive cells was calculated by the number of cells that expressed the specific marker stain out of at least 500 cells in different microscope fields. For pH2A.X, we counted the number of positive dots for every cell nucleus.

### 2.14 Western blot analysis

Cells were lysed in a buffer containing 0.1% Triton X‐100 for 30 min in ice. 20 μg of protein for each sample were electrophoresed in a polyacrylamide gel and electroblotted onto a nitrocellulose membrane. We used the following primary antibodies: RB1 (AV33212) and GAPDH (G8795) (Sigma–Aldrich); RB2/P130 (R27020) (BD Biosciences, Allschwil, Switzerland); and P27KIP1 (3686) (Cell Signalling Technology, Danvers, MA, USA), as well as P53 (sc‐126) and P21CIP1 (sc‐397) (Santa Cruz Biotechnology, Santa Cruz, CA, USA) and P16INK4A (ab54210) (Abcam, Cambridge, UK). Immunoreactive signals were detected with a horseradish peroxidase‐conjugated secondary antibody (ImmunoReagents, Raleigh, NC, USA) and reacted with ECL plus reagent (Merck Millipore, Burlington MA, USA). The mean value was quantified densitometrically using Quantity One 1‐D analysis software (Bio‐Rad, Hercules, CA, USA).

### 2.15 mRNA extraction and real‐time quantitative polymerase chain reaction (real‐time qPCR)

Total RNA was extracted from cells using TRI Reagent (Molecular Research Center Inc., Cincinnati, OH, USA). The RNA quantification was performed with a NanoDrop Spectrophotometer (Thermo Scientific, Waltham, MA, USA). The primer pairs for real‐time qPCR used in this paper were designed using OligoArchitect (Sigma–Aldrich). An appropriate region of GAPDH mRNA was used as an internal control. Supplementary file S5 reports the list of the primers used. Real‐time PCR assays were carried out three times on a LineGene 9600 Plus (Bioer, Binjiang, Hangzhou, China). Reactions were performed according to the manufacturer's instructions. SYBR green PCR master mix (abm, Richmond, BC, Canada) was used and the 2^−ΔΔCT^ method was employed as a relative quantification method.

### 2.16 Sample preparation for mass spectroscopy

Irradiated and control MSC cultures were incubated in serum‐free media for 24 h. Subsequently, culture media (secretomes) were collected from each sample. Culture debris were eliminated by centrifugation at 10,000 g for 10 min, and supernatants were used for the StartaClean beads protein pooling according to a procedure we have already described.^13^

### 2.17 LC–MS/MS (liquid chromatography‐mass spectrometry/mass spectrometry) analysis

LC–MS/MS analysis was performed with a Triple ToF 5600+ (AB Sciex, Framingham, MA, USA) that was associated with an LC–MS/MS Eksigent Ekspert NanoLC 400 System (AB Sciex). The peptide mixture was separated by a nanoACQUITY UPLC 1.8 μM HSS T3 C18 column (Thermo Fisher, Waltham, MA, USA) in the trap‐elute mode. A 4–40% ACN gradient was used for 240 min to separate the peptides. Data dependent acquisition (DDA) MS/MS analysis of separated peptides was performed after electrospray ionization. For each sample, the raw data analysis and multiple analytical data measurements were performed with Analyst TF v.1.6 (AB Sciex). The peptides and the ion‐product of the MS and MS/MS data were evaluated with PeakView (AB Sciex). Generated peak‐lists were evaluated in consideration of the UniProtKB‐based reference library of the *Homo sapiens* species on our server with ProteinPilot 4.5 Beta (AB Sciex).

### 2.18 Gene ontology (GO), REACTOME and network analysis

The protein content of secretomes was evaluated with PANTHER gene ontology (http://www.pantherdb.org), with the REACTOME (https://REACTOME.org) and with NETWORKANALYST (https://www.networkanalyst.ca).

PANTHER allowed the GO analysis by classifying protein contents according to “biological process” ontological terms.^14^ For PANTHER analysis, we used the statistics overrepresentation, which compares classifications of multiple clusters of lists with a reference list to statistically identify the over‐ or underrepresentation of PANTHER ontologies. Significance was set to a *p* value of 0.05.

The secretome proteins were mapped to specific pathways with REACTOME analysis. We carried out an overrepresentation and a pathway‐topology analysis. Overrepresentation analysis determines whether certain specific REACTOME pathways are enriched in the submitted protein dataset. This analysis produced a probability score, wherein the false discovery rate (FDR) was corrected for using the Benjamini‐Hochberg method. We followed the developers' instructions for running a REACTOME analysis.^15^, ^16^

The building of a protein network with NETWORKANALYST allows a clear resolution of the biological context of the analysed proteins. The most relevant biological pathways and molecules that interact with the protein list of interest are assembled into a functional whole.^17^ We generated protein networks with a Minimum IMEx Interactome Network, which curtails the networks and holds only seeds and their connecting nodes. Within the generated networks, we selected only nodes having a node degree >20 and betweenness >200.

### 2.19 Statistical analysis

Statistical significance was evaluated using ANOVA followed by Student's *t* and Tukey tests. All data were analysed using the GraphPad Prism version 5.01 statistical software package (GraphPad Software, San Diego, CA, USA).

## 3 RESULTS

Several findings on senescence have been performed on proliferating cells that become senescent cells following a stressful stimulus. Nevertheless, in our body many cells are in a quiescent state and hence it is important to evaluate effects of genotoxic clues on resting cells.

In a previous finding, we demonstrated that, following 48 hours serum starvation, the great majority of MSCs in culture left cell cycle and entered quiescence.^12^ We induced quiescence of MSCs with serum starvation and then irradiated them with 2 Gy x rays to cause senescence. Cells were further cultivated up to 60 days in conditions that preserved quiescence status but provided minimum nutrients and healthy conditions (0.5% serum supplement).

### 3.1 Quiescent MSCs entered senescence following x‐ray exposure

We performed biomolecular analyses at 10, 30 and 60 days post‐irradiation. Hereinafter, these time points are designated as 10D, 30D and 60D, respectively. As a reference, we used unirradiated cells at 10 days of cultivation in “quiescence medium” (CT). We did not use unirradiated cells at 30 days and 60 days post‐quiescence onset, since prolonged cultivation of quiescent cells induces senescence and this can introduce a bias in our reference system. Indeed, long term quiescent cells may enter senescence by reduction of lysosomal function and/or other mechanisms, such as oxidative stress.^1^, ^2^, ^18^

**FIGURE 1:**
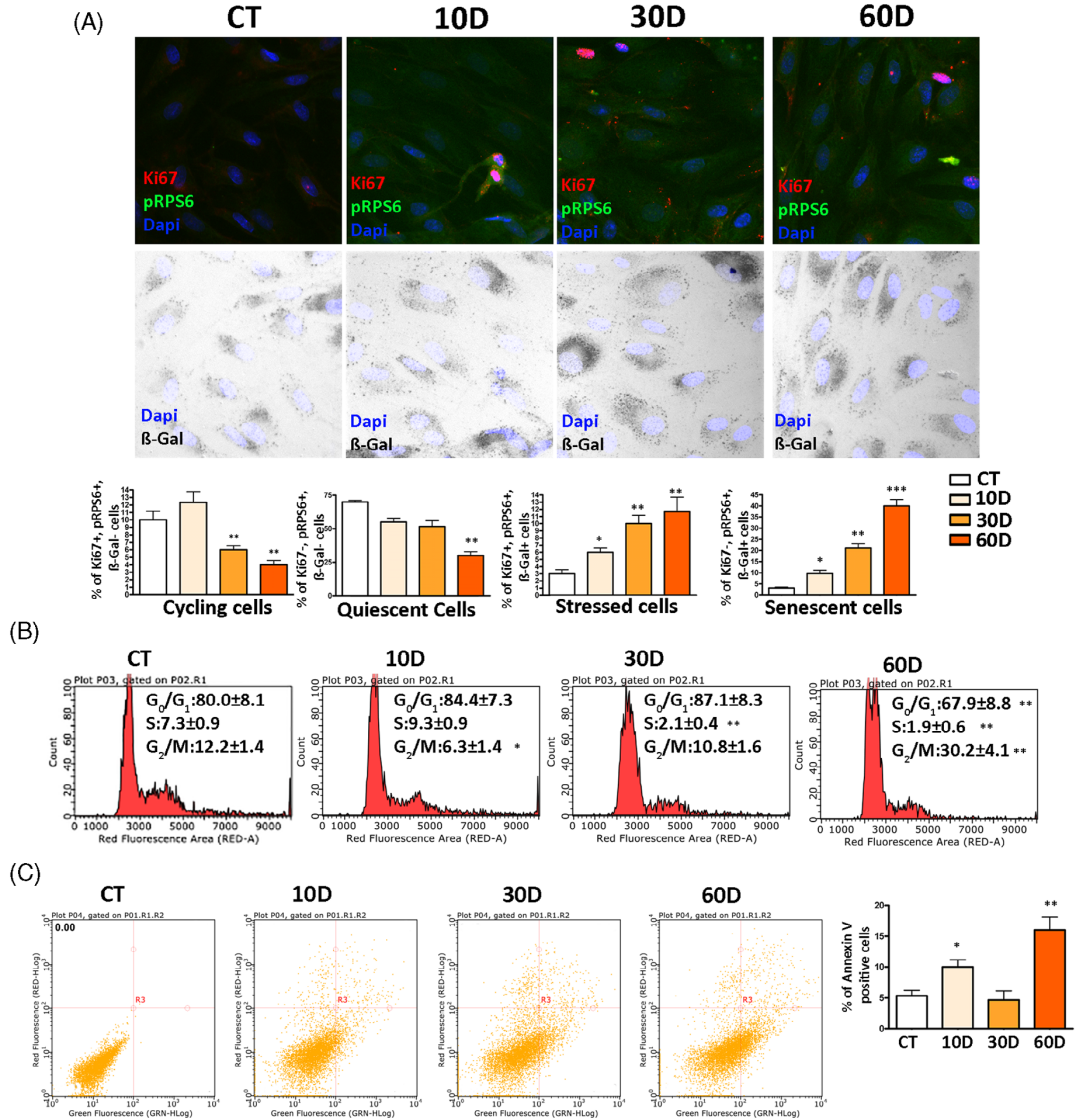
Biological parameters of MSCs following x‐ray exposure. Panel A: representative images of cells stained to identify nuclei (DAPI), pRPS6, Ki67 and to determine β‐gal activity. The graphs show the percentage of cycling, quiescent, stressed and senescent cells. In each graph, the * indicates the statistical difference between CT, chosen as the reference, and the other time points. Data are shown with standard deviation (SD), *n* = 3 (*p < 0.05, **p < 0.01, ***p < 0.001). We used Leica CTR500 microscope equipped with a DCF3000G digital monochrome camera. The β‐gal activity was recorded as a grey‐stain with this setting. This experimental approach was used to identify in the same cell, a marker emitting a signal in the visible light (β‐gal) together with others expressing fluorescent signals. Panel B: Cell‐cycle profiles of samples collected at different time points following x‐ray treatment. The * indicates the statistical difference between CT, chosen as the reference, and the other time points. Data are shown with standard deviation (SD), *n* = 3 (*p < 0.05, **p < 0.01). Panel C: Flow cytometry chart of annexin V assay. The percentage of apoptotic cells is indicated in the associated right histogram. Data are shown with standard deviation (SD), *n* = 3 (*p < 0.05 and **p < 0.01 indicate statistical significance between the control and treated samples).

We identified cycling, quiescent and senescent cells by the algorithm we set up previously.^12^ The cycling cells were positive for Ki67 and phosphorylated ribosomal RP6 (pRP6). At the same time, they were negative for senescence‐associated acid beta‐galactosidase (β‐gal). The quiescent cells were Ki67(−), pRP6(−) and β‐gal(−), while the senescent cells were Ki67(−), pRP6(+) and β‐gal(+). At 10D, in reference cultures around 90% of cells were not positive for cycling markers and about 4% of these cells were senescent (Figure 1A). In irradiated culture, we detected around 10% of senescent cells at 10D. This percentage further increased at 30D and 60D and replaced the percentage of quiescent cells (Figure 1A). This result agreed with cell‐cycle analysis showing a progressive reduction of cells in S‐phase and an increase of those in G2/M phase in irradiated cultures (Figure 1B).

There are findings showing that within a few hours after genotoxic damage cells show a rapid increase in SA‐β‐gal activity. This event does not pinpoint a senescent phenotype; rather, it evidences stressed cells, which can either successfully cope with the injury and recover a functional status or wilt and enter senescence. In our previous study we showed that stressed cells were Ki67(+), pRPS6(+) and SA‐β‐gal(+).^12^ The stressed cells show increased SA‐β‐gal activity but are still positive for the Ki67 cycling marker. In irradiated cultures, we observed an increase in stressed cells, besides augmentation of senescent ones (Figure 1A).

Following genotoxic damage, MSCs are generally more prone to senescence than apoptosis.^19^ Nevertheless, the percentage of apoptotic cells at 10D in irradiated cultures changed significantly compared with the control sample (Figure 1C). A huge increase of apoptosis was observed at 60D. This augmentation of dead cells may be due to prolonged cultivation with minimal serum supplement, or, alternatively, cultures at 60D were approaching a final post‐senescence stage with degradation phenomena and cell death.^20^

**FIGURE 2:**
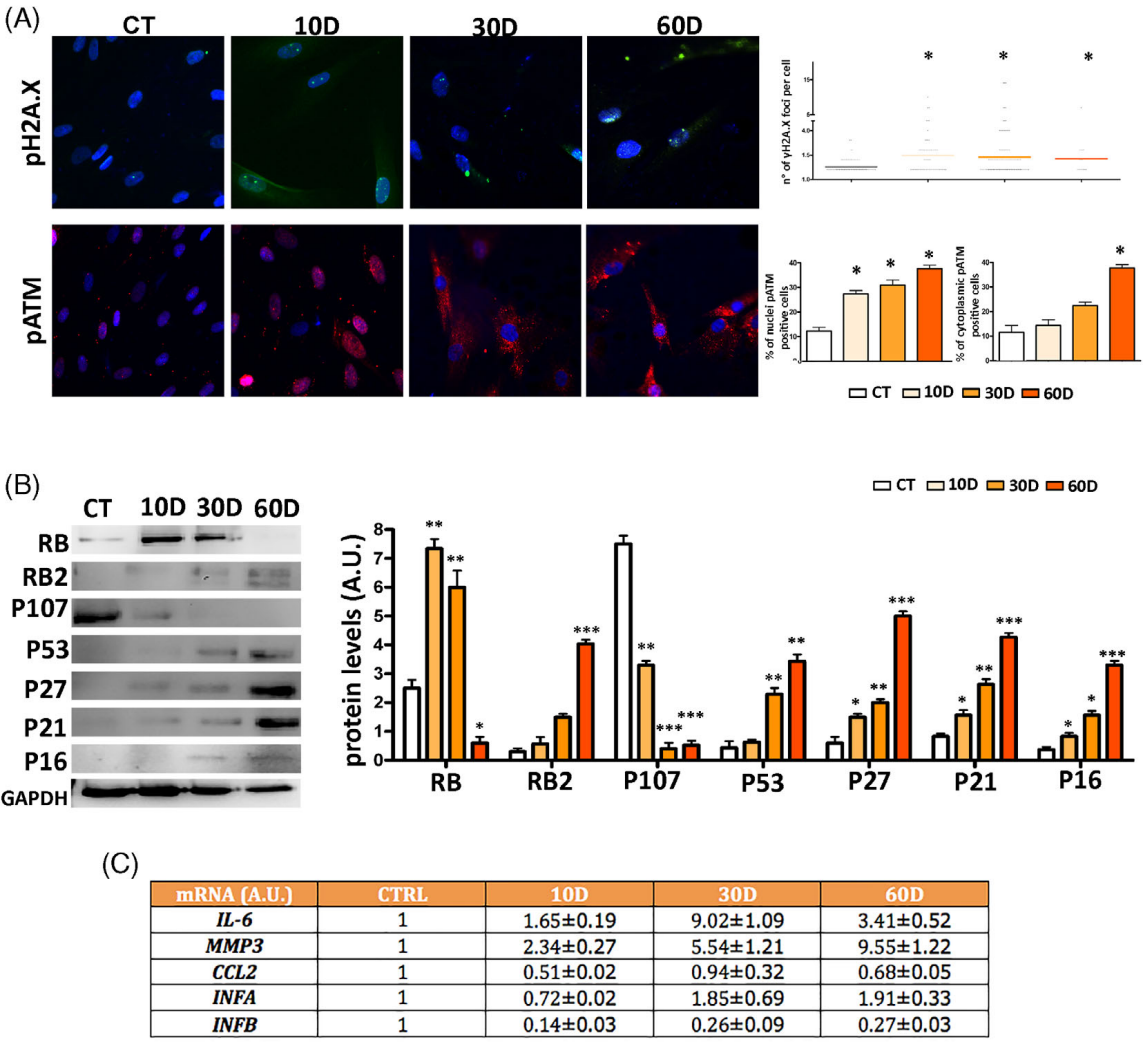
DNA damage/repair analysis and senescence‐associated signalling pathways. Panel A: Representative images of cells stained with anti‐pH2A.X (green) or pATM (red) are shown. Cell nuclei were stained with DAPI. Upper right: the graph shows the degree of pH2A.X foci per cell (*n* = 3 ± SD; *p < 0.05). The lower right graphs show the percentage of pATM positive cells. The protein is localized either within the nucleus or into cytoplasm (*n* = 3 ± SD; *p < 0.05). Panel B: A representative western blot analysis of RB/P105, RB2/P130, P107, P53, P27/KIP1, P21/CIP1, P16/INK4A and GAPDH (loading control) is shown. The right histogram reports the densitometric analysis of western blot bands. Panel C: The table reports the mRNA expression level of the indicated genes. The mRNA levels were normalized to GAPDH mRNA expression, which was selected as an internal control. For every mRNA, the change in the expression level is compared with control culture (CTRL), whose expression was fixed as 1. The symbols ***p < 0.001, **p < 0.01 and *p < 0.05 indicate statistical significance between the control and irradiated samples.

Senescence is associated with the presence of unrepaired/misrepaired DNA damage and aberrant persistent activation of DNA damage‐repair (DDR) machinery.^5^, ^21^ At 10D, in irradiated MSCs, we detected an increase in phosphorylated H2A.X (pH2A.X) in nuclei compared with control (Figure 2A). The pH2A.X, which marks damaged DNA, persisted at 30D and 60D. Damaged DNA was associated with a persistent DDR signalling through activated ATM in cell nuclei (Figure 2A). Of note, an activated ATM, which is localized in the cytoplasm, can contribute also to the acquisition of a proinflammatory SASP.^22^ We found an increase in the percentage of cells with activated cytoplasmic ATM in irradiated cells at 30D and 60D (Figure 2A).

The executive senescence program relies on the P53 and/or retinoblastoma pathways depending on cell type and animal species.^23^ In irradiated cultures, at 30D and 60D, we observed a strong increase of P53 compared to reference culture (Figure 2B). The initial stages of senescence relied on RB upregulation that progressively declined at 60D and was accompanied by an increase of RB2/P130 protein (Figure 2B). The P107 protein, another member of the retinoblastoma family, showed a significant decline in all the irradiated samples. Of note, the levels of cyclin kinase inhibitors (CKI): P16, P21 and P27 were upregulated in senescent cultures and progressively upregulated as senescence entered into its final stages (Figure 2B; Supplementary file S5).

### 3.2 SASP from irradiated MSCs progressively accumulated pro‐inflammatory factors during in vitro cultivation

The above‐reported results indicated that in our experimental conditions the irradiated quiescent MSC cultures enter progressively into late/deep senescence. This data is in line with other findings showing that deep senescence can be reached within 20–30 days following a genotoxic injury.^24^, ^25^, ^26^ Progression from early to late senescence is associated with SASP enriched in pro‐inflammatory factors. De Cecco and co‐workers identified some factors (IL6, IFNA, IFNB, CCL2, MMP3) whose mRNA levels can be considered as good markers to ascertain passage to the late‐senescence stage.^27^ Irradiated MSCs showed a continuous increase in IFNA and MMP3 going from 10D to 60D (Figure 2C). In contrast, IL6 peaked at 30D and then declined, while IFNB and CCL2 did not reach levels above those observed in reference samples (Figure 2C). Collectively, these results evidenced that SASP could be enriched in some pro‐inflammatory factors during in vitro cultivation of senescent cells.

### 3.3 The onset and establishment of senescence were associated with significant changes in SASP composition

**FIGURE 3:**
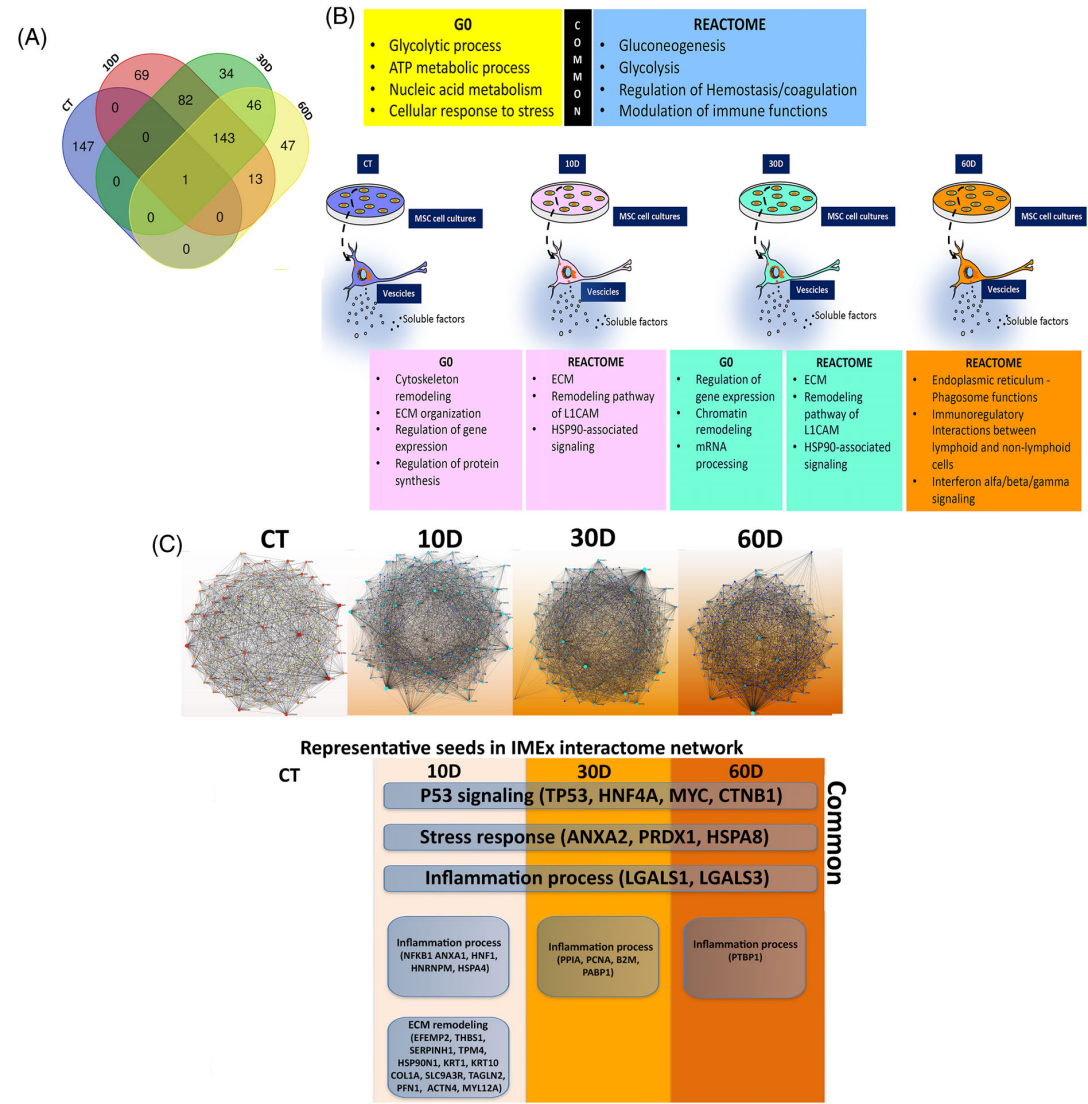
Gene Ontology and REACTOME analysis. Panel A: Venn diagram to identify common and specific SASP components among the different experimental conditions. Panel B: main GO and REACTOME outcomes. The SASP contained both factors, which were common among control and irradiated samples (see upper boxes), and proteins that were specifically present in a given sample (see lower boxes). Our analysis was carried out on whole secretomes containing soluble factors and molecules encapsulated within extracellular vesicles. Panel C: Network analysis. The top pictures show Minimum IMEx Interactome Networks with reduced complexity obtained by considering only seeds and their connecting nodes. The bottom images show representative seeds (node degree >20 and betweenness >200). Common: indicates seeds that are present in all irradiated samples (10D, 30D, 60D).

We then decided to gain more insights into secretome composition obtained from irradiated MSCs to evaluate changes during progression to deep senescence. We performed a LC–MS/MS analysis of protein secretome content in the different experimental conditions. We identified 148 proteins in control quiescent cultures, while in irradiated samples we found 309, 306 and 249 at 10D, 30D and 60D, respectively (Supplementary file S1). We then performed a Venn analysis to identify which factors were exclusively present in a given condition and which were not. Of note, 147 of 148 proteins identified in control samples were not present in irradiated ones. This result indicates that senescence, following irradiation, induced a drastic change in the secretome composition (Figure 3A, Supplementary file S1). The data is in line with the onset of senescence‐specific biological activities. The irradiated samples contained a core of 143 common factors, while the others were specifically present in each of the three irradiated conditions or were in common between two of them (Figure 3A, Supplementary file S1).

We parsed the different secretomes by Gene Ontology (GO) to determine the relative frequency of ontological terms associated with specific cellular functions. We performed analysis of the terms grouped in the GO biological process database and found 100 ontologies for each of the four experimental conditions (Supplementary file S2).

Venn analysis identified 23 common ontologies among all the samples, in spite of significant differences in secretome content between control and irradiated cultures. Most of the common ontologies refer to fundamental cellular processes, such as glycolytic and other metabolic processes, nucleic acid anabolism/catabolism and cellular response to stress (Figure 3B, Supplementary file S2).

The secretome of irradiated cultures at 10D was enriched in several ontologies associated with cytoskeleton and extracellular matrix (ECM) remodelling, as expected for cells entering senescence, which have a reshape of cell phenotype. In irradiated samples at 10D we also found ontologies associated with regulation of gene expression and protein synthesis. Indeed, senescent cells must implement specific programs for permanent cell‐cycle exit, response to stress and secretome production.^3^, ^5^, ^19^ Senescent cells also have an active protein synthesis compared to quiescent cells. Several ontologies related to regulation of gene expression were also identified in 30D irradiated samples. In detail, some ontologies were associated with regulation at transcriptional (chromatin remodelling) and post‐transcriptional levels (mRNA processing) (Figure 3B, Supplementary file S2).

GO analysis evidenced profound changes in biological processes following onset and setting up of senescence. We then identified the set of interactions for each protein in our datasets by REACTOME analysis. This pathway database considers as “reaction” among molecules any binding, activation, translocation, degradation and all other biochemical phenomena involving a catalyst.^15^, ^16^ The REACTOME analysis granted us the identification of the most representative pathways, which may have a functional importance for the biological phenomena pinpointed by GO evaluation. The Venn analysis allowed the identification of common and specific pathways among the different experimental conditions (Figure 3B, Supplementary file S3). Of note, pathways related to gluconeogenesis and glycolysis were found in all the secretomes and this is in line with starvation conditions in which cells must produce glucose via non‐carbohydrate substrates. Other common pathways were associated with platelet and neutrophil degranulation. These pathways are typical of paracrine activities of MSCs, which release several factors involved in regulation of haemostasis, coagulation and modulation of immune system functions. We previously found these pathways in the secretome of active proliferating adipose tissue‐derived MSCs indicating that these signalling factors are part of the major paracrine activities of MSCs, irrespective of cell physiological status and tissue of residence.^28^

Two pathways that were exclusively found in the secretomes of 10D and 30D samples evidenced that senescence phenomena completely modified the paracrine signalling of MSCs (Figure 3B, Supplementary file S3). We identified a pathway associated with ECM remodelling, such as the recycling pathway of the L1 cell adhesion molecule (L1CAM). The L1 protein may act either as an adhesion molecule between cells or promote migration during neural development and metastasis formation.^29^ HSP90‐associated signalling was the other pathway specifically identified in the 10D and 30D secretomes. HSP90 proteins are involved in several cellular processes (cell survival, cell‐cycle control, hormone signalling and apoptosis) and may play a role in homeostasis, stress response and several pathological conditions.^30^ As the senescence process proceeded to a late stage, the composition of secretome changed and other signalling pathways were identified. Several pathways were related with endoplasmic reticulum (ER)‐phagosomes and their activities, such as stress response. Of great interest, the paracrine activity of senescent MSCs shifted to pro‐inflammatory signalling, since we found a pathway of immunoregulatory interactions between lymphoid and non‐lymphoid cells, another related to interferon alfa/beta/gamma signalling, and many others associated with antigen processing and presentation (Figure 3B, Supplementary file S3).

By merging the GO and REACTOME analysis data, we had a global outlook to the main functions and pathways implemented in senescent secretome, such as: (i) changes in gene expression; (ii) cytoskeleton and ECM remodelling; (iii) ER‐phagosome activities and stress response; and (iv) inflammation signalling.

By using a top‐down approach, which goes from the general to the specific, we tried to identify the most important factors present in the secretomes and underlying the above‐identified functions and pathways. To this end, we performed a network analysis. The construction of a protein network permits a straightforward resolution of the biological context of the analysed proteins. The NetworkAnalyst database maps the experimental protein datasets to a selected underlying database. This approach generates networks and allows the identification of proteins that directly interact with proteins of the experimental dataset, which are named seeds. We generated networks with the Minimum IMEx Interactome Network, which reduces networks' complexity by keeping only seeds and their connecting nodes. We produced “core networks” by choosing only nodes having a high node degree and betweenness. These two parameters are among the most important network features: the node degree pinpoints the number of edges that connect a node, while betweenness indicates the number of times a node lies on the shortest path between other nodes.

### 3.4 Network analysis of SASP components showed the senescence‐related significant hubs

The networks of irradiated samples showed some common key seeds that are important for implementation of a senescence executive program and regulation of gene expression. In particular there are many factors involved in P53 signalling (TP53, HNF4A, MYC, CTNB1). Other factors are involved in stress response (ANXA2, PRDX1, HSPA8) and others in inflammation phenomena (LGALS1, LGALS3) (Figure 3C, Supplementary file S4).

We found more seeds specifically present in irradiated samples at 10D compared with 30D and 60D, as if at 10D the onset of the senescence process needs the implementation of several signalling circuits. In detail, many seeds were associated with cytoskeleton and ECM remodelling (EFEMP2, THBS1, SERPINH1, TPM4, HSP90N1, KRT1, KRT10 COL1A, SLC9A3R, TAGLN2, PFN1, ACTN4, MYL12A), and others with synthesis, homeostasis, and degradation of proteins and organelles (Figure 3C, Supplementary file S4). In 10D secretomes there were also seeds regulating the inflammation process (NFKB1 ANXA1, HNF1, HNRNPM, HSPA4). The seeds specifically present in SASP from 30D and 60D samples evidenced an enrichment in factors governing inflammation phenomena, such as PPIA, PCNA, B2M, PABP1 in 30D samples and PTBP1 in the 60D ones (Figure 3C, Supplementary file S4).

### 3.5 The SASP coming from cells with different senescent stages showed overlapping paracrine activities on cancer cells

Having evidenced significant changes in secretome composition during the progression of senescence induced by irradiation, we aimed to ascertain how these modifications affected the SASP paracrine functions. We focused our attention on the SASP capacity to arrest the growth of tumour cells by inducing senescence and/or apoptosis. Indeed, there are several findings showing that SASP from deep senescent cells may lose its anti‐tumour properties and, on the contrary, may sustain cancer growth through the presence of pro‐inflammatory factors that modulate immune system activity by creating a favourable tumour microenvironment.^3^, ^5^, ^31^, ^32^

We evaluated SASP paracrine function on three colorectal cancer cell lines: SW480, Caco‐2 and HCT116. The choice was based on the consideration that the mutation patterns of these cell lines are representative of the genetic and epigenetic landscape of this tumour phenotype.^33^ The Caco‐2 cells had microsatellite stability, chromosomal instability, a wild‐type *KRAS*, a wild‐type *PIK3CA* and an E204X mutation in *TP53*. The HCT116 cells had microsatellite instability, chromosomal stability, a G13D mutation in *KRAS*, an H1047R mutation in *PIK3CA* and a wild type *TP53*. The SW480 had microsatellite stability, chromosomal instability, a G12V mutation in *KRAS*, a wild‐type *PIK3CA*, and a R273H, P309S mutations in *TP53*.^33^

**FIGURE 4:**
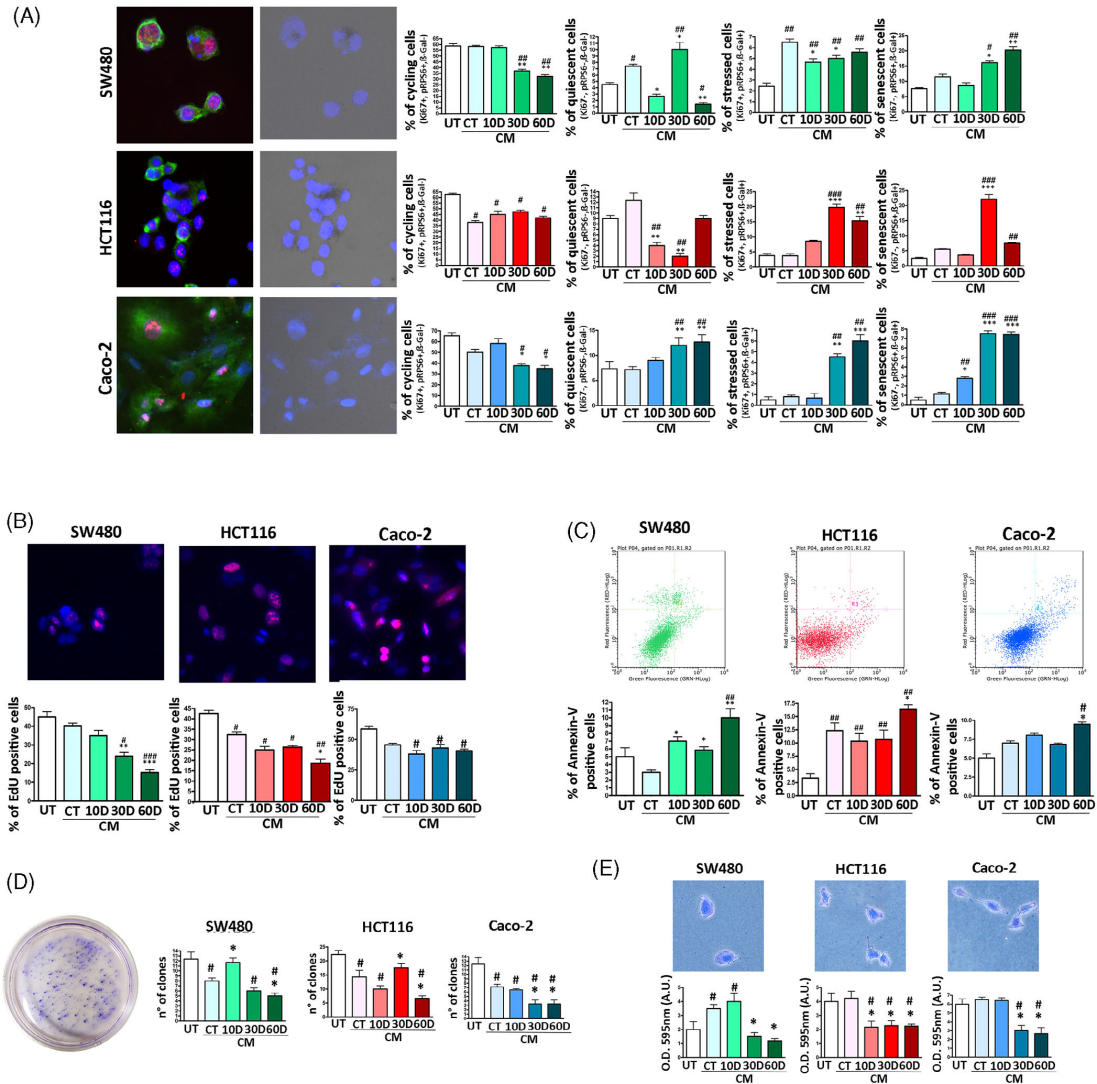
Biological parameters of cancer cells incubated with SASP. Panel A: SW480, HCT116 and Caco‐2 cells were incubated with different MSC secretomes as reported in methods. The histograms show the percentage of cycling, quiescent, stressed and senescent cells following secretome treatment. Representative images of cancer cells stained with DAPI (blue), pRPS6 (green) and Ki67 (red) are reported on the far left. In addition, the same images were analysed under a light microscope to determine β‐gal activity (black dots). Panel B: The histograms show the percentage of EdU positive cells in the different experimental conditions. Representative images of EdU (red) and DAPI (blue) staining are reported. Panel C: The histograms show the percentage of apoptotic cells as detected by flow cytometry Annexin V assay. Representative plots of apoptosis analysis are reported. Panel D: The histograms show the number of clones obtained in colony formation assay and the migration capacity of cancer cells, which was determined by crystal violet cell staining. An example of CFU assay is depicted. Panel E: The graphs show the results of invasion/migration assay performed on, SW480, HCT116 and Caco‐2 cancer cells. The migrated cells were stained with crystal violet and a quantitative evaluation of migration capacity was performed by measuring the optical density (O.D.) of the crystal violet stain at 595 nm. UT are the untreated cancer cells. The CT, 10D, 30D and 60D acronyms indicate cancer cultures incubated with conditioned media (CM) obtained from non‐irradiated (CT) and irradiated MSCs. For all the assays, the symbols ***p < 0.001, **p < 0.01 and *p < 0.05 indicate statistical significance between the CT, chosen as reference, and 10D, 30D, 60D. The symbols ###*p* < 0.001, ##*p* < 0.01 and #*p* < 0.05 indicate statistical significance between the UT, chosen as reference, and the other samples.

We incubated the cancer cells for 10 days in medium supplemented with secretomes of irradiated MSCs (10D, 30D, 60D) and compared their biological parameters with cancer cells incubated with secretome of nonirradiated MSCs (CT) and with the corresponding untreated cancer cultures (UT). The percentage of Caco‐2 and SW480 [Ki67(+), pRP6(+) and β‐gal(−)] cycling cells declined when incubated with SASP from 30D and 60D samples compared with untreated samples (UT) and with CT secretome (Figure 4A). In HCT116, the decline in cycling cells was observed only with respect to UT and not CT (Figure 4A). Data on cycling cells were in concordance with EdU analysis (Figure 4B). The pattern of quiescent cells was more complex (Figure 4A). In Caco‐2, the percentage of quiescent cells increased in samples treated with 30D and 60D secretome compared to UT and CT, while in SW480, the increase was observed only with 30D and declined with 60D. In HCT116, we did not observe an increase in quiescent cells but rather a decline in samples treated with 10D and 30D compared to UT and CT. These data must be globally evaluated by also considering the percentage of senescent and stressed cells (see below).

In SW480 and HCT116 cells, the percentage of senescent cells was low in control condition and increased with 30D and 60D treatment. In HCT116, the increase in senescence with 60D was lower than that observed with 30D incubation (Figure 4A). The Caco‐2 samples showed a very low number of senescent cells (around 1% in UT and CT), but this percentage increased significantly (around 9%) when cultures were incubated with 30D and 60D (Figure 4A). The number of stressed cells increased in all three cancer cell phenotypes following incubation with 30D and 60D (Figure 4A). Of note, HCT116 showed a significant increase in the percentage of apoptotic cells when treated with senescent secretomes, the SW480 evidenced an increase of apoptosis at 10D and 30D and a very significant augmentation at 60D compared with UT (Figure 4C). The Caco‐2 cells showed changes in apoptosis only in 60D treated samples. In some settings, the CT secretome produced some biological effects on cancer cells that are comparable to those induced by 10D, 30D, 60D secretomes. It must be remembered that CT samples contain some senescent cells that can secrete SASP factors.

Loss of cell adhesion and invasion has been classically viewed as tumorigenic features of cancer cells. We then evaluated the effect of SASP on colon cancer cell capacity to grow on soft agar and to in vitro migrate. Generally, almost all the analysed SASPs negatively affected the migration and unanchored growth of the cancer cell lines we analysed (Figure 4D,E). Of note, the 60D secretome induced the most significant impairment of these tumorigenic properties.

### 3.6 The presence of immune cells impaired the anti‐tumour properties of SASP

The observation that SASP coming from deep senescent cells preserved its anti‐tumour properties is at odds with several findings showing that during progression from early to late senescence the SASP will be enriched in pro‐inflammatory cytokines that can foster cancer growth.^4^, ^34^ In this context, it is possible to imagine that SASP always contains some core components that have anti‐tumour activity, the progression from early to late senescence enriches the SASP of pro‐inflammatory factors that may promote its tumorigenic activity only by interacting and instructing cells of the immune system.

We evaluated this hypothesis by treating cancer cells with SASP in the presence of immune cells. We selected the deep senescence secretome (60D) that, in principle, should have the most pro‐tumorigenic properties, but, in our experimental conditions, showed significant anti‐tumour activities especially on migration and unanchored growth of Caco‐2 cells.

**FIGURE 5:**
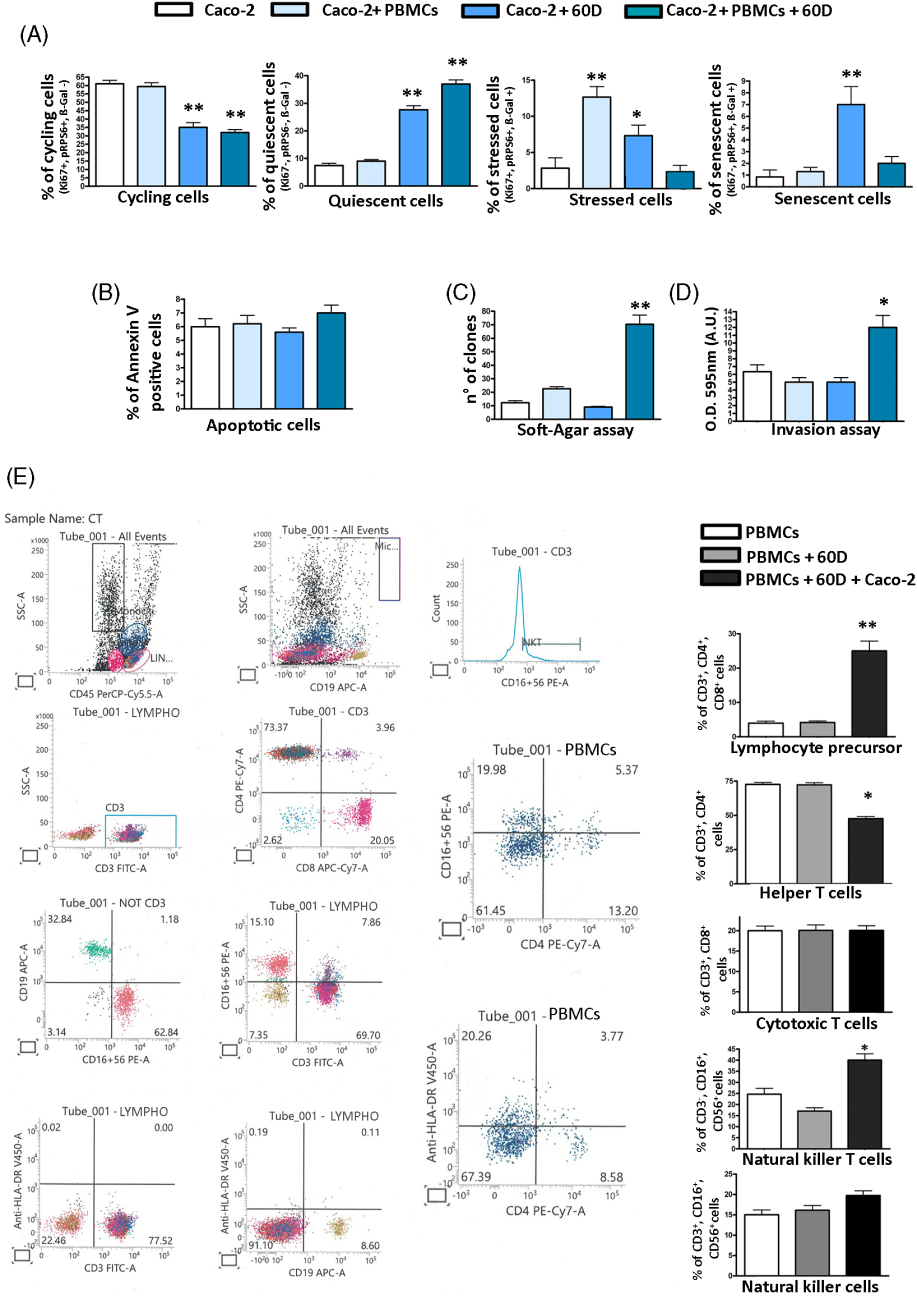
Effects of SASP and PBMCs on Caco‐2 cell biology. Panel A: Caco‐2 cells were incubated with 60D SASP and/or PBMCs as reported in methods. The histograms show the percentage of cycling, quiescent, stressed and senescent cells following this treatment. Panel B, C, D: Apoptosis, soft agar colony formation, and cell migration were evaluated on Caco‐2 cells treated as indicated in panel A. Panel E: representative plots of flow cytometry analysis carried on naïve PBMCs or on those incubated with SASP and Caco‐2 cells. For all the assays, the symbols **p < 0.01 and *p < 0.05 indicate statistical significance between the control and irradiated samples.

In detail, we isolated peripheral blood mononuclear cells (PBMCs) from healthy individuals and incubated them with the 60D SASP for 18 h. Then, Caco‐2 were cultivated in the presence of this “primed” SASP and PBMCs. The PBMCs blunted most of the SASP anti‐tumour properties. In detail, the presence of PBMCs did not increase the percentage of Caco‐2 cycling cells and/or reduced the senescent cells compared to treatment with 60D only but almost eliminated the percentage of stressed cells in favour of an increase in quiescent cells, suggesting that stress events induced by SASP successfully coped with the presence of PBMCs (Figure 5). No significant changes were observed in the apoptosis phenomena. What was more striking was the effect on migration and unanchored growth: the co‐presence of SASP and PBMCs significantly increased the number of migrating cells and of clones growing in soft agar to a level that was higher than the one observed in control samples cultivated without SASP.

These significant changes in biological properties of SASP induced by PBMCs suggested a crosstalk between immunomodulator factors present in SASP, immune cells and cancer cells. Indeed, we performed a very preliminary analysis on the changes in cell phenotypes observed following incubation of PBMCs with SASP and Caco‐2 cells. The co‐presence of SASP and cancer cells induced the most significant modification in the PBMC cell composition. We detected an augmentation of T‐helper/cytotoxic precursors (CD3+, CD4+, CD8+) and of NKT (CD3+, CD16+, CD56+) cells together with a decrease of T‐helper cells (CD3+, CD4+) (Figure 5).

## 4 DISCUSSION

Genotoxic stimuli may induce damage to DNA and other macromolecules and this occurrence may trigger the cellular senescence. The study of this phenomenon is quite challenging, since it depends on several factors and, indeed, many aspects of senescence are not completely understood. Several genotoxic injuries converge on the senescence‐executive program that rely on P53/RB, MAPK pathway, cGAS/STING, CEBPβ and NFκB signalling. These pathways promote cell‐cycle arrest, chromatin remodelling with the onset of a senescence‐related gene expression pattern and production of SASP.^4^, ^5^ The noxious agent triggers random damage to cellular macromolecules and hence, in spite of a common executive program, the senescence features may vary cell by cell. Another puzzling issue must be added to the above complex phenomenon: senescence is a dynamic process and cannot be analysed as a static endpoint.

The study of SASP is particularly intriguing since through it, a senescence process triggered in a few cells can spread to many other cells and produce either beneficial or negative consequences for health. Most SASP analyses have been performed on proliferating cells, while it is well known that many cells are in a quiescent state in our organism. Our findings tried to contribute to fill this gap. We decided to analyse the SASP of quiescent MSCs following stress‐induced premature senescence (SIPS) by ionizing radiation exposure. Our research approach also allowed us to evaluate in a more rigorous way the composition and biological effects of SASP coming from SIPS in the absence of any replicative senescence phenomena. Indeed, if genotoxic damage is induced in proliferating cultures, the events coming from SIPS will overlap with those arising from replicative senescence when cultures are prolonged for extended periods of time.

Several findings on SASPs focused their attention on specific components, such as ECM remodelers, survival and growth factors. In particular, many investigations on the SASP evaluated the levels of cytokines, interleukins and other modulators of inflammation by means of ELISA assays or antibody dot blot assays. These studies, even of great interest to identify specific SASP factors, do not allow an unbiased analysis of SASP composition. The LC–MS/MS detection of SASP components, followed by bioinformatics analyses, allowed us to perform a hypothesis‐free manner study on SASP. It should be underlined that every experimental method has pros and cons. The LC–MS/MS may hold a bias toward high abundance proteins with loss of information about low abundance proteins.^35^ Moreover, the dynamic range of LC MS/MS analysis generally spans from 4 to 6 logarithms orders of magnitude, while proteins in bio‐fluids can span till to 12 logarithms in concentration.^35^ These caveats may render uninformative a quantitative MS study on SASP. We used an experimental approach to minimize such hindrance. Our study aimed at identifying SASP features that were exclusive for every experimental condition. For this reason, the identification of ontologies, pathways and networks was parsed by Venn analysis to identify specific SASP characteristics for every experimental condition (10D, 30D, 60D), rather than quantitative evaluation of changes occurring in the expression of a given factor.

The bioinformatics evaluation of SASPs (GO, REACTOME and Network analysis) showed some common and specific features among 10D, 30D and late 60D SASP. The common ontologies present in the three SASPs refer to biological processes associated with protein depolymerization and organelle organization. This is in line with profound morphological and functional changes occurring in senescent cells compared with healthy ones, as we already evidenced in a previous finding.^36^ In 10D SASP, there are ontologies specifically related to cytoskeleton remodelling, stress and chemical response. This data may suggest that in the early stages of senescence cells have to set up a proper and effective reaction to cope with an injury event. In 30D SASP, the specific ontologies belong to chromatin remodelling phenomena as expected for permanent activation of a senescence‐specific gene expression profile.^37^ In the 30D and 60D SASPs, we found ontologies encompassing metabolic processes involving organic acids, carbohydrates and small molecules. These events are in line with changes in metabolism, which are essential for both inflammatory and anti‐inflammatory responses.^38^

Merging the GO and REACTOME analyses evidenced that in all the senescent secretomes the most significant pathways belong to four main cellular activities: modulation of gene expression, remodelling of cytoskeleton and ECM, stress response and related ER‐phagosome activities and inflammation. It is noteworthy that LC MS/MS may fall detection of specific inflammation‐related cytokines (such as IL‐1, IL‐6, TNF‐α). These factors are present in extracellular fluids at very low concentrations, which are below the MS limit of detection. Nevertheless, the bioinformatics tools helped us to overcome this limit. Indeed, the bioinformatics analyses clearly evidenced a key role for inflammation‐related ontologies and networks in SASPs of our experimental model.

In detail, the 30D and 60D SASPs, which are secretomes of late senescent cells, are enriched in interferon pathways, which play a main role in inflammation phenomena. The senescence process relies on an executive program for its implementation. Of note, several factors belonging to pathways that are associated with this program, such as P53 signalling, stress response and inflammation, are also present in the SASP, as evidenced by Network analysis. These results further strengthen the hypothesis that senescence heavily depends on autocrine/paracrine signalling.

A manual inspection of secretome content showed that SASP of 30D and 60D senescent cells was particularly enriched in proteins involved in modulation of inflammation phenomena that can be exploited by cancer cells for their growth and survival. Besides the well‐known cytokines, some other key factors were identified: HSPA9/MORTALIN, B2M, PABP1 and PTBP1. The MORTALIN is a heat shock protein that has a fundamental role in protecting cells from cytotoxic death (CTD), and its inactivation sensitizes cancer cells to CTD. MORTALIN binds and inactivates the C5b‐9 membrane attack complex (MAC) during complement activation. This will avoid CTD of cancer cells as evidenced for erythroleukemia cells.^39^ MORTALIN has also a role in promotion of colorectal cancer cell proliferation and epithelial‐mesenchymal transition (EMT).^40^ Of note, several evidences showed that modifications of B2M expression dampens antigen presentation and contribute to poor reaction to cancer immunotherapies.^41^ Increased circulating levels of β2‐microglobulin (B2M), a component of major histocompatibility complex class 1, have been associated to aging phenomena, such as cognitive impairment and neurodegeneration.^42^

The PABP1 and PTBP1 are proteins involved in regulation of mRNA half‐life and splicing, respectively. Their expression appears related to global transcriptome changes in senescent cells to promote inflammatory response.^43^, ^44^ In particular, PTBP1 regulates several pathways that play a key role in cancer growth and survival by modulating apoptosis and proliferation through alternative splicing of MCL1 and ADAR1. PTBP1 also contributes to colorectal cancer cells metastasis through downregulation of ATG10. Noteworthy, CDC42 and SASP play a role in tumorigenesis through PTBP1. The isoforms of CDC42, which are the key factors for filopodia formation in tumorigenesis, are generated by alternative splicing regulated by PTBP1. There are studies showing that the inactivation of PTBP1 can impairs the pro‐tumorigenic effects of SASP by modulating immune surveillance.^45^

The presence of global regulators of inflammation‐related gene expression indicates that this phenomenon has a major part in SASP duties of late senescent cells. Globally, these results, and other findings showing that late senescent SASP is enriched in cytokines that could foster cancer growth, are at odds with our data showing 30D and 60D SASPs preserved their anti‐tumour properties in our experimental conditions.

This contradictory result may be explained by hypothesizing that in our conditions we did not reach a deep senescence state. Indeed, we demonstrated that, 30 days after SIPS, the secretome of MSCs contained pro‐inflammatory cytokines and factors associated with deep senescence status. Moreover, 60 days after SIPS, we detected pathways related with IL1‐α and IL‐β that De Cecco and others showed were associated with the final stage of senescence.^27^ In the SASP at 60 days after SIPS, we found the PTBP1 protein, which is involved in the inflammatory and pro‐tumorigenic activity of SASP.^43^ On the other hand, the cancer cell lines we chose may be unresponsive to SASP's pro‐tumorigenic effect. This may occur for one cell line, but it is difficult to hypothesize that three different cell lines were SASP‐insensitive. It must be remembered that the colon cancer cell lines we selected had different epigenetic and genetic profiles and recapitulate the most common colon cancer molecular signature. An alternative hypothesis could reconcile the contradiction between our data and other published findings. The pro‐tumorigenic activity of SASP has been demonstrated in in vivo experiments.^31^, ^32^ The most convincing study on the pro‐tumorigenic activity of senescent cells comes from Eggert and colleagues, which found that the SASP of senescent hepatocytes, coexisting with liver cancer cells, may recruit immature myeloid cells and may promote cancer progression by impairing the function of immune cells, such as NK cells.^31^ In this context, it is possible to imagine that SASP always contains some core components that have an anti‐tumour activity, the progression from early to late senescence enriches the SASP of pro‐inflammatory factors that may promote SASP tumorigenic activity only by interacting and instructing cells of the immune system (See graphical abstract).

Our results on Caco‐2 cells incubated with 60D SASP in the presence of peripheral PBMCs lend support to this hypothesis. Indeed, the presence of PBMCs greatly reduced the percentage of stressed cells in favour of an augmentation of quiescent cells. Furthermore, the incubation of Caco‐2 cells with SASP and PBMCs significantly increased the number of migrating cells and of clones growing in soft agar.

The SASP modified the composition of PBMC populations with a decrease of T‐helper cells (CD3+, CD4+) and an increase of T‐helper/cytotoxic precursors (CD3+, CD4+, CD8+) and of NKT (CD3+, CD16+, CD56+) cells. The complexity of immune response to cancer is beyond our goals. Still, we would like to provide some hints: (i) the T‐helper cells give signals to cytotoxic lymphocytes within a tumour to establish antitumor immunity; (ii) an increase in CD3+, CD4+ and CD8+ cells were detected in some cancers; (iii) the NKT cells may either eliminate cancer cells or, if overstimulated, they may become ineffective.^46^, ^47^, ^48^ These studies are in line with the changes in PBMC composition we observed following incubation with SASP that impair its anti‐tumour properties.

Generally speaking, we can affirm that the SASP pro‐tumorigenic activity is not autonomous. It should also be remembered that, besides interaction with immune cells, signals coming from cancer cells may modify SASP composition and activity. In a previous paper, we found that the SASP of senescent MSCs loses its anti‐tumour properties if senescent cells are “primed” by allowing them to interact with cancer cells.^49^

## 5 CONCLUSION

We have shown that quiescent stromal cells following SIPS‐produced SASP that, while progressively changing its composition, preserved the capacity to block cancer cell growth by inducing senescence and/or apoptosis only in an autonomous manner. This finding adds further complexity to the way senescent cells act through SASP and demonstrates that SASP activities are cell‐context dependent. This result must be taken into consideration for effective anti‐cancer or anti‐aging therapeutic strategies.

## AUTHOR CONTRIBUTIONS

**Umberto Galderisi**, **Servet Özcan**, **Tiziana Squillaro**: Conceptualization; **Nicola Alessio**, **Giovanni Di Bernardo**, **Servet Özcan**, **Mustafa Burak Acar**, **Domenico Aprile**: Data curation; **Umberto Galderisi**: Funding acquisition; **Tiziana Squillaro**, **Domenico Aprile**, **Mustafa Burak Acar**, **Şerife Ayaz‐Güner**, **Nicola Alessio**: Investigation; **Tiziana Squillaro**, **Mustafa Burak Acar**, **Domenico Aprile**, **Nicola Alessio**: Methodology; **Umberto Galderisi**, **Servet Özcan**, **Gianfranco Peluso**: Supervision; **Nicola Alessio**, **Mustafa Burak Acar**, **Domenico Aprile**, **Şerife Ayaz‐Güner**, **Giovanni Di Bernardo**: Validation; **Nicola Alessio**: Writing (original draft); **Umberto Galderisi**, **Servet Özcan**, **Gianfranco Peluso**: Writing (revised draft).

## FUNDING INFORMATION

The work presented herein was partly supported by grants from Regione Campania Progetto POR “Identificazione, caratterizzazione e significato della tumorigenesi nel colon‐retto: causa, prevenzione e cura—iCURE” CUP B21C17000030007” awarded to U.G. Salary of Assistant Professor for N.A. was obtained from iCURE.

## CONFLICT OF INTEREST

The authors declare no competing interests.

## INFORMED CONSENT

Informed consent was obtained from all subjects involved in the study. The study was approved by Vanvitelli University Ethical Committee (Prot. 0029471/i 14/10/2021), which acts according the Helsinki Declaration.

## Supporting information


**Supplementary file S1.** LC–MS/MS analysis. This file details the LC–MS/MS analyses of peptides from the tryptic digestion of cells' secretomes. In the file are reported the names of genes, which correspond to the proteins identified in the several experimental conditions. The file also reports the Venn analysis performed on the identified factors. C10 stands for quiescent cells at 10D, while IR10, IR30 and IR60 stand for irradiated cells at 10D, 30D and 60D, respectively.

**Supplementary file S2.** Gene Ontology (GO) analysis. The results of the Panther overrepresentation test are reported. The analysis was performed on secretomes obtained 10, 30 and 60 days (10D, 30D, 60D) post‐x‐ray treatment of MSCs and from unirradiated MSCs (CT). Venn diagram evaluation was performed to identify common and specific ontologies among the several experimental conditions.

**Supplementary file S3.** REACTOME analysis. The results of pathway identification by REACTOME test are reported. The analysis was performed on secretomes obtained 10, 30 and 60 days (10D, 30D, 60D) post‐x‐ray treatment of MSCs and from unirradiated MSCs (CT). Venn diagram evaluation was performed to identify common and specific pathways among the several experimental conditions.

**Supplementary file S4.** Network analysis. The results of Minimum IMEx Interactome Networks are reported. The analysis was performed on secretomes obtained 10, 30 and 60 days (10D, 30D, 60D) post‐x‐ray treatment of MSCs and from unirradiated MSCs (CT). Venn diagram evaluation was performed on nodes having a degree higher than 20 and a betweenness higher than 200. The Venn analysis allowed identification of common and specific nodes among the several experimental conditions.

**Supplementary file S5.** Western blot raw data. Uncropped western blot bands reported in Figure 2B.

## Data Availability

Supplementary data to this article can be found online.
