# Supplementary material for: Progression of irradiated mesenchymal stromal cells from early to late senescence: Changes in SASP composition and anti‐tumour properties
Source: Cell Prolif. 2023 Mar 22;56(6):e13401. doi: 10.1111/cpr.13401 (PMC10280137; doi:10.1111/cpr.13401)
Supplement: Supplementary file 1 — Supplementary file S1. LC–MS/MS analysis. This file details the LC–MS/MS analyses of peptides from the tryptic digestion of cells' secretomes. In the file are reported the names of genes, which correspond to the proteins identified in the several experimental conditions. The file also reports the Venn analysis performed on the identified factors. C10 stands for quiescent cells at 10D, while IR10, IR30 and IR60 stand for irradiated cells at 10D, 30D and 60D, respectively. [file CPR-56-e13401-s005.docx]

**Supplementary file 1 – LC-MS/MS analysis**

This file details the LC-MS/MS analyses of peptides from the tryptic digestion of cells’ secretomes. In the file are reported the names of genes, which correspond to the proteins identified in the several experimental conditions. The file also reports the Venn analysis performed on the identified factors. C10 stands for quiescent cells at 10D, while IR10, IR30 and IR60 stand for irradiated cells at 10D, 30D, and 60D, respectively.

List of secreted factors

| **C10** | **IR10** | **IR30** | **IR60** |
| --- | --- | --- | --- |
| P35052 | ABI3BP | ABHD14B | ABHD14B |
| E7EQR4 | ACTB | ABI3BP | ABI3BP |
| P03956 | ACTG1 | ACLY | ACAT1 |
| P02461 | ACTN4 | ACTB | ACTB |
| Q14112 | ACTR2 | ACTG1 | ACTG1 |
| P30101 | AGRN | ACTN1 | ACTN4 |
| A0A7I2YQT6 | AHNAK | ACTN4 | ACY1 |
| P80723 | AHSG | AGRN | ADPRS |
| A0A0G2JIW1 | AIMP1 | AHNAK | AHNAK |
| Q14315 | AK2 | AHSG | AHNAK2 |
| P08572 | AKR1B1 | AK1 | AHSG |
| P07996 | ALB | AK2 | AIMP1 |
| P13497 | ALDOA | AK3 | AK1 |
| H0YA55 | ALDOC | AKR1B1 | AK2 |
| H0YMW4 | ANXA1 | ALDOA | AK3 |
| P05997 | ANXA2 | ALDOC | AKR1A1 |
| P08238 | APEX1 | ALYREF | AKR1B1 |
| P61604 | APP | ANXA1 | ALB |
| B1AHL2 | ARHGDIA | ANXA2 | ALDOA |
| P08123 | ARPC1B | APEX1 | ALDOC |
| P30041 | ARPC4-TTLL3 | APP | ALYREF |
| P63104 | ARPC5 | ARHGDIA | ANPEP |
| J3KPS3 | ATP5F1B | ARPC5 | ANXA1 |
| P01034 | AXL | ATP5F1B | ANXA2 |
| P01023 | B2M | ATP6V1G1 | ANXA5 |
| A0A494C0G5 | BAG3 | AXL | APEX1 |
| O43707 | BASP1 | B2M | ARHGDIA |
| Q09666 | BGN | BAG3 | ATP5PF |
| P63261 | BLVRB | BASP1 | AXL |
| P17936 | C1S | BLVRB | BAG3 |
| D6RE83 | C3 | C1S | BASP1 |
| Q16270 | CALD1 | C3 | BLVRB |
| P51884 | CAP1 | CALD1 | C11orf68 |
| P02452 | CAPZA1 | CAP1 | CALD1 |
| P00558 | CAT | CAPZA1 | CAT |
| P62937 | CAVIN1 | CAST | CAVIN1 |
| P51858 | CAVIN3 | CAT | CAVIN3 |
| P04083 | CCL2 | CAVIN1 | CBR1 |
| O00300 | CCN1 | CAVIN3 | CDV3 |
| O00391 | CD248 | CBR1 | CFL2 |
| P24821 | CDC37 | CCL2 | CIRBP |
| P36955 | CDH11 | CDV3 | CLTA |
| A0A7I2V4I6 | CDV3 | CFL1 | CLTB |
| P07737 | CFL1 | CFL2 | COL1A1 |
| B5MD45 | CFL2 | CIRBP | COL1A2 |
| Q01995 | CLEC11A | CLEC11A | COX5B |
| E9PG40 | CLIC1 | CLSTN1 | COX6B1 |
| Q5SS57 | CLIC4 | CLTA | CRK |
| P21810 | CLSTN1 | CLTB | CRKL |
| P67936 | CLTA | CNDP2 | CRYZ |
| A0A5F9ZHM4 | CLTB | CNN2 | CSRP1 |
| P58215 | CLU | CNN3 | CSRP2 |
| P29401 | CNDP2 | COL1A1 | CST3 |
| P20908 | CNN3 | COL4A2 | CSTB |
| Q08380 | COL12A1 | COL6A1 | CTSD |
| P28799 | COL12A1 | COL6A2 | CTSS |
| P02765 | COL1A1 | COL6A3 | CTTN |
| Q8N2S1-2 | COL1A2 | COTL1 | CYB5R2 |
| A0A087WWU8 | COL3A1 | COX5B | CYCS |
| Q32Q12 | COL4A2 | CRK | DAP |
| Q9NZN4 | COL5A2 | CRKL | DDTL |
| Q16658 | COL6A1 | CRYZ | DLD |
| Q9Y490 | COL6A2 | CSF1 | DNPEP |
| P39060 | COL6A3 | CSRP1 | DPYSL2 |
| P98160 | CORO1B | CSRP2 | DPYSL3 |
| Q04917 | CORO1C | CST3 | DUSP3 |
| Q9HCU0 | CSPG4 | CSTB | DYNLRB2 |
| P08670 | CSRP1 | CTSB | ECI1 |
| P55287 | CSRP2 | CTSD | ECM1 |
| H3BTN5 | CST3 | CTSK | EDF1 |
| Q5H9A7 | CSTB | CTSS | EEF1A1 |
| P09871 | CTSB | CYCS | EFHD2 |
| P23142 | CTSD | DAG1 | EHD2 |
| P61981 | CTSK | DAP | EIF1 |
| P35555 | CTTN | DCN | EIF4EBP1 |
| P16035 | CYCS | DDAH2 | EIF4H |
| P07585 | DAG1 | DDTL | ENO1 |
| P08294 | DAP | DLD | ENSA |

| P30530 | DCN | DNPEP | ERP29 |
| --- | --- | --- | --- |
| Q9NR99 | DCTN2 | DPYSL2 | ETHE1 |
| O94985 | DKK1 | DPYSL3 | EWSR1 |
| Q96C24 | DLD | DSTN | FH |
| E9PQ70 | DPYSL2 | DUSP3 | FKBP1A |
| Q16610 | DPYSL3 | DUT | FKBP2 |
| P31151 | DYNC1LI2 | DYNC1LI2 | FKBP3 |
| A0A0A0MT01 | DYNLRB2 | ECM1 | FLNA |
| Q15149 | ECM1 | EDF1 | FLNC |
| Q5T985 | EEF2 | EEF1A1 | FSCN1 |
| P67809 | EHD2 | EFEMP2 | FUBP1 |
| A0A6Q8PFJ0 | EIF4H | EHD2 | FUS |
| A0A1C7CYX9 | ELAVL1 | EIF4EBP1 | G6PD |
| P22692 | ENO1 | EIF5A | GAPDH |
| Q6EMK4 | ERP29 | EMILIN1 | GLO1 |
| P37802 | EZR | ENO1 | GOT2 |
| O75369 | FBLN1 | ENO2 | GPI |
| P05121 | FBLN1 | ENSA | GPNMB |
| P06733 | FH | ERP29 | GRN |
| P11021 | FHL1 | EWSR1 | GSN |
| P00338 | FKBP1A | EZR | GSR |
| A0A087X0K0 | FKBP3 | FAHD1 | GSTO1 |
| Q15113 | FLNA | FBLN1 | GSTP1 |
| P02751-3 | FLNB | FBLN1 | H1-2 |
| P07093-3 | FLNC | FKBP1A | H1-4 |
| P30086 | FSCN1 | FKBP3 | H2BC15 |
| P26022 | FUBP1 | FLNA | HDDC2 |
| P12814-3 | G3BP1 | FLNB | HEBP1 |
| P18206 | GANAB | FLNC | HEBP2 |
| Q14118 | GAPDH | FN1 | HEXB |
| P11047 | GLO1 | FSCN1 | HINT2 |
| P35579 | GNPTG | FUBP1 | HLA-A |
| A0A087WYF1 | GPC1 | G3BP1 | HLA-C |
| D6RGG3 | GPI | GAPDH | HMGA1 |
| Q12841 | GPNMB | GDF15 | HMGA2 |
| Q9NRN5 | GRN | GLO1 | HMGN2 |
| P31949 | GSN | GLRX | HMGN4 |
| Q02818 | GSR | GNPTG | HNRNPA0 |
| P08253 | GSTP1 | GOT2 | HNRNPA1 |
| A0A087X0S5 | H1-4 | GPI | HNRNPA2B1 |
| P29966 | H1-5 | GPNMB | HSPA1A |
| P60709 | H2BC18 | GRN | HSPA5 |
| Q99497 | HADH | GSN | HSPA8 |
| Q14847 | HBA1 | GSR | HSPB1 |
| A0A7P0Z497 | HDGF | GSTO1 | HSPD1 |
| P24593 | HEXB | GSTP1 | HSPE1 |
| O76061 | HINT1 | H1-2 | IDH1 |
| A0A0U1RRH7 | HMGA1 | H1-4 | IGFBP4 |
| K7ELL7 | HNRNPA1 | H1-5 | IGFBP5 |
| A0A7I2V659 | HNRNPA2B1 | H2BC18 | IGFBP7 |
| P04406 | HNRNPAB | HDDC2 | ITIH2 |
| Q13308 | HNRNPD | HEXB | JPT1 |
| P09603 | HNRNPK | HINT1 | JPT2 |
| A0A499FI48 | HNRNPM | HINT2 | KHSRP |
| Q9Y240 | HSP90AA1 | HLA-A | KRT1 |
| P12110 | HSP90AB1 | HLA-C | KRT2 |
| P09486 | HSP90B1 | HMGA1 | KRT9 |
| E7ENL6 | HSPA1A | HMGN2 | L1CAM |
| A0A0A0MSI0 | HSPA4 | HNRNPA0 | LASP1 |
| P14543 | HSPA5 | HNRNPA2B1 | LDHA |
| A0A5F9UP49 | HSPA8 | HNRNPA3 | LDHB |
| Q8N474 | HSPB1 | HNRNPAB | LEMD2 |
| P07900 | HSPD1 | HNRNPD | LGALS1 |
| H0Y5N9 | HSPE1 | HNRNPK | LGALS3 |
| Q9Y6C2 | HSPG2 | HSPA1A | LMNA |
| E9PK25 | IGFBP3 | HSPA5 | LUM |
| Q9BRK3 | IGFBP4 | HSPA8 | MAN2B1 |
| Q5T7C4 | IGFBP5 | HSPA9 | MAP4 |
| P26038 | IGFBP6 | HSPB1 | MARCKS |
|  | IGFBP7 | HSPE1 | MDH1 |
|  | IL6 | IDH1 | MDH2 |
|  | IQGAP1 | IGFBP3 | MIF |
|  | ITIH2 | IGFBP4 | MMP1 |
|  | JPT1 | IGFBP5 | MMP2 |
|  | KHSRP | IGFBP7 | MMP3 |
|  | KRT1 | IL6 | MSN |
|  | KRT10 | ITIH2 | MTPN |
|  | KRT2 | JPT1 | MYH9 |
|  | KRT9 | KHSRP | NES |
|  | LAMA4 | KRT1 | NME1 |
|  | LAMB2 | KRT10 | NPM1 |

LAMC1 KRT2 PAFAH1B2

LASP1 KTN1 PARK7

LDHA L1CAM PCBP1

LDHB LAMC1 PCBP2 LGALS1 LASP1 PCMT1

LGALS3 LDHA PDIA3

LGALS3BP LDHB PDIA6 LIMA1 LGALS1 PDLIM1

LMNA LGALS3 PDLIM7

LOX LGALS3BP PEBP1

LUM LMNA PFN1

MAN2B1 LOX PGAM1

MANF LUM PGK1 MAP1A MAN2B1 PGLS MAP4 MANF PKM

MARCKS MAP4 PLEC MDH1 MARCKS PLIN3 MDH2 MDH1 POLR2M MIF MDH2 PPIA

MMP1 MIF PPIB MMP2 MMP1 PPP1R14B MMP3 MMP2 PRDX1 MSN MMP3 PRDX2

MVP MSN PRDX3 MXRA5 MXRA8 PRDX5 MXRA8 MYH9 PRDX6

MYH9 NES PRKCSH

MYL12A NID2 PTBP1

NES NME1 PTK7

NID2 NONO PXN

NME1 NPM1 PYM1

NONO NSFL1C QSOX1

NPM1 NUCB1 RAB7A NSFL1C NUCB2 RAD23B NUCB1 P4HB RBM3 NUCB2 PABPC1 RBMX P4HB PAFAH1B2 RCN1 PAFAH1B2 PAMR1 RPL7A PAMR1 PARK7 RPS10

PARK7 PCBP1 RPS12

PCOLCE PCBP2 RPS21 PDIA3 PCMT1 RPS28

PDIA4 PCOLCE RRBP1

PDIA6 PDIA3 S100A10

PDLIM1 PDIA4 S100A11 PDLIM5 PDLIM1 S100A13 PDLIM7 PDLIM2 S100A4 PEBP1 PDLIM5 SDCBP

PFN1 PDLIM7 SEC22B PGAM1 PEBP1 SERPINB1 PGK1 PFN1 SERPINE1

PGLS PGAM1 SERPINE2

PKM PGK1 SERPINF1

PLEC PGLS SH3BGRL3

PLIN3 PKM SH3KBP1

PLOD3 PLEC SNX12

PLS3 PLIN3 SOD2

PPIA PLOD3 SORBS3

PPIB PLPBP SORD

PRDX1 PNPO SRI

PRDX2 PPIA STMN1

PRDX4 PPIB STOM

PRDX5 PPP1R14B STX7 PRDX6 PRDX1 SUMF2 PRKCSH PRDX2 SYTL4

PSMA1 PRDX5 TAF15 PSMA2 PRDX6 TAX1BP3 PSMA5 PRKCSH TBCA PSMA6 PSMA1 TIMP2 PSMA7 PSMA6 TKT

PTK7 PSMA7 TOM1

PTX3 PSME1 TPI1

QSOX1 PTBP1 TPM3

RAD23B PTK7 TPM4

RANBP1 PTX3 TXNDC17

RBMX PYM1 TXNDC5

RCN1 QSOX1 UBE2L3

RDX RAD23B UBE2V1 RPL10A RBM3 UBQLN1

RPL12 RBMX UCHL1

RPS10 RCN1 UGP2

| RPS12 | RDX | VASN |
| --- | --- | --- |
| RPS21 | RPS10 | VAT1 |
| RPS28 | RPS12 | VCL |
| RPSA | RPS21 | VIM |
| RRBP1 | RPS28 | WBP2 |
| S100A11 | RRBP1 | YAP1 |
| S100A13 | S100A11 | YWHAB |
| S100A4 | S100A13 | YWHAZ |
| S100A6 | S100A6 | ZYX |
| SEMA7A | SDCBP |  |
| SEPTIN11 | SERBP1 |  |
| SEPTIN2 | SERPINE1 |  |
| SEPTIN9 | SERPINE2 |  |
| SERPINE1 | SFRP1 |  |
| SERPINE2 | SNX12 |  |
| SERPINH1 | SNX3 |  |
| SFRP1 | SOD2 |  |
| SLC9A3R1 | SORD |  |
| SNX3 | SPARC |  |
| SOD3 | SPOCK1 |  |
| SPARC | SPTAN1 |  |
| SPOCK1 | STC1 |  |
| SPTAN1 | STC2 |  |
| SPTBN1 | STIP1 |  |
| STC1 | STMN1 |  |
| STC2 | STX7 |  |
| STIP1 | SUMF2 |  |
| STMN1 | SYNPO2 |  |
| STX7 | TAF15 |  |
| SUMF2 | TAGLN |  |
| SYNPO2 | TAGLN2 |  |
| TAGLN | TALDO1 |  |
| TAGLN2 | TAX1BP3 |  |
| TAX1BP3 | TBCA |  |
| TBCA | THBS1 |  |
| THBS1 | THBS2 |  |
| THBS2 | TIMP2 |  |
| THY1 | TKT |  |
| TIMP1 | TLN1 |  |
| TIMP2 | TMSB4X |  |
| TKT | TNC |  |
| TLN1 | TPI1 |  |
| TMOD3 | TPM4 |  |
| TNFRSF11B | TUBA1C |  |
| TPM1 | TUBB |  |
| TPM2 | TUBB4B |  |
| TPM3 | TXNDC5 |  |
| TPM4 | TXNRD1 |  |
| TUBA1C | UBA52 |  |
| TUBB4B | UBE2NL |  |
| TXNDC12 | UBQLN1 |  |
| TXNDC5 | UCHL1 |  |
| UCHL1 | VASN |  |
| ULBP2 | VAT1 |  |
| VASN | VCL |  |
| VASP | VCP |  |
| VAT1 | VIM |  |
| VCL | WARS1 |  |
| VCP | WBP2 |  |
| VIM | WDR1 |  |
| WARS1 | YBX1 |  |
| WDR1 | YWHAB |  |
| YBX1 | YWHAG |  |
| YBX3 | YWHAQ |  |
| YWHAG | YWHAZ |  |
| YWHAH | ZYX |  |
| YWHAQ |  |  |
| YWHAZ |  |  |
| ZYX |  |  |

**Venn Diagram**


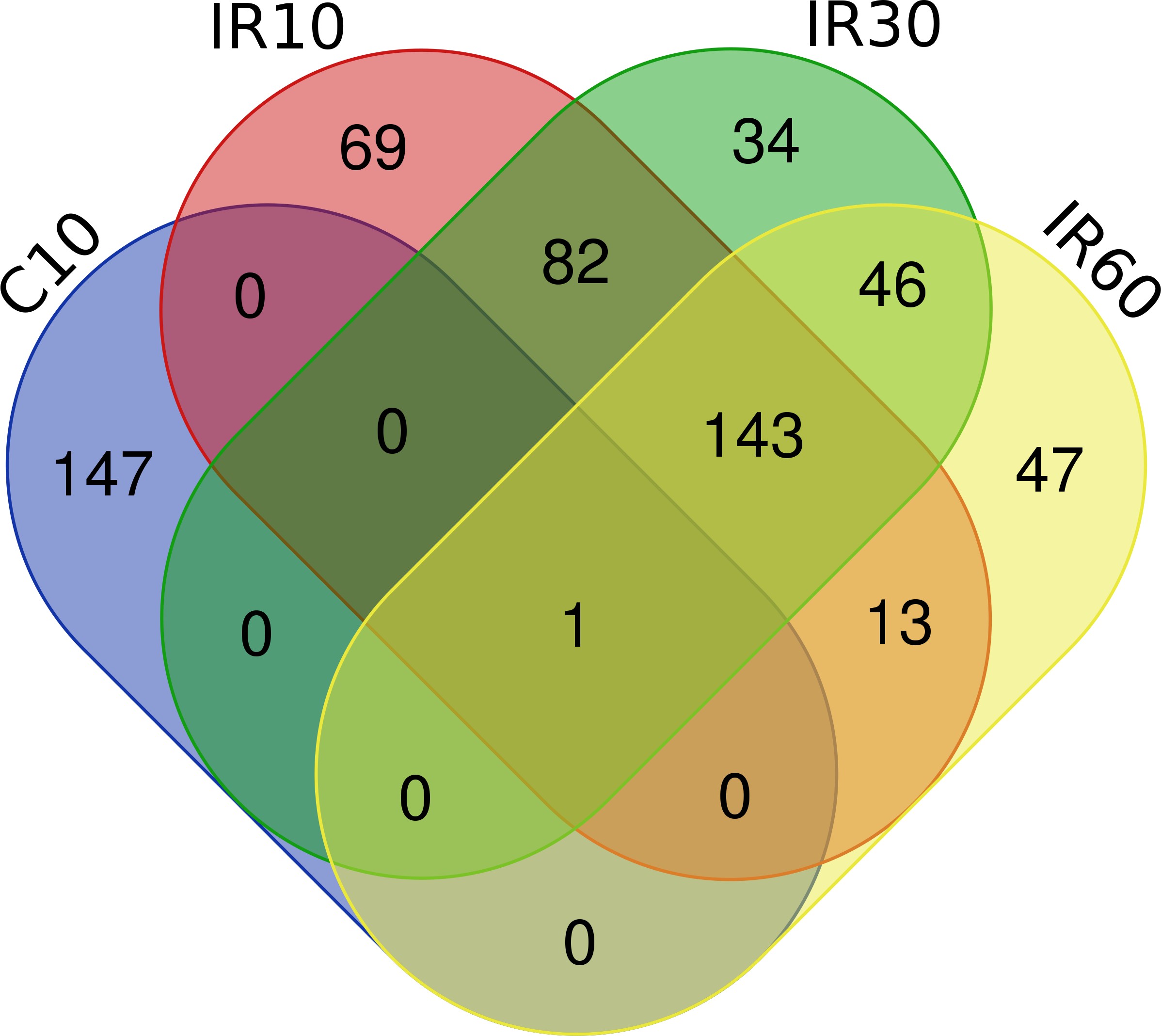


| **Names** | **total** | **elements** |
| --- | --- | --- |
| **C10 IR10 IR30 IR60** | 1 |  |
| **IR10 IR30 IR60** | 143 | ACTN4 |
|  |  | MMP2 |
|  |  | ANXA2 |
|  |  | MAP4 |
|  |  | TKT |
|  |  | GSR |
|  |  | HSPB1 |
|  |  | PRKCSH |
|  |  | JPT1 |
|  |  | LUM |
|  |  | ANXA1 |
|  |  | CYCS |
|  |  | GLO1 |
|  |  | MMP3 |
|  |  | MARCKS |
|  |  | DPYSL2 |
|  |  | AXL |
|  |  | MSN |
|  |  | ERP29 |
|  |  | PRDX6 |
|  |  | GPNMB |
|  |  | TPM4 |
|  |  | BASP1 |
|  |  | SERPINE2 |
|  |  | PDLIM7 |
|  |  | KRT1 |
|  |  | PDLIM1 |
|  |  | HNRNPA2B1 |
|  |  | RPS10 |
|  |  | CLTA |
|  |  | COL1A1 |
|  |  | PDIA3 |
|  |  | ALDOC |
|  |  | UCHL1 |
|  |  | CAVIN3 |
|  |  | TBCA |
|  |  | MDH1 |
|  |  | RAD23B |
|  |  | FSCN1 |
|  |  | LGALS1 |
|  |  | LGALS3 |
|  |  | CFL2 |
|  |  | RPS28 |
|  |  | HSPA5 |
|  |  | TAX1BP3 |
|  |  | RPS12 |
|  |  | MYH9 |
|  |  | FKBP3 |
|  |  | NPM1 |
|  |  | GPI |
|  |  | LDHB |
|  |  | ITIH2 |
|  |  | IGFBP7 |
|  |  | PRDX1 |
|  |  | AKR1B1 |
|  |  | PLEC |
|  |  | MAN2B1 |
|  |  | PAFAH1B2 |
|  |  | FLNC |
|  |  | HSPE1 |
|  |  | CAVIN1 |
|  |  | CST3 |
|  |  | HSPA8 |
|  |  | GRN |
|  |  | KHSRP |
|  |  | GSTP1 |
|  |  | ECM1 |
|  |  | FUBP1 |
|  |  | AHNAK |
|  |  | VASN |
|  |  | RPS21 |
|  |  | PRDX2 |
|  |  | YWHAZ |
|  |  | ACTB |
|  |  | ENO1 |
|  |  | VCL |
|  |  | SERPINE1 |
|  |  | CSRP1 |
|  |  | RBMX |
|  |  | PGAM1 |
|  |  | GSN |
|  |  | ZYX |
|  |  | DLD |
|  |  | PARK7 |
|  |  | CAT |
|  |  | PKM |
|  |  | VIM |
|  |  | ARHGDIA |
|  |  | CDV3 |
|  |  | PTK7 |
|  |  | CSRP2 |
|  |  | LDHA |
|  |  | DAP |
|  |  | QSOX1 |
|  |  | CSTB |
|  |  | PLIN3 |
|  |  | NES |
|  |  | AHSG |
|  |  | EHD2 |
|  |  | STX7 |
|  |  | MIF |
|  |  | PFN1 |
|  |  | TIMP2 |
|  |  | HEXB |
|  |  | BAG3 |
|  |  | ABI3BP |
|  |  | PGLS |
|  |  | CALD1 |
|  |  | HSPA1A |
|  |  | SUMF2 |
|  |  | PPIB |
|  |  | RRBP1 |
|  |  | IGFBP4 |
|  |  | PRDX5 |
|  |  | LMNA |
|  |  | PPIA |
|  |  | TXNDC5 |
|  |  | CLTB |
|  |  | RCN1 |
|  |  | GAPDH |
|  |  | MDH2 |
|  |  | CTSD |
|  |  | APEX1 |
|  |  | VAT1 |
|  |  | HMGA1 |
|  |  | AK2 |
|  |  | ACTG1 |
|  |  | NME1 |
|  |  | ALDOA |
|  |  | FLNA |
|  |  | LASP1 |
|  |  | PEBP1 |
|  |  | FKBP1A |
|  |  | MMP1 |
|  |  | S100A11 |
|  |  | IGFBP5 |
|  |  | DPYSL3 |
|  |  | S100A13 |
|  |  | BLVRB |
|  |  | STMN1 |
|  |  | H1-4 |
|  |  | PGK1 |
|  |  | KRT2 |
| **IR10 IR30** | 82 | YBX1 |
|  |  | TAGLN |
|  |  | G3BP1 |
|  |  | COL4A2 |
|  |  | C1S |
|  |  | TLN1 |

|  | | CLSTN1 |
| --- | --- | --- |
|  |  | HNRNPD |
|  |  | YWHAG |
|  |  | SYNPO2 |
|  |  | CAPZA1 |
|  |  | PCOLCE |
|  |  | PSMA1 |
|  |  | AGRN |
|  |  | MXRA8 |
|  |  | NUCB2 |
|  |  | TAGLN2 |
|  |  | WDR1 |
|  |  | C3 |
|  |  | CFL1 |
|  |  | YWHAQ |
|  |  | PAMR1 |
|  |  | HNRNPAB |
|  |  | THBS1 |
|  |  | NUCB1 |
|  |  | TUBA1C |
|  |  | DYNC1LI2 |
|  |  | PSMA7 |
|  |  | STIP1 |
|  |  | THBS2 |
|  |  | EZR |
|  |  | SPTAN1 |
|  |  | NID2 |
|  |  | CNN3 |
|  |  | SNX3 |
|  |  | NSFL1C |
|  |  | ARPC5 |
|  |  | H2BC18 |
|  |  | MANF |
|  |  | RDX |
|  |  | H1-5 |
|  |  | CTSB |
|  |  | COL6A2 |
|  |  | WARS1 |
|  |  | FBLN1 |
|  |  | STC1 |
|  |  | LOX |
|  |  | CNDP2 |
|  |  | ATP5F1B |
|  |  | LGALS3BP |
|  |  | NONO |
|  |  | COL6A3 |
|  |  | KRT10 |
|  |  | TUBB4B |
|  |  | B2M |
|  |  | CAP1 |
|  |  | HNRNPK |
|  |  | CTSK |
|  |  | P4HB |
|  |  | FLNB |
|  |  | STC2 |
|  |  | COL6A1 |
|  |  | SPARC |
|  |  | IGFBP3 |
|  |  | CLEC11A |
|  |  | PSMA6 |
|  |  | DCN |
|  |  | PTX3 |
|  |  | S100A6 |
|  |  | PDIA4 |
|  |  | DAG1 |
|  |  | PLOD3 |
|  |  | HINT1 |
|  |  | VCP |
|  |  | SFRP1 |
|  |  | CCL2 |
|  |  | LAMC1 |
|  |  | IL6 |
|  |  | APP |
|  |  | PDLIM5 |
|  |  | GNPTG |
|  |  | SPOCK1 |
| **IR10 IR60** | 13 | FH |
|  |  | KRT9 |
|  |  | S100A4 |
|  |  | TPM3 |
|  |  | HSPD1 |
|  |  | AIMP1 |
|  |  | PDIA6 |
|  |  | EIF4H |
|  |  | ALB |
|  |  | DYNLRB2 |
|  |  | COL1A2 |
|  |  | HNRNPA1 |
|  |  | CTTN |
| **IR30 IR60** | 46 | AK1 |
|  |  | L1CAM |
|  |  | HDDC2 |
|  |  | SOD2 |
|  |  | AK3 |
|  |  | DUSP3 |
|  |  | HMGN2 |
|  |  | SDCBP |
|  |  | PPP1R14B |
|  |  | TPI1 |
|  |  | PCMT1 |
|  |  | DNPEP |
|  |  | HINT2 |
|  |  | ENSA |
|  |  | CRYZ |
|  |  | WBP2 |
|  |  | EIF4EBP1 |
|  |  | SORD |
|  |  | COX5B |
|  |  | ALYREF |
|  |  | GOT2 |
|  |  | UBQLN1 |
|  |  | EWSR1 |
|  |  | CTSS |
|  |  | HNRNPA0 |
|  |  | PCBP1 |
|  |  | DDTL |
|  |  | CRK |
|  |  | GSTO1 |
|  |  | HLA-A |
|  |  | CIRBP |
|  |  | IDH1 |
|  |  | SNX12 |
|  |  | PCBP2 |
|  |  | EDF1 |
|  |  | TAF15 |
|  |  | RBM3 |
|  |  | HLA-C |
|  |  | CRKL |
|  |  | PYM1 |
|  |  | ABHD14B |
|  |  | PTBP1 |
|  |  | EEF1A1 |
|  |  | H1-2 |
|  |  | YWHAB |
|  |  | CBR1 |
| **C10** | 147 | P35052 |
|  |  | P03956 |
|  |  | Q14112 |
|  |  | P30101 |
|  |  | A0A7I2YQT6 |
|  |  | P80723 |
|  |  | A0A0G2JIW1 |
|  |  | Q14315 |
|  |  | P08572 |
|  |  | H0YA55 |
|  |  | H0YMW4 |
|  |  | P05997 |
|  |  | P61604 |
|  |  | P08123 |
|  |  | J3KPS3 |
|  |  | P01023 |
|  |  | O43707 |

P17936 Q16270 P02452 P51858 P04083 O00300 P36955 Q01995 E9PG40 P67936 A0A5F9ZHM4 P58215 P29401 P02765 Q16658 P98160 H3BTN5 P23142 P61981 P16035 P08294 P30530 O94985 Q96C24 E9PQ70 Q16610 P31151 Q5T985 P67809 P22692 Q6EMK4 P37802 O75369 P11021 P02751-3 P07093-3 P26022 P12814-3 P18206 D6RGG3 Q12841 Q9NRN5 P31949 Q02818 P08253 P60709 A0A7P0Z497 Q99497 P24593 O76061 Q13308 A0A0A0MSI0 A0A5F9UP49 Q8N474 H0Y5N9 E9PK25 Q9BRK3 E7EQR4 P02461 P07996 P13497 P08238 B1AHL2 P30041 P63104 P01034 A0A494C0G5 Q09666 P63261 D6RE83 P51884 P00558 P62937 O00391 P24821 A0A7I2V4I6 P07737 B5MD45 Q5SS57 P21810 P20908 Q08380 P28799 Q8N2S1-2 A0A087WWU8 Q32Q12 Q9NZN4 P39060 Q9Y490 Q04917 Q9HCU0 P08670 P55287 Q5H9A7 P09871 P35555 P07585 Q9NR99 A0A0A0MT01 Q15149 A0A1C7CYX9 A0A6Q8PFJ0 P05121 P06733 P00338 A0A087X0K0 Q15113 P30086 Q14118 P11047 P35579 A0A087WYF1 A0A087X0S5 P29966 Q14847 A0A0U1RRH7 K7ELL7 P04406 A0A7I2V659 P09603 A0A499FI48 Q9Y240 P12110 P09486 E7ENL6 P14543 P07900 Q9Y6C2 Q5T7C4 P26038

**IR10** 69 PSMA2 COL3A1 RPL10A CSPG4

EEF2 SERPINH1 CLIC4 SEPTIN11 MYL12A IGFBP6 SOD3 RPL12 IQGAP1 TNFRSF11B CLU

HADH HNRNPM ULBP2 SPTBN1 HSP90AB1 PRDX4 HSP90B1

|  | | ARPC1B |
| --- | --- | --- |
|  |  | HSPA4 |
|  |  | ARPC4-TTLL3 |
|  |  | RPSA |
|  |  | SLC9A3R1 |
|  |  | FHL1 |
|  |  | CDC37 |
|  |  | DKK1 |
|  |  | CORO1B |
|  |  | YWHAH |
|  |  | LIMA1 |
|  |  | VASP |
|  |  | GANAB |
|  |  | LAMB2 |
|  |  | CCN1 |
|  |  | CD248 |
|  |  | COL5A2 |
|  |  | TPM2 |
|  |  | THY1 |
|  |  | ELAVL1 |
|  |  | CORO1C |
|  |  | RANBP1 |
|  |  | YBX3 |
|  |  | MAP1A |
|  |  | TPM1 |
|  |  | SEPTIN9 |
|  |  | SEMA7A |
|  |  | HDGF |
|  |  | LAMA4 |
|  |  | MXRA5 |
|  |  | DCTN2 |
|  |  | SEPTIN2 |
|  |  | CDH11 |
|  |  | TXNDC12 |
|  |  | PLS3 |
|  |  | TIMP1 |
|  |  | BGN |
|  |  | HSPG2 |
|  |  | TMOD3 |
|  |  | CLIC1 |
|  |  | PSMA5 |
|  |  | GPC1 |
|  |  | COL12A1 |
|  |  | MVP |
|  |  | HSP90AA1 |
|  |  | ACTR2 |
|  |  | HBA1 |
| **IR30** | 34 | PLPBP |
|  |  | CNN2 |
|  |  | UBE2NL |
|  |  | GDF15 |
|  |  | COTL1 |
|  |  | TNC |
|  |  | PDLIM2 |
|  |  | FAHD1 |
|  |  | HSPA9 |
|  |  | DSTN |
|  |  | CSF1 |
|  |  | TUBB |
|  |  | EMILIN1 |
|  |  | UBA52 |
|  |  | EFEMP2 |
|  |  | TXNRD1 |
|  |  | ATP6V1G1 |
|  |  | DUT |
|  |  | GLRX |
|  |  | FN1 |
|  |  | HNRNPA3 |
|  |  | PABPC1 |
|  |  | ENO2 |
|  |  | TALDO1 |
|  |  | DDAH2 |
|  |  | EIF5A |
|  |  | ACLY |
|  |  | ACTN1 |
|  |  | TMSB4X |
|  |  | SERBP1 |
|  |  | CAST |
|  |  | PNPO |
|  |  | KTN1 |
|  |  | PSME1 |
| **IR60** | 47 | HEBP1 |
|  | | PRDX3 |
|  |  | RPL7A |
|  |  | SERPINF1 |
|  |  | ACY1 |
|  |  | COX6B1 |
|  |  | ACAT1 |
|  |  | EFHD2 |
|  |  | SRI |
|  |  | SERPINB1 |
|  |  | EIF1 |
|  |  | ETHE1 |
|  |  | FUS |
|  |  | G6PD |
|  |  | HMGN4 |
|  |  | LEMD2 |
|  |  | ANPEP |
|  |  | JPT2 |
|  |  | C11orf68 |
|  |  | MTPN |
|  |  | S100A10 |
|  |  | CYB5R2 |
|  |  | SYTL4 |
|  |  | PXN |
|  |  | SH3BGRL3 |
|  |  | STOM |
|  |  | SH3KBP1 |
|  |  | UGP2 |
|  |  | YAP1 |
|  |  | UBE2V1 |
|  |  | SORBS3 |
|  |  | TOM1 |
|  |  | AKR1A1 |
|  |  | SEC22B |
|  |  | AHNAK2 |
|  |  | UBE2L3 |
|  |  | ECI1 |
|  |  | TXNDC17 |
|  |  | H2BC15 |
|  |  | FKBP2 |
|  |  | HEBP2 |
|  |  | POLR2M |
|  |  | ANXA5 |
|  |  | ADPRS |
|  |  | ATP5PF |
|  |  | HMGA2 |
|  |  | RAB7A |
