# Supplementary material for: Progression of irradiated mesenchymal stromal cells from early to late senescence: Changes in SASP composition and anti‐tumour properties
Source: Cell Prolif. 2023 Mar 22;56(6):e13401. doi: 10.1111/cpr.13401 (PMC10280137; doi:10.1111/cpr.13401)
Supplement: Supplementary file 2 — Supplementary file S2. Gene Ontology (GO) analysis. The results of the Panther overrepresentation test are reported. The analysis was performed on secretomes obtained 10, 30 and 60 days (10D, 30D, 60D) post‐x‐ray treatment of MSCs and from unirradiated MSCs (CT). Venn diagram evaluation was performed to identify common and specific ontologies among the several experimental conditions. [file CPR-56-e13401-s004.docx]

**Supplementary file 2 – Gene Ontology (GO) analysis**

The results of the Panther overrepresentation test are reported. The analysis was performed on secretomes obtained 10, 30, and 60 days (10D, 30D, 60D) post-X-ray treatment of MSCs and from unirradiated MSCs (CT). Venn diagram evaluation was performed to identify common and specific ontologies among the several experimental conditions.

Gene Ontology CT

Analysis Type: PANTHER Overrepresentation Test (Released 20210224)

Annotation Version and Release Date: PANTHER version 16.0 Released 2020-12-01

Analyzed List: Client Text Box Input (Homo sapiens)

Reference List: Homo sapiens (all genes in database)

Test Type: FISHER

Correction: FDR

PANTHER GO-Slim Biological Process Homo sapienClient Text B Client Text B Client Text B Client Text B Client Text B Client Text Box Input (FDR)

| cell-substrate junction assembly (GO:0007044) | 5 | 2 .04 | + | 56.81 | 9.96E-04 | 3.15E-02 |
| --- | --- | --- | --- | --- | --- | --- |
| glycolytic process (GO:0006096) | 24 | 5 .17 | + | 29.59 | 1.62E-06 | 8.82E-04 |
| ribonucleoside diphosphate metabolic process (GO:0009185) | 25 | 5 .18 | + | 28.41 | 1.93E-06 | 8.42E-04 |
| purine ribonucleoside diphosphate metabolic process (GO:0009179) | 25 | 5 .18 | + | 28.41 | 1.93E-06 | 7.02E-04 |
| purine nucleoside diphosphate metabolic process (GO:0009135) | 25 | 5 .18 | + | 28.41 | 1.93E-06 | 6.01E-04 |
| ATP generation from ADP (GO:0006757) | 25 | 5 .18 | + | 28.41 | 1.93E-06 | 5.26E-04 |
| ADP metabolic process (GO:0046031) | 25 | 5 .18 | + | 28.41 | 1.93E-06 | 4.68E-04 |
| nucleoside diphosphate phosphorylation (GO:0006165) | 28 | 5 .20 | + | 25.36 | 3.16E-06 | 5.74E-04 |
| nucleotide phosphorylation (GO:0046939) | 28 | 5 .20 | + | 25.36 | 3.16E-06 | 5.30E-04 |
| cellular response to oxidative stress (GO:0034599) | 19 | 3 .13 | + | 22.43 | 4.68E-04 | 1.93E-02 |
| cellular response to chemical stress (GO:0062197) | 20 | 3 .14 | + | 21.31 | 5.35E-04 | 2.12E-02 |
| nucleoside diphosphate metabolic process (GO:0009132) | 34 | 5 .24 | + | 20.89 | 7.41E-06 | 1.08E-03 |
| regulation of angiogenesis (GO:0045765) | 21 | 3 .15 | + | 20.29 | 6.08E-04 | 2.25E-02 |
| 'de novo' protein folding (GO:0006458) | 21 | 3 .15 | + | 20.29 | 6.08E-04 | 2.21E-02 |
| regulation of vasculature development (GO:1901342) | 21 | 3 .15 | + | 20.29 | 6.08E-04 | 2.18E-02 |
| chaperone-mediated protein folding (GO:0061077) | 25 | 3 .18 | + | 17.04 | 9.65E-04 | 3.10E-02 |
| wound healing (GO:0042060) | 34 | 4 .24 | + | 16.71 | 1.41E-04 | 1.06E-02 |
| response to wounding (GO:0009611) | 36 | 4 .25 | + | 15.78 | 1.72E-04 | 1.21E-02 |
| response to oxidative stress (GO:0006979) | 30 | 3 .21 | + | 14.20 | 1.57E-03 | 4.68E-02 |
| carbohydrate catabolic process (GO:0016052) | 50 | 5 .35 | + | 14.20 | 4.09E-05 | 4.47E-03 |
| negative regulation of endopeptidase activity (GO:0010951) | 62 | 6 .44 | + | 13.75 | 8.07E-06 | 1.10E-03 |
| extracellular matrix organization (GO:0030198) | 116 | 11 .82 | + | 13.47 | 1.48E-09 | 3.23E-06 |
| extracellular structure organization (GO:0043062) | 117 | 11 .82 | + | 13.35 | 1.61E-09 | 1.76E-06 |
| negative regulation of peptidase activity (GO:0010466) | 64 | 6 .45 | + | 13.32 | 9.55E-06 | 1.23E-03 |
| protein folding (GO:0006457) | 87 | 8 .61 | + | 13.06 | 3.42E-07 | 2.49E-04 |
| negative regulation of hydrolase activity (GO:0051346) | 78 | 7 .55 | + | 12.75 | 2.21E-06 | 4.82E-04 |
| negative regulation of proteolysis (GO:0045861) | 68 | 6 .48 | + | 12.53 | 1.32E-05 | 1.60E-03 |
| regulation of endopeptidase activity (GO:0052548) | 84 | 6 .59 | + | 10.15 | 4.04E-05 | 4.64E-03 |
| ATP metabolic process (GO:0046034) | 71 | 5 .50 | + | 10.00 | 1.93E-04 | 1.28E-02 |
| regulation of peptidase activity (GO:0052547) | 86 | 6 .61 | + | 9.91 | 4.58E-05 | 4.76E-03 |
| tissue development (GO:0009888) | 157 | 9 1.11 | + | 8.14 | 2.59E-06 | 5.15E-04 |
| negative regulation of catalytic activity (GO:0043086) | 143 | 7 1.01 | + | 6.95 | 9.03E-05 | 7.30E-03 |
| generation of precursor metabolites and energy (GO:0006091) | 126 | 6 .89 | + | 6.76 | 3.35E-04 | 1.62E-02 |
| regulation of proteolysis (GO:0030162) | 128 | 6 .90 | + | 6.66 | 3.63E-04 | 1.65E-02 |
| negative regulation of cellular protein metabolic process (GO:0032269) | 162 | 7 1.14 | + | 6.14 | 1.89E-04 | 1.29E-02 |
| monocarboxylic acid metabolic process (GO:0032787) | 167 | 7 1.18 | + | 5.95 | 2.27E-04 | 1.41E-02 |
| negative regulation of protein metabolic process (GO:0051248) | 168 | 7 1.18 | + | 5.92 | 2.35E-04 | 1.38E-02 |
| negative regulation of molecular function (GO:0044092) | 171 | 7 1.20 | + | 5.81 | 2.60E-04 | 1.46E-02 |
| transmembrane receptor protein tyrosine kinase signaling pathway (GO:0007169) | 212 | 8 1.49 | + | 5.36 | 1.62E-04 | 1.18E-02 |
| ribose phosphate metabolic process (GO:0019693) | 163 | 6 1.15 | + | 5.23 | 1.22E-03 | 3.82E-02 |
| enzyme linked receptor protein signaling pathway (GO:0007167) | 317 | 10 2.23 | + | 4.48 | 1.04E-04 | 8.12E-03 |
| oxidation-reduction process (GO:0055114) | 228 | 7 1.61 | + | 4.36 | 1.35E-03 | 4.09E-02 |
| nucleobase-containing small molecule metabolic process (GO:0055086) | 235 | 7 1.65 | + | 4.23 | 1.60E-03 | 4.70E-02 |
| anatomical structure morphogenesis (GO:0009653) | 492 | 13 3.46 | + | 3.75 | 5.63E-05 | 5.12E-03 |
| multicellular organism development (GO:0007275) | 906 | 17 6.38 | + | 2.67 | 2.53E-04 | 1.45E-02 |
| anatomical structure development (GO:0048856) | 1126 | 21 7.93 | + | 2.65 | 4.90E-05 | 4.64E-03 |
| developmental process (GO:0032502) | 1271 | 21 8.95 | + | 2.35 | 3.44E-04 | 1.63E-02 |
| multicellular organismal process (GO:0032501) | 1385 | 22 9.75 | + | 2.26 | 3.48E-04 | 1.61E-02 |
| macromolecule metabolic process (GO:0043170) | 5051 | 19 35.56 | - | .53 | 9.18E-04 | 2.99E-02 |
| organic substance biosynthetic process (GO:1901576) | 3004 | 8 21.15 | - | .38 | 8.59E-04 | 2.98E-02 |
| biosynthetic process (GO:0009058) | 3011 | 8 21.20 | - | .38 | 8.57E-04 | 3.02E-02 |
| cellular biosynthetic process (GO:0044249) | 2966 | 7 20.88 | - | .34 | 4.70E-04 | 1.90E-02 |
| gene expression (GO:0010467) | 3048 | 7 21.46 | - | .33 | 2.28E-04 | 1.38E-02 |
| cellular nitrogen compound biosynthetic process (GO:0044271) | 2597 | 5 18.28 | - | .27 | 2.26E-04 | 1.45E-02 |
| regulation of RNA metabolic process (GO:0051252) | 2113 | 4 14.88 | - | .27 | 1.30E-03 | 4.01E-02 |
| regulation of cellular biosynthetic process (GO:0031326) | 2134 | 4 15.02 | - | .27 | 9.03E-04 | 3.03E-02 |
| regulation of biosynthetic process (GO:0009889) | 2141 | 4 15.07 | - | .27 | 9.01E-04 | 3.07E-02 |
| regulation of nucleobase-containing compound metabolic process (GO:0019219) | 2161 | 4 15.21 | - | .26 | 9.05E-04 | 2.99E-02 |
| nucleobase-containing compound biosynthetic process (GO:0034654) | 2297 | 4 16.17 | - | .25 | 4.48E-04 | 1.88E-02 |
| heterocycle biosynthetic process (GO:0018130) | 2322 | 4 16.35 | - | .24 | 3.04E-04 | 1.54E-02 |
| aromatic compound biosynthetic process (GO:0019438) | 2323 | 4 16.36 | - | .24 | 3.04E-04 | 1.58E-02 |
| organic cyclic compound biosynthetic process (GO:1901362) | 2350 | 4 16.55 | - | .24 | 3.08E-04 | 1.53E-02 |
| cellular macromolecule biosynthetic process (GO:0034645) | 2580 | 4 18.16 | - | .22 | 6.81E-05 | 5.95E-03 |
| macromolecule biosynthetic process (GO:0009059) | 2590 | 4 18.24 | - | .22 | 6.88E-05 | 5.77E-03 |
| regulation of transcription, DNA-templated (GO:0006355) | 1984 | 3 13.97 | - | .21 | 5.67E-04 | 2.21E-02 |
| regulation of nucleic acid-templated transcription (GO:1903506) | 1984 | 3 13.97 | - | .21 | 5.67E-04 | 2.17E-02 |
| regulation of RNA biosynthetic process (GO:2001141) | 1984 | 3 13.97 | - | .21 | 5.67E-04 | 2.13E-02 |
| RNA metabolic process (GO:0016070) | 2659 | 4 18.72 | - | .21 | 4.74E-05 | 4.70E-03 |
| nucleic acid-templated transcription (GO:0097659) | 2047 | 3 14.41 | - | .21 | 3.91E-04 | 1.74E-02 |
| transcription, DNA-templated (GO:0006351) | 2047 | 3 14.41 | - | .21 | 3.91E-04 | 1.71E-02 |
| RNA biosynthetic process (GO:0032774) | 2054 | 3 14.46 | - | .21 | 3.93E-04 | 1.68E-02 |
| regulation of cellular macromolecule biosynthetic process (GO:2000112) | 2112 | 3 14.87 | - | .20 | 2.70E-04 | 1.47E-02 |
| regulation of macromolecule biosynthetic process (GO:0010556) | 2120 | 3 14.93 | - | .20 | 2.71E-04 | 1.44E-02 |
| nucleic acid metabolic process (GO:0090304) | 2918 | 4 20.54 | - | .19 | 6.43E-06 | 1.00E-03 |

# Gene Ontology 10D

Analysis Type: PANTHER Overrepresentation Test (Released 20210224)

Annotation Version and Release Date: PANTHER version 16.0 Released 2020-12-01

Analyzed List: Client Text Box Input (Homo sapiens)

Reference List: Homo sapiens (all genes in database)

Test Type: FISHER

Correction: FDR

PANTHER GO-Slim Biological Process Homo sapienClient Text B Client Text B Client Text B Client Text B Client Text B Client Text Box Input (FDR)

| positive regulation of protein kinase B signaling (GO:0051897) | 5 | 2 .05 | + | 37.45 | 2.26E-03 | 3.87E-02 |
| --- | --- | --- | --- | --- | --- | --- |
| cellular response to oxidative stress (GO:0034599) | 19 | 6 .20 | + | 29.56 | 1.95E-07 | 5.32E-05 |
| cellular response to chemical stress (GO:0062197) | 20 | 6 .21 | + | 28.08 | 2.51E-07 | 6.09E-05 |
| response to oxidative stress (GO:0006979) | 30 | 8 .32 | + | 24.96 | 5.10E-09 | 1.86E-06 |
| glycolytic process (GO:0006096) | 24 | 6 .26 | + | 23.40 | 6.25E-07 | 1.14E-04 |
| ribonucleoside diphosphate metabolic process (GO:0009185) | 25 | 6 .27 | + | 22.47 | 7.69E-07 | 1.12E-04 |
| purine ribonucleoside diphosphate metabolic process (GO:0009179) | 25 | 6 .27 | + | 22.47 | 7.69E-07 | 1.05E-04 |
| purine nucleoside diphosphate metabolic process (GO:0009135) | 25 | 6 .27 | + | 22.47 | 7.69E-07 | 9.87E-05 |
| ATP generation from ADP (GO:0006757) | 25 | 6 .27 | + | 22.47 | 7.69E-07 | 9.32E-05 |
| ADP metabolic process (GO:0046031) | 25 | 6 .27 | + | 22.47 | 7.69E-07 | 8.83E-05 |
| nucleoside diphosphate phosphorylation (GO:0006165) | 28 | 6 .30 | + | 20.06 | 1.37E-06 | 1.24E-04 |
| nucleotide phosphorylation (GO:0046939) | 28 | 6 .30 | + | 20.06 | 1.37E-06 | 1.19E-04 |
| protein stabilization (GO:0050821) | 19 | 4 .20 | + | 19.71 | 9.18E-05 | 2.99E-03 |
| regulation of protein stability (GO:0031647) | 20 | 4 .21 | + | 18.72 | 1.09E-04 | 3.46E-03 |
| nucleoside diphosphate metabolic process (GO:0009132) | 34 | 6 .36 | + | 16.52 | 3.70E-06 | 2.69E-04 |
| positive regulation of cytoskeleton organization (GO:0051495) | 49 | 7 .52 | + | 13.37 | 2.00E-06 | 1.56E-04 |
| 'de novo' protein folding (GO:0006458) | 21 | 3 .22 | + | 13.37 | 2.00E-03 | 3.52E-02 |
| Arp2/3 complex-mediated actin nucleation (GO:0034314) | 21 | 3 .22 | + | 13.37 | 2.00E-03 | 3.49E-02 |
| actin filament bundle assembly (GO:0051017) | 36 | 5 .38 | + | 13.00 | 6.93E-05 | 2.44E-03 |
| positive regulation of actin filament polymerization (GO:0030838) | 29 | 4 .31 | + | 12.91 | 3.91E-04 | 9.37E-03 |
| positive regulation of supramolecular fiber organization (GO:1902905) | 45 | 6 .48 | + | 12.48 | 1.58E-05 | 8.60E-04 |
| actin filament bundle organization (GO:0061572) | 38 | 5 .41 | + | 12.32 | 8.75E-05 | 2.89E-03 |
| carbohydrate catabolic process (GO:0016052) | 50 | 6 .53 | + | 11.23 | 2.72E-05 | 1.24E-03 |
| muscle structure development (GO:0061061) | 51 | 6 .54 | + | 11.01 | 3.01E-05 | 1.34E-03 |
| protein folding (GO:0006457) | 87 | 10 .93 | + | 10.76 | 8.00E-08 | 2.49E-05 |
| positive regulation of protein polymerization (GO:0032273) | 36 | 4 .38 | + | 10.40 | 8.24E-04 | 1.74E-02 |
| positive regulation of cellular component biogenesis (GO:0044089) | 68 | 7 .73 | + | 9.64 | 1.44E-05 | 8.08E-04 |
| protein depolymerization (GO:0051261) | 40 | 4 .43 | + | 9.36 | 1.18E-03 | 2.31E-02 |
| ATP metabolic process (GO:0046034) | 71 | 7 .76 | + | 9.23 | 1.87E-05 | 9.97E-04 |
| positive regulation of organelle organization (GO:0010638) | 82 | 8 .88 | + | 9.13 | 5.05E-06 | 3.34E-04 |
| regulation of actin filament organization (GO:0110053) | 82 | 8 .88 | + | 9.13 | 5.05E-06 | 3.24E-04 |
| positive regulation of protein-containing complex assembly (GO:0031334) | 41 | 4 .44 | + | 9.13 | 1.29E-03 | 2.49E-02 |
| regulation of actin filament polymerization (GO:0030833) | 72 | 7 .77 | + | 9.10 | 2.04E-05 | 1.03E-03 |
| actin polymerization or depolymerization (GO:0008154) | 84 | 8 .90 | + | 8.92 | 5.96E-06 | 3.71E-04 |
| regulation of actin filament length (GO:0030832) | 74 | 7 .79 | + | 8.86 | 2.40E-05 | 1.19E-03 |
| regulation of actin polymerization or depolymerization (GO:0008064) | 74 | 7 .79 | + | 8.86 | 2.40E-05 | 1.16E-03 |
| developmental growth (GO:0048589) | 44 | 4 .47 | + | 8.51 | 1.64E-03 | 3.04E-02 |
| actin filament polymerization (GO:0030041) | 78 | 7 .83 | + | 8.40 | 3.29E-05 | 1.41E-03 |
| regulation of protein polymerization (GO:0032271) | 79 | 7 .84 | + | 8.29 | 3.55E-05 | 1.41E-03 |
| actin filament organization (GO:0007015) | 184 | 16 1.97 | + | 8.14 | 4.28E-10 | 2.33E-07 |
| extracellular matrix organization (GO:0030198) | 116 | 10 1.24 | + | 8.07 | 9.39E-07 | 9.76E-05 |
| actin cytoskeleton organization (GO:0030036) | 268 | 23 2.86 | + | 8.03 | 6.99E-14 | 1.53E-10 |
| regulation of actin cytoskeleton organization (GO:0032956) | 105 | 9 1.12 | + | 8.02 | 3.47E-06 | 2.61E-04 |
| extracellular structure organization (GO:0043062) | 117 | 10 1.25 | + | 8.00 | 1.01E-06 | 1.00E-04 |
| regulation of supramolecular fiber organization (GO:1902903) | 94 | 8 1.00 | + | 7.97 | 1.28E-05 | 7.36E-04 |
| regulation of actin filament-based process (GO:0032970) | 106 | 9 1.13 | + | 7.95 | 3.73E-06 | 2.62E-04 |
| response to inorganic substance (GO:0010035) | 59 | 5 .63 | + | 7.93 | 5.79E-04 | 1.29E-02 |
| actin filament-based process (GO:0030029) | 280 | 23 2.99 | + | 7.69 | 1.64E-13 | 1.79E-10 |
| cellular protein complex disassembly (GO:0043624) | 50 | 4 .53 | + | 7.49 | 2.54E-03 | 4.30E-02 |
| generation of precursor metabolites and energy (GO:0006091) | 126 | 10 1.35 | + | 7.43 | 1.89E-06 | 1.53E-04 |
| positive regulation of cellular component organization (GO:0051130) | 139 | 11 1.48 | + | 7.41 | 5.90E-07 | 1.17E-04 |
| regulation of cellular component size (GO:0032535) | 108 | 8 1.15 | + | 6.93 | 3.27E-05 | 1.43E-03 |
| supramolecular fiber organization (GO:0097435) | 274 | 20 2.93 | + | 6.83 | 4.94E-11 | 3.59E-08 |
| regulation of protein-containing complex assembly (GO:0043254) | 96 | 7 1.03 | + | 6.83 | 1.12E-04 | 3.50E-03 |
| regulation of cytoskeleton organization (GO:0051493) | 151 | 11 1.61 | + | 6.82 | 1.27E-06 | 1.20E-04 |
| tissue development (GO:0009888) | 157 | 11 1.68 | + | 6.56 | 1.81E-06 | 1.52E-04 |
| protein polymerization (GO:0051258) | 105 | 7 1.12 | + | 6.24 | 1.90E-04 | 5.31E-03 |
| regulation of anatomical structure size (GO:0090066) | 123 | 8 1.31 | + | 6.09 | 7.79E-05 | 2.70E-03 |
| negative regulation of hydrolase activity (GO:0051346) | 78 | 5 .83 | + | 6.00 | 1.88E-03 | 3.35E-02 |
| regulation of cellular component biogenesis (GO:0044087) | 161 | 9 1.72 | + | 5.23 | 8.47E-05 | 2.89E-03 |
| ribose phosphate metabolic process (GO:0019693) | 163 | 9 1.74 | + | 5.17 | 9.26E-05 | 2.97E-03 |
| regulation of organelle organization (GO:0033043) | 275 | 15 2.94 | + | 5.11 | 5.02E-07 | 1.10E-04 |
| purine ribonucleotide metabolic process (GO:0009150) | 149 | 8 1.59 | + | 5.03 | 2.72E-04 | 6.91E-03 |
| ribonucleotide metabolic process (GO:0009259) | 155 | 8 1.66 | + | 4.83 | 3.50E-04 | 8.50E-03 |
| purine nucleotide metabolic process (GO:0006163) | 166 | 8 1.77 | + | 4.51 | 5.41E-04 | 1.24E-02 |
| monocarboxylic acid metabolic process (GO:0032787) | 167 | 8 1.78 | + | 4.48 | 5.62E-04 | 1.28E-02 |
| nucleotide metabolic process (GO:0009117) | 196 | 9 2.09 | + | 4.30 | 3.45E-04 | 8.46E-03 |
| cell migration (GO:0016477) | 263 | 12 2.81 | + | 4.27 | 3.86E-05 | 1.50E-03 |
| purine-containing compound metabolic process (GO:0072521) | 178 | 8 1.90 | + | 4.21 | 8.38E-04 | 1.76E-02 |
| nucleoside phosphate metabolic process (GO:0006753) | 202 | 9 2.16 | + | 4.17 | 4.26E-04 | 9.99E-03 |
| regulation of cellular component organization (GO:0051128) | 410 | 18 4.38 | + | 4.11 | 7.52E-07 | 1.26E-04 |
| oxidation-reduction process (GO:0055114) | 228 | 10 2.44 | + | 4.11 | 2.32E-04 | 6.33E-03 |
| cytoskeleton organization (GO:0007010) | 649 | 28 6.93 | + | 4.04 | 7.50E-10 | 3.27E-07 |
| localization of cell (GO:0051674) | 304 | 13 3.25 | + | 4.00 | 3.50E-05 | 1.44E-03 |
| cell motility (GO:0048870) | 304 | 13 3.25 | + | 4.00 | 3.50E-05 | 1.42E-03 |
| nucleobase-containing small molecule metabolic process (GO:0055086) | 235 | 10 2.51 | + | 3.98 | 2.93E-04 | 7.34E-03 |
| locomotion (GO:0040011) | 384 | 15 4.10 | + | 3.66 | 2.42E-05 | 1.15E-03 |
| cellular homeostasis (GO:0019725) | 234 | 9 2.50 | + | 3.60 | 1.16E-03 | 2.29E-02 |
| transmembrane receptor protein tyrosine kinase signaling pathway (GO:0007169) | 212 | 8 2.26 | + | 3.53 | 2.43E-03 | 4.14E-02 |
| protein-containing complex subunit organization (GO:0043933) | 516 | 17 5.51 | + | 3.08 | 5.58E-05 | 1.99E-03 |
| animal organ development (GO:0048513) | 385 | 12 4.11 | + | 2.92 | 1.11E-03 | 2.20E-02 |
| homeostatic process (GO:0042592) | 360 | 11 3.85 | + | 2.86 | 2.06E-03 | 3.57E-02 |
| cellular response to stress (GO:0033554) | 500 | 15 5.34 | + | 2.81 | 4.04E-04 | 9.58E-03 |
| cellular protein-containing complex assembly (GO:0034622) | 407 | 12 4.35 | + | 2.76 | 1.75E-03 | 3.16E-02 |
| regulation of biological quality (GO:0065008) | 890 | 26 9.51 | + | 2.73 | 4.73E-06 | 3.22E-04 |
| anatomical structure morphogenesis (GO:0009653) | 492 | 14 5.26 | + | 2.66 | 1.03E-03 | 2.12E-02 |
| protein-containing complex assembly (GO:0065003) | 433 | 12 4.63 | + | 2.59 | 2.87E-03 | 4.81E-02 |
| cellular component assembly (GO:0022607) | 909 | 25 9.71 | + | 2.57 | 1.97E-05 | 1.02E-03 |
| catabolic process (GO:0009056) | 921 | 25 9.84 | + | 2.54 | 2.44E-05 | 1.13E-03 |
| movement of cell or subcellular component (GO:0006928) | 595 | 16 6.36 | + | 2.52 | 8.22E-04 | 1.76E-02 |
| cellular component biogenesis (GO:0044085) | 1040 | 27 11.11 | + | 2.43 | 3.30E-05 | 1.39E-03 |
| system development (GO:0048731) | 804 | 19 8.59 | + | 2.21 | 1.36E-03 | 2.58E-02 |
| multicellular organism development (GO:0007275) | 906 | 21 9.68 | + | 2.17 | 1.34E-03 | 2.57E-02 |
| response to chemical (GO:0042221) | 822 | 19 8.78 | + | 2.16 | 1.68E-03 | 3.09E-02 |
| anatomical structure development (GO:0048856) | 1126 | 26 12.03 | + | 2.16 | 2.68E-04 | 6.89E-03 |
| response to stress (GO:0006950) | 967 | 22 10.33 | + | 2.13 | 1.08E-03 | 2.19E-02 |

| cellular component organization (GO:0016043) | 2624 | 55 28.03 | + | 1.96 | 8.26E-07 | 9.01E-05 |
| --- | --- | --- | --- | --- | --- | --- |
| cellular component organization or biogenesis (GO:0071840) | 2750 | 57 29.38 | + | 1.94 | 7.58E-07 | 1.18E-04 |
| developmental process (GO:0032502) | 1271 | 26 13.58 | + | 1.91 | 1.72E-03 | 3.13E-02 |
| organelle organization (GO:0006996) | 1979 | 39 21.14 | + | 1.84 | 2.05E-04 | 5.66E-03 |
| biological_process (GO:0008150) | 10802 | 146 115.39 | + | 1.27 | 4.20E-05 | 1.55E-03 |
| cellular process (GO:0009987) | 9951 | 134 106.30 | + | 1.26 | 2.35E-04 | 6.32E-03 |
| Unclassified (UNCLASSIFIED) | 9793 | 74 104.61 | - | .71 | 4.20E-05 | 1.58E-03 |
| organic substance biosynthetic process (GO:1901576) | 3004 | 16 32.09 | - | .50 | 1.39E-03 | 2.61E-02 |
| biosynthetic process (GO:0009058) | 3011 | 16 32.16 | - | .50 | 1.40E-03 | 2.60E-02 |
| cellular biosynthetic process (GO:0044249) | 2966 | 15 31.68 | - | .47 | 6.71E-04 | 1.48E-02 |
| cellular nitrogen compound biosynthetic process (GO:0044271) | 2597 | 13 27.74 | - | .47 | 1.96E-03 | 3.48E-02 |
| RNA metabolic process (GO:0016070) | 2659 | 13 28.40 | - | .46 | 1.10E-03 | 2.19E-02 |
| regulation of gene expression (GO:0010468) | 2410 | 11 25.74 | - | .43 | 9.75E-04 | 2.03E-02 |
| nucleic acid metabolic process (GO:0090304) | 2918 | 13 31.17 | - | .42 | 1.85E-04 | 5.24E-03 |
| nucleobase-containing compound biosynthetic process (GO:0034654) | 2297 | 10 24.54 | - | .41 | 1.07E-03 | 2.18E-02 |
| heterocycle biosynthetic process (GO:0018130) | 2322 | 10 24.80 | - | .40 | 7.77E-04 | 1.69E-02 |
| aromatic compound biosynthetic process (GO:0019438) | 2323 | 10 24.81 | - | .40 | 7.77E-04 | 1.68E-02 |
| organic cyclic compound biosynthetic process (GO:1901362) | 2350 | 10 25.10 | - | .40 | 5.71E-04 | 1.28E-02 |
| cellular macromolecule biosynthetic process (GO:0034645) | 2580 | 10 27.56 | - | .36 | 1.20E-04 | 3.55E-03 |
| macromolecule biosynthetic process (GO:0009059) | 2590 | 10 27.67 | - | .36 | 8.58E-05 | 2.88E-03 |
| regulation of RNA metabolic process (GO:0051252) | 2113 | 8 22.57 | - | .35 | 4.54E-04 | 1.05E-02 |
| regulation of nucleobase-containing compound metabolic process (GO:0019219) | 2161 | 8 23.08 | - | .35 | 3.31E-04 | 8.21E-03 |
| nucleic acid-templated transcription (GO:0097659) | 2047 | 7 21.87 | - | .32 | 2.38E-04 | 6.34E-03 |
| transcription, DNA-templated (GO:0006351) | 2047 | 7 21.87 | - | .32 | 2.38E-04 | 6.26E-03 |
| RNA biosynthetic process (GO:0032774) | 2054 | 7 21.94 | - | .32 | 2.39E-04 | 6.20E-03 |
| regulation of cellular biosynthetic process (GO:0031326) | 2134 | 7 22.80 | - | .31 | 1.23E-04 | 3.57E-03 |
| regulation of biosynthetic process (GO:0009889) | 2141 | 7 22.87 | - | .31 | 1.23E-04 | 3.54E-03 |
| regulation of transcription, DNA-templated (GO:0006355) | 1984 | 6 21.19 | - | .28 | 1.18E-04 | 3.61E-03 |
| regulation of nucleic acid-templated transcription (GO:1903506) | 1984 | 6 21.19 | - | .28 | 1.18E-04 | 3.56E-03 |
| regulation of RNA biosynthetic process (GO:2001141) | 1984 | 6 21.19 | - | .28 | 1.18E-04 | 3.51E-03 |
| regulation of cellular macromolecule biosynthetic process (GO:2000112) | 2112 | 6 22.56 | - | .27 | 4.18E-05 | 1.60E-03 |
| regulation of macromolecule biosynthetic process (GO:0010556) | 2120 | 6 22.65 | - | .26 | 4.23E-05 | 1.54E-03 |
| regulation of transcription by RNA polymerase II (GO:0006357) | 1591 | 2 17.00 | - | .12 | 8.88E-06 | 5.23E-04 |
| transcription by RNA polymerase II (GO:0006366) | 1635 | 2 17.47 | - | .11 | 6.12E-06 | 3.71E-04 |

Gene Ontology 30D

Analysis Type: PANTHER Overrepresentation Test (Released 20210224)

Annotation Version and Release Date: PANTHER version 16.0 Released 2020-12-01

Analyzed List: Client Text Box Input (Homo sapiens)

Reference List: Homo sapiens (all genes in database)

Test Type: FISHER

Correction: FDR

PANTHER GO-Slim Biological Process Homo sapienClient Text B Client Text B Client Text B Client Text B Client Text B Client Text Box Input (FDR)

| glycolytic process (GO:0006096) | 24 | 7 .24 | + | 28.74 | 2.00E-08 | 2.18E-05 |
| --- | --- | --- | --- | --- | --- | --- |
| ribonucleoside diphosphate metabolic process (GO:0009185) | 25 | 7 .25 | + | 27.59 | 2.54E-08 | 1.85E-05 |
| purine ribonucleoside diphosphate metabolic process (GO:0009179) | 25 | 7 .25 | + | 27.59 | 2.54E-08 | 1.39E-05 |
| purine nucleoside diphosphate metabolic process (GO:0009135) | 25 | 7 .25 | + | 27.59 | 2.54E-08 | 1.11E-05 |
| ATP generation from ADP (GO:0006757) | 25 | 7 .25 | + | 27.59 | 2.54E-08 | 9.24E-06 |
| ADP metabolic process (GO:0046031) | 25 | 7 .25 | + | 27.59 | 2.54E-08 | 7.92E-06 |
| cellular response to oxidative stress (GO:0034599) | 19 | 5 .19 | + | 25.93 | 3.55E-06 | 3.23E-04 |
| nucleoside diphosphate phosphorylation (GO:0006165) | 28 | 7 .28 | + | 24.64 | 4.95E-08 | 1.35E-05 |
| cellular response to chemical stress (GO:0062197) | 20 | 5 .20 | + | 24.64 | 4.40E-06 | 3.84E-04 |
| nucleotide phosphorylation (GO:0046939) | 28 | 7 .28 | + | 24.64 | 4.95E-08 | 1.20E-05 |
| nucleoside diphosphate metabolic process (GO:0009132) | 34 | 8 .35 | + | 23.19 | 7.99E-09 | 1.74E-05 |
| NADP metabolic process (GO:0006739) | 13 | 3 .13 | + | 22.74 | 5.08E-04 | 2.36E-02 |
| negative regulation of chromatin silencing (GO:0031936) | 13 | 3 .13 | + | 22.74 | 5.08E-04 | 2.31E-02 |
| response to oxidative stress (GO:0006979) | 30 | 6 .30 | + | 19.71 | 1.45E-06 | 1.86E-04 |
| regulation of chromatin silencing (GO:0031935) | 15 | 3 .15 | + | 19.71 | 7.30E-04 | 2.95E-02 |
| negative regulation of DNA recombination (GO:0045910) | 19 | 3 .19 | + | 15.56 | 1.34E-03 | 4.63E-02 |
| regulation of gene silencing (GO:0060968) | 20 | 3 .20 | + | 14.78 | 1.53E-03 | 4.97E-02 |
| carbohydrate catabolic process (GO:0016052) | 50 | 7 .51 | + | 13.80 | 1.61E-06 | 1.85E-04 |
| ATP metabolic process (GO:0046034) | 71 | 9 .72 | + | 12.49 | 1.10E-07 | 2.40E-05 |
| protein depolymerization (GO:0051261) | 40 | 4 .41 | + | 9.85 | 9.81E-04 | 3.63E-02 |
| muscle structure development (GO:0061061) | 51 | 5 .52 | + | 9.66 | 2.46E-04 | 1.28E-02 |
| generation of precursor metabolites and energy (GO:0006091) | 126 | 11 1.28 | + | 8.60 | 1.42E-07 | 2.81E-05 |
| regulation of mRNA splicing, via spliceosome (GO:0048024) | 65 | 5 .66 | + | 7.58 | 6.95E-04 | 3.03E-02 |
| regulation of endopeptidase activity (GO:0052548) | 84 | 6 .85 | + | 7.04 | 2.95E-04 | 1.50E-02 |
| protein-containing complex disassembly (GO:0032984) | 71 | 5 .72 | + | 6.94 | 1.01E-03 | 3.68E-02 |
| regulation of peptidase activity (GO:0052547) | 86 | 6 .87 | + | 6.87 | 3.33E-04 | 1.61E-02 |
| regulation of RNA splicing (GO:0043484) | 88 | 6 .89 | + | 6.72 | 3.74E-04 | 1.77E-02 |
| ribose phosphate metabolic process (GO:0019693) | 163 | 11 1.65 | + | 6.65 | 1.56E-06 | 1.89E-04 |
| purine ribonucleotide metabolic process (GO:0009150) | 149 | 10 1.51 | + | 6.61 | 4.91E-06 | 4.12E-04 |
| regulation of mRNA processing (GO:0050684) | 75 | 5 .76 | + | 6.57 | 1.27E-03 | 4.48E-02 |
| purine nucleotide metabolic process (GO:0006163) | 166 | 11 1.68 | + | 6.53 | 1.84E-06 | 1.83E-04 |
| ribonucleotide metabolic process (GO:0009259) | 155 | 10 1.57 | + | 6.36 | 6.82E-06 | 5.51E-04 |
| negative regulation of hydrolase activity (GO:0051346) | 78 | 5 .79 | + | 6.32 | 1.50E-03 | 5.04E-02 |
| purine-containing compound metabolic process (GO:0072521) | 178 | 11 1.81 | + | 6.09 | 3.48E-06 | 3.30E-04 |
| nucleotide metabolic process (GO:0009117) | 196 | 12 1.99 | + | 6.03 | 1.36E-06 | 1.98E-04 |
| nucleoside phosphate metabolic process (GO:0006753) | 202 | 12 2.05 | + | 5.85 | 1.84E-06 | 1.91E-04 |
| oxidation-reduction process (GO:0055114) | 228 | 13 2.31 | + | 5.62 | 1.05E-06 | 1.76E-04 |
| nucleobase-containing small molecule metabolic process (GO:0055086) | 235 | 13 2.38 | + | 5.45 | 1.45E-06 | 1.97E-04 |
| monocarboxylic acid metabolic process (GO:0032787) | 167 | 9 1.69 | + | 5.31 | 7.49E-05 | 4.95E-03 |
| negative regulation of cellular component organization (GO:0051129) | 114 | 6 1.16 | + | 5.19 | 1.35E-03 | 4.61E-02 |
| actin cytoskeleton organization (GO:0030036) | 268 | 14 2.72 | + | 5.15 | 1.08E-06 | 1.68E-04 |
| carbohydrate metabolic process (GO:0005975) | 140 | 7 1.42 | + | 4.93 | 7.26E-04 | 2.99E-02 |
| actin filament-based process (GO:0030029) | 280 | 14 2.84 | + | 4.93 | 1.77E-06 | 1.93E-04 |
| actin filament organization (GO:0007015) | 184 | 9 1.87 | + | 4.82 | 1.51E-04 | 8.68E-03 |
| regulation of cytoskeleton organization (GO:0051493) | 151 | 7 1.53 | + | 4.57 | 1.11E-03 | 3.96E-02 |
| regulation of organelle organization (GO:0033043) | 275 | 12 2.79 | + | 4.30 | 3.56E-05 | 2.59E-03 |
| supramolecular fiber organization (GO:0097435) | 274 | 11 2.78 | + | 3.96 | 1.53E-04 | 8.56E-03 |
| carboxylic acid metabolic process (GO:0019752) | 338 | 13 3.43 | + | 3.79 | 5.89E-05 | 4.15E-03 |
| oxoacid metabolic process (GO:0043436) | 357 | 13 3.62 | + | 3.59 | 1.00E-04 | 6.43E-03 |
| organophosphate metabolic process (GO:0019637) | 388 | 14 3.94 | + | 3.56 | 5.95E-05 | 4.06E-03 |
| organic acid metabolic process (GO:0006082) | 375 | 13 3.81 | + | 3.42 | 1.60E-04 | 8.53E-03 |
| regulation of cellular component organization (GO:0051128) | 410 | 14 4.16 | + | 3.36 | 1.05E-04 | 6.53E-03 |
| catabolic process (GO:0009056) | 921 | 28 9.35 | + | 3.00 | 3.26E-07 | 5.93E-05 |
| carbohydrate derivative metabolic process (GO:1901135) | 429 | 13 4.35 | + | 2.99 | 5.58E-04 | 2.48E-02 |
| protein-containing complex subunit organization (GO:0043933) | 516 | 15 5.24 | + | 2.86 | 3.25E-04 | 1.61E-02 |
| cytoskeleton organization (GO:0007010) | 649 | 18 6.59 | + | 2.73 | 1.45E-04 | 8.58E-03 |
| small molecule metabolic process (GO:0044281) | 654 | 18 6.64 | + | 2.71 | 1.59E-04 | 8.70E-03 |
| organonitrogen compound catabolic process (GO:1901565) | 480 | 13 4.87 | + | 2.67 | 1.51E-03 | 4.98E-02 |
| organic substance catabolic process (GO:1901575) | 753 | 20 7.64 | + | 2.62 | 1.09E-04 | 6.63E-03 |
| cellular catabolic process (GO:0044248) | 816 | 19 8.28 | + | 2.29 | 9.77E-04 | 3.68E-02 |
| cellular macromolecule biosynthetic process (GO:0034645) | 2580 | 11 26.18 | - | .42 | 6.95E-04 | 2.97E-02 |
| macromolecule biosynthetic process (GO:0009059) | 2590 | 11 26.28 | - | .42 | 6.96E-04 | 2.92E-02 |
| nucleic acid-templated transcription (GO:0097659) | 2047 | 8 20.77 | - | .39 | 1.54E-03 | 4.93E-02 |
| transcription, DNA-templated (GO:0006351) | 2047 | 8 20.77 | - | .39 | 1.54E-03 | 4.86E-02 |
| RNA biosynthetic process (GO:0032774) | 2054 | 8 20.84 | - | .38 | 1.54E-03 | 4.80E-02 |
| regulation of transcription, DNA-templated (GO:0006355) | 1984 | 7 20.13 | - | .35 | 8.46E-04 | 3.36E-02 |
| regulation of nucleic acid-templated transcription (GO:1903506) | 1984 | 7 20.13 | - | .35 | 8.46E-04 | 3.30E-02 |
| regulation of RNA biosynthetic process (GO:2001141) | 1984 | 7 20.13 | - | .35 | 8.46E-04 | 3.24E-02 |
| regulation of transcription by RNA polymerase II (GO:0006357) | 1591 | 2 16.15 | - | .12 | 1.85E-05 | 1.39E-03 |
| transcription by RNA polymerase II (GO:0006366) | 1635 | 2 16.59 | - | .12 | 1.26E-05 | 9.85E-04 |

# Gene Ontology 60D

Analysis Type: PANTHER Overrepresentation Test (Released 20210224)

Annotation Version and Release Date: PANTHER version 16.0 Released 2020-12-01

Analyzed List: Client Text Box Input (Homo sapiens)

Reference List: Homo sapiens (all genes in database)

Test Type: FISHER

Correction: FDR

PANTHER GO-Slim Biological Process Homo sapienClient Text B Client Text B Client Text B Client Text B Client Text B Client Text Box Input (FDR

| cellular response to oxidative stress (GO:0034599) | 19 | 6 .16 | + | 36.95 | 5.27E-08 | 8.84E-06 |
| --- | --- | --- | --- | --- | --- | --- |
| cellular response to chemical stress (GO:0062197) | 20 | 6 .17 | + | 35.11 | 6.80E-08 | 1.06E-05 |
| glycolytic process (GO:0006096) | 24 | 7 .21 | + | 34.13 | 6.16E-09 | 4.48E-06 |
| ribonucleoside diphosphate metabolic process (GO:0009185) | 25 | 7 .21 | + | 32.76 | 7.83E-09 | 4.27E-06 |
| purine ribonucleoside diphosphate metabolic process (GO:0009179) | 25 | 7 .21 | + | 32.76 | 7.83E-09 | 3.42E-06 |
| purine nucleoside diphosphate metabolic process (GO:0009135) | 25 | 7 .21 | + | 32.76 | 7.83E-09 | 2.85E-06 |
| ATP generation from ADP (GO:0006757) | 25 | 7 .21 | + | 32.76 | 7.83E-09 | 2.44E-06 |
| ADP metabolic process (GO:0046031) | 25 | 7 .21 | + | 32.76 | 7.83E-09 | 2.14E-06 |
| nucleoside diphosphate phosphorylation (GO:0006165) | 28 | 7 .24 | + | 29.25 | 1.53E-08 | 3.71E-06 |
| nucleotide phosphorylation (GO:0046939) | 28 | 7 .24 | + | 29.25 | 1.53E-08 | 3.34E-06 |
| nucleoside diphosphate metabolic process (GO:0009132) | 34 | 8 .29 | + | 27.53 | 2.09E-09 | 4.56E-06 |
| response to oxidative stress (GO:0006979) | 30 | 7 .26 | + | 27.30 | 2.31E-08 | 4.59E-06 |
| NADP metabolic process (GO:0006739) | 13 | 3 .11 | + | 27.00 | 3.09E-04 | 1.60E-02 |
| carbohydrate catabolic process (GO:0016052) | 50 | 7 .43 | + | 16.38 | 5.15E-07 | 7.02E-05 |
| ATP metabolic process (GO:0046034) | 71 | 9 .61 | + | 14.83 | 2.54E-08 | 4.61E-06 |
| protein depolymerization (GO:0051261) | 40 | 4 .34 | + | 11.70 | 5.19E-04 | 2.52E-02 |
| generation of precursor metabolites and energy (GO:0006091) | 126 | 12 1.08 | + | 11.14 | 2.27E-09 | 2.47E-06 |
| negative regulation of endopeptidase activity (GO:0010951) | 62 | 5 .53 | + | 9.44 | 2.61E-04 | 1.46E-02 |
| cellular protein complex disassembly (GO:0043624) | 50 | 4 .43 | + | 9.36 | 1.13E-03 | 4.66E-02 |
| negative regulation of peptidase activity (GO:0010466) | 64 | 5 .55 | + | 9.14 | 2.99E-04 | 1.59E-02 |
| negative regulation of hydrolase activity (GO:0051346) | 78 | 6 .67 | + | 9.00 | 7.99E-05 | 5.81E-03 |
| negative regulation of proteolysis (GO:0045861) | 68 | 5 .58 | + | 8.60 | 3.89E-04 | 1.97E-02 |
| regulation of endopeptidase activity (GO:0052548) | 84 | 6 .72 | + | 8.36 | 1.17E-04 | 8.01E-03 |
| protein-containing complex disassembly (GO:0032984) | 71 | 5 .61 | + | 8.24 | 4.69E-04 | 2.33E-02 |
| regulation of peptidase activity (GO:0052547) | 86 | 6 .73 | + | 8.16 | 1.33E-04 | 8.51E-03 |
| ribose phosphate metabolic process (GO:0019693) | 163 | 10 1.39 | + | 7.18 | 2.28E-06 | 2.49E-04 |
| purine ribonucleotide metabolic process (GO:0009150) | 149 | 9 1.27 | + | 7.07 | 8.34E-06 | 7.92E-04 |
| purine nucleotide metabolic process (GO:0006163) | 166 | 10 1.42 | + | 7.05 | 2.66E-06 | 2.77E-04 |
| ribonucleotide metabolic process (GO:0009259) | 155 | 9 1.32 | + | 6.79 | 1.13E-05 | 1.02E-03 |
| oxidation-reduction process (GO:0055114) | 228 | 13 1.95 | + | 6.67 | 1.49E-07 | 2.17E-05 |
| regulation of RNA splicing (GO:0043484) | 88 | 5 .75 | + | 6.65 | 1.18E-03 | 4.75E-02 |
| purine-containing compound metabolic process (GO:0072521) | 178 | 10 1.52 | + | 6.57 | 4.80E-06 | 4.76E-04 |
| nucleotide metabolic process (GO:0009117) | 196 | 11 1.67 | + | 6.57 | 1.61E-06 | 1.95E-04 |
| nucleoside phosphate metabolic process (GO:0006753) | 202 | 11 1.73 | + | 6.37 | 2.13E-06 | 2.44E-04 |
| monocarboxylic acid metabolic process (GO:0032787) | 167 | 9 1.43 | + | 6.31 | 1.98E-05 | 1.66E-03 |
| nucleobase-containing small molecule metabolic process (GO:0055086) | 235 | 12 2.01 | + | 5.98 | 1.38E-06 | 1.77E-04 |
| carbohydrate metabolic process (GO:0005975) | 140 | 7 1.20 | + | 5.85 | 2.62E-04 | 1.43E-02 |
| regulation of proteolysis (GO:0030162) | 128 | 6 1.09 | + | 5.49 | 9.97E-04 | 4.18E-02 |
| negative regulation of cellular protein metabolic process (GO:0032269) | 162 | 7 1.38 | + | 5.06 | 6.07E-04 | 2.70E-02 |
| negative regulation of protein metabolic process (GO:0051248) | 168 | 7 1.44 | + | 4.88 | 7.46E-04 | 3.19E-02 |
| actin cytoskeleton organization (GO:0030036) | 268 | 10 2.29 | + | 4.37 | 1.35E-04 | 8.44E-03 |
| actin filament-based process (GO:0030029) | 280 | 10 2.39 | + | 4.18 | 1.91E-04 | 1.12E-02 |
| carboxylic acid metabolic process (GO:0019752) | 338 | 12 2.89 | + | 4.15 | 4.61E-05 | 3.59E-03 |
| oxoacid metabolic process (GO:0043436) | 357 | 12 3.05 | + | 3.93 | 7.64E-05 | 5.75E-03 |
| organophosphate metabolic process (GO:0019637) | 388 | 13 3.32 | + | 3.92 | 3.95E-05 | 3.19E-03 |
| regulation of organelle organization (GO:0033043) | 275 | 9 2.35 | + | 3.83 | 7.32E-04 | 3.19E-02 |
| organic acid metabolic process (GO:0006082) | 375 | 12 3.20 | + | 3.74 | 1.20E-04 | 7.92E-03 |
| protein-containing complex subunit organization (GO:0043933) | 516 | 13 4.41 | + | 2.95 | 5.96E-04 | 2.71E-02 |
| small molecule metabolic process (GO:0044281) | 654 | 16 5.59 | + | 2.86 | 1.93E-04 | 1.11E-02 |
| catabolic process (GO:0009056) | 921 | 22 7.87 | + | 2.80 | 1.66E-05 | 1.45E-03 |
| cellular macromolecule biosynthetic process (GO:0034645) | 2580 | 8 22.05 | - | .36 | 5.29E-04 | 2.46E-02 |
| macromolecule biosynthetic process (GO:0009059) | 2590 | 8 22.13 | - | .36 | 5.29E-04 | 2.51E-02 |
| regulation of transcription by RNA polymerase II (GO:0006357) | 1591 | 2 13.60 | - | .15 | 1.65E-04 | 1.00E-02 |
| transcription by RNA polymerase II (GO:0006366) | 1635 | 2 13.97 | - | .14 | 1.13E-04 | 7.93E-03 |

|  | | **Venn analysis** |
| --- | --- | --- |
| **Names** | **total** | **elements** |
| **C10 BP IR10 BP IR30 BP IR60 BP** | 23 |  |
|  |  | ADP metabolic process (GO:0046031) |
|  |  | purine ribonucleoside diphosphate metabolic process (GO:0009179) |
|  |  | cellular response to chemical stress (GO:0062197) |
|  |  | glycolytic process (GO:0006096) |
|  |  | macromolecule biosynthetic process (GO:0009059) |
|  |  | ribose phosphate metabolic process (GO:0019693) |
|  |  | monocarboxylic acid metabolic process (GO:0032787) |
|  |  | nucleoside diphosphate phosphorylation (GO:0006165) |
|  |  | nucleoside diphosphate metabolic process (GO:0009132) |
|  |  | carbohydrate catabolic process (GO:0016052) |
|  |  | negative regulation of hydrolase activity (GO:0051346) |
|  |  | ATP metabolic process (GO:0046034) |
|  |  | nucleotide phosphorylation (GO:0046939) |
|  |  | oxidation-reduction process (GO:0055114) |
|  |  | purine nucleoside diphosphate metabolic process (GO:0009135) |
|  |  | ribonucleoside diphosphate metabolic process (GO:0009185) |
|  |  | generation of precursor metabolites and energy (GO:0006091) |
|  |  | ATP generation from ADP (GO:0006757) |
|  |  | cellular macromolecule biosynthetic process (GO:0034645) |
|  |  | nucleobase-containing small molecule metabolic process (GO:0055086) |
|  |  | cellular response to oxidative stress (GO:0034599) |
|  |  | response to oxidative stress (GO:0006979) |
| **C10 BP IR10 BP IR30 BP** | 6 | regulation of transcription, DNA-templated (GO:0006355) |
|  |  | RNA biosynthetic process (GO:0032774) |
|  |  | regulation of RNA biosynthetic process (GO:2001141) |
|  |  | nucleic acid-templated transcription (GO:0097659) |
|  |  | transcription, DNA-templated (GO:0006351) |
|  |  | regulation of nucleic acid-templated transcription (GO:1903506) |
| **C10 BP IR30 BP IR60 BP** | 2 | regulation of peptidase activity (GO:0052547) |
|  |  | regulation of endopeptidase activity (GO:0052548) |
| **IR10 BP IR30 BP IR60 BP** | 14 | nucleotide metabolic process (GO:0009117) |
|  |  | protein depolymerization (GO:0051261) |
|  |  | regulation of organelle organization (GO:0033043) |
|  |  | protein-containing complex subunit organization (GO:0043933) |
|  |  | purine ribonucleotide metabolic process (GO:0009150) |
|  |  | catabolic process (GO:0009056) |
|  |  | purine-containing compound metabolic process (GO:0072521) |
|  |  | purine nucleotide metabolic process (GO:0006163) |
|  |  | actin filament-based process (GO:0030029) |
|  |  | transcription by RNA polymerase II (GO:0006366) |
|  |  | nucleoside phosphate metabolic process (GO:0006753) |
|  |  | actin cytoskeleton organization (GO:0030036) |
|  |  | regulation of transcription by RNA polymerase II (GO:0006357) |
|  |  | ribonucleotide metabolic process (GO:0009259) |
| **C10 BP IR10 BP** | 26 | regulation of cellular macromolecule biosynthetic process (GO:2000112) |
|  |  | organic cyclic compound biosynthetic process (GO:1901362) |
|  |  | cellular biosynthetic process (GO:0044249) |
|  |  | anatomical structure morphogenesis (GO:0009653) |
|  |  | transmembrane receptor protein tyrosine kinase signaling pathway (GO:0007169) |
|  |  | extracellular matrix organization (GO:0030198) |
|  |  | aromatic compound biosynthetic process (GO:0019438) |
|  |  | extracellular structure organization (GO:0043062) |
|  |  | tissue development (GO:0009888) |
|  |  | 'de novo' protein folding (GO:0006458) |
|  |  | multicellular organism development (GO:0007275) |
|  |  | cellular nitrogen compound biosynthetic process (GO:0044271) |
|  |  | regulation of cellular biosynthetic process (GO:0031326) |
|  |  | protein folding (GO:0006457) |
|  |  | nucleobase-containing compound biosynthetic process (GO:0034654) |
|  |  | developmental process (GO:0032502) |
|  |  | nucleic acid metabolic process (GO:0090304) |
|  |  | heterocycle biosynthetic process (GO:0018130) |
|  |  | RNA metabolic process (GO:0016070) |
|  |  | regulation of macromolecule biosynthetic process (GO:0010556) |
|  |  | regulation of nucleobase-containing compound metabolic process (GO:0019219) |
|  |  | anatomical structure development (GO:0048856) |
|  |  | regulation of RNA metabolic process (GO:0051252) |
|  |  | regulation of biosynthetic process (GO:0009889) |
|  |  | biosynthetic process (GO:0009058) |
|  |  | organic substance biosynthetic process (GO:1901576) |
| **C10 BP IR60 BP** | 6 | negative regulation of proteolysis (GO:0045861) |
|  |  | negative regulation of protein metabolic process (GO:0051248) |
|  |  | negative regulation of endopeptidase activity (GO:0010951) |
|  |  | negative regulation of cellular protein metabolic process (GO:0032269) |
|  |  | negative regulation of peptidase activity (GO:0010466) |
|  |  | regulation of proteolysis (GO:0030162) |
| **IR10 BP IR30 BP** | 6 | muscle structure development (GO:0061061) |
|  |  | regulation of cellular component organization (GO:0051128) |
|  |  | regulation of cytoskeleton organization (GO:0051493) |
|  |  | supramolecular fiber organization (GO:0097435) |
|  |  | cytoskeleton organization (GO:0007010) |
|  |  | actin filament organization (GO:0007015) |
| **IR10 BP IR60 BP** | 1 | cellular protein complex disassembly (GO:0043624) |
| **IR30 BP IR60 BP** | 9 | organic acid metabolic process (GO:0006082) |
|  |  | protein-containing complex disassembly (GO:0032984) |
|  |  | small molecule metabolic process (GO:0044281) |

|  | | NADP metabolic process (GO:0006739) |
| --- | --- | --- |
|  |  | carbohydrate metabolic process (GO:0005975) |
|  |  | regulation of RNA splicing (GO:0043484) |
|  |  | carboxylic acid metabolic process (GO:0019752) |
|  |  | organophosphate metabolic process (GO:0019637) |
|  |  | oxoacid metabolic process (GO:0043436) |
| **C10 BP** | 12 | chaperone-mediated protein folding (GO:0061077) |
|  |  | negative regulation of catalytic activity (GO:0043086) |
|  |  | macromolecule metabolic process (GO:0043170) |
|  |  | multicellular organismal process (GO:0032501) |
|  |  | regulation of vasculature development (GO:1901342) |
|  |  | negative regulation of molecular function (GO:0044092) |
|  |  | wound healing (GO:0042060) |
|  |  | cell-substrate junction assembly (GO:0007044) |
|  |  | regulation of angiogenesis (GO:0045765) |
|  |  | gene expression (GO:0010467) |
|  |  | response to wounding (GO:0009611) |
|  |  | enzyme linked receptor protein signaling pathway (GO:0007167) |
| **IR10 BP** | 55 | positive regulation of cytoskeleton organization (GO:0051495) |
|  |  | regulation of anatomical structure size (GO:0090066) |
|  |  | locomotion (GO:0040011) |
|  |  | regulation of actin polymerization or depolymerization (GO:0008064) |
|  |  | regulation of actin filament polymerization (GO:0030833) |
|  |  | cell motility (GO:0048870) |
|  |  | regulation of protein stability (GO:0031647) |
|  |  | actin filament polymerization (GO:0030041) |
|  |  | localization of cell (GO:0051674) |
|  |  | regulation of cellular component biogenesis (GO:0044087) |
|  |  | cellular process (GO:0009987) |
|  |  | response to stress (GO:0006950) |
|  |  | positive regulation of supramolecular fiber organization (GO:1902905) |
|  |  | regulation of actin filament-based process (GO:0032970) |
|  |  | positive regulation of actin filament polymerization (GO:0030838) |
|  |  | cell migration (GO:0016477) |
|  |  | protein-containing complex assembly (GO:0065003) |
|  |  | cellular component biogenesis (GO:0044085) |
|  |  | protein polymerization (GO:0051258) |
|  |  | regulation of biological quality (GO:0065008) |
|  |  | regulation of protein polymerization (GO:0032271) |
|  |  | cellular component assembly (GO:0022607) |
|  |  | actin filament bundle assembly (GO:0051017) |
|  |  | regulation of actin cytoskeleton organization (GO:0032956) |
|  |  | regulation of actin filament organization (GO:0110053) |
|  |  | cellular component organization or biogenesis (GO:0071840) |
|  |  | cellular component organization (GO:0016043) |
|  |  | regulation of protein-containing complex assembly (GO:0043254) |
|  |  | cellular protein-containing complex assembly (GO:0034622) |
|  |  | positive regulation of protein-containing complex assembly (GO:0031334) |
|  |  | regulation of cellular component size (GO:0032535) |
|  |  | positive regulation of protein kinase B signaling (GO:0051897) |
|  |  | cellular response to stress (GO:0033554) |
|  |  | positive regulation of cellular component organization (GO:0051130) |
|  |  | response to chemical (GO:0042221) |
|  |  | biological_process (GO:0008150) |
|  |  | animal organ development (GO:0048513) |
|  |  | actin filament bundle organization (GO:0061572) |
|  |  | developmental growth (GO:0048589) |
|  |  | regulation of supramolecular fiber organization (GO:1902903) |
|  |  | regulation of gene expression (GO:0010468) |
|  |  | Unclassified (UNCLASSIFIED) |
|  |  | positive regulation of protein polymerization (GO:0032273) |
|  |  | actin polymerization or depolymerization (GO:0008154) |
|  |  | cellular homeostasis (GO:0019725) |
|  |  | homeostatic process (GO:0042592) |
|  |  | system development (GO:0048731) |
|  |  | regulation of actin filament length (GO:0030832) |
|  |  | response to inorganic substance (GO:0010035) |
|  |  | movement of cell or subcellular component (GO:0006928) |
|  |  | positive regulation of cellular component biogenesis (GO:0044089) |
|  |  | Arp2/3 complex-mediated actin nucleation (GO:0034314) |
|  |  | positive regulation of organelle organization (GO:0010638) |
|  |  | organelle organization (GO:0006996) |
|  |  | protein stabilization (GO:0050821) |
| **IR30 BP** | 11 | cellular catabolic process (GO:0044248) |
|  |  | carbohydrate derivative metabolic process (GO:1901135) |
|  |  | organic substance catabolic process (GO:1901575) |
|  |  | negative regulation of chromatin silencing (GO:0031936) |
|  |  | regulation of gene silencing (GO:0060968) |
|  |  | organonitrogen compound catabolic process (GO:1901565) |
|  |  | regulation of chromatin silencing (GO:0031935) |
|  |  | negative regulation of DNA recombination (GO:0045910) |
|  |  | negative regulation of cellular component organization (GO:0051129) |
|  |  | regulation of mRNA processing (GO:0050684) |
|  |  | regulation of mRNA splicing, via spliceosome (GO:0048024) |
