# Supplementary material for: Progression of irradiated mesenchymal stromal cells from early to late senescence: Changes in SASP composition and anti‐tumour properties
Source: Cell Prolif. 2023 Mar 22;56(6):e13401. doi: 10.1111/cpr.13401 (PMC10280137; doi:10.1111/cpr.13401)
Supplement: Supplementary file 3 — Supplementary file S3. REACTOME analysis. The results of pathway identification by REACTOME test are reported. The analysis was performed on secretomes obtained 10, 30 and 60 days (10D, 30D, 60D) post‐x‐ray treatment of MSCs and from unirradiated MSCs (CT). Venn diagram evaluation was performed to identify common and specific pathways among the several experimental conditions. [file CPR-56-e13401-s003.docx]

**Supplementary file 3 – REACTOME analysis**

The results of pathway identification by REACTOME test are reported. The analysis was performed on secretomes obtained 10, 30, and 60 days (10D, 30D, 60D) post-X-ray treatment of MSCs and from unirradiated MSCs (CT). Venn diagram evaluation was performed to identify common and specific pathways among the several experimental conditions.

**REACTOME CT**

| **Pathway identifier** | **Pathway name** | **#Entities found** | **#Entities total** | **#Interactors found** | **#Interactors total** | **Entities ratio** | **Entities pValue** | **Entities FDR** | **#Reactions found** | **#Reactions total** | **Reactions ratio** | **Species identifier** | **Species name** | **Submitted entities found** | **Mapped entities** | **Submitted entities hit interactor** | **Interacts with** | **Found reaction identifiers** |
| --- | --- | --- | --- | --- | --- | --- | --- | --- | --- | --- | --- | --- | --- | --- | --- | --- | --- | --- |
| **R-HSA-8957275** | Post-translational protein phosphorylation | 22 | 109 | 0 | 0 | 0.007637867003013103 | 1.1102230246251565E-16 | 5.551115123125783E-14 | 1 | 1 | 7.365397363187744E-5 | 9606 | Homo sapiens | APP;ITIH2;SPARC;CSF1;IGFBP5;AHSG;PRKCSH;IGFBP4;IGFBP3;TNC;FN1;LAMC1;FSTL1;CST3;STC2;ALB;MXRA8;NUCB1;IGFBP7;QSOX1;TIMP1;FB | | | | R-HSA-8952289 |
| **R-HSA-3000178** | ECM proteoglycans | 22 | 79 | 6 | 50 | 0.005535701772826011 | 1.1102230246251565E-16 | 5.551115123125783E-14 | 19 | 23 | 0.0016940413935331812 | 9606 | Homo sapiens | APP;SPARC;LAMA2;LUM;SERPINE1;TN | P02452;P24043;P02461;P | BMP1;COL3A1;FN1;DAG1;BMP1;GRN | Q14118;29108;P09486;P98160;P07585 | R-HSA-2467716;R-HSA-4086204;R-HSA- |
| **R-HSA-1474228** | Degradation of the extracellular matrix | 27 | 148 | 13 | 89 | 0.010370681802256324 | 1.1102230246251565E-16 | 5.551115123125783E-14 | 81 | 105 | 0.007733667231347131 | 9606 | Homo sapiens | COL18A1;COL15A1;MMP1;MMP2;COL | P02452;P35555;P12111;P | BMP1;APP;APP;TIMP2;LUM;BMP1;M | P01033;P09958;P16070;P50281;P08253;P35 |  |
| **R-HSA-114608** | Platelet degranulation | 24 | 141 | 3 | 45 | 0.009880176581879336 | 2.220446049250313E-16 | 8.326672684688674E-14 | 6 | 11 | 8.101937099506518E-4 | 9606 | Homo sapiens | LGALS3BP;APP;ECM1;SPARC;HSPA5;A | P01033;Q96C24;Q08380; | APP;VIM;COL1A1 | P11021 | R-HSA-482775;R-HSA-482772;R-HSA-48 |
| **R-HSA-2022090** | Assembly of collagen fibrils and other multimeric structures | 17 | 67 | 0 | 14 | 0.004694835680751174 | 1.9984014443252818E-15 | 5.995204332975845E-13 | 21 | 26 | 0.0019150033144288135 | 9606 | Homo sapiens | COL18A1;COL15A1;MMP1;COL12A1;LOXL3;PCOLCE;COL1A1;COL3A1;COL1A2;BMP1;COL5A1;COL4A2;COL6A2;COL6A1;COL5A2;COL6A3;PLEC | | | | R-HSA-2395302;R-HSA-2022141;R-HSA- |
| **R-HSA-1442490** | Collagen degradation | 16 | 69 | 2 | 12 | 0.004834980029430313 | 3.419486915845482E-14 | 8.548717289613705E-12 | 26 | 34 | 0.002504235103483833 | 9606 | Homo sapiens | COL18A1;COL15A1;MMP1;MMP2;COL | P02452;P02461;P05997;P | APP;TIMP1 | P09958;P14780 | R-HSA-1474197;R-HSA-1564179;R-HSA- |
| **R-HSA-76005** | Response to elevated platelet cytosolic Ca2+ | 24 | 148 | 4 | 105 | 0.010370681802256324 | 1.4777068457760834E-13 | 3.1622926499608184E-11 | 7 | 14 | 0.0010311556308462842 | 9606 | Homo sapiens | LGALS3BP;APP;ECM1;SPARC;HSPA5;A | P01033;Q96C24;Q08380; | BMP1;APP;VIM;COL1A1 | 29108;P11021 | R-HSA-482775;R-HSA-482772;R-HSA-48 |
| **R-HSA-1474244** | Extracellular matrix organization | 47 | 329 | 48 | 739 | 0.02305374535771845 | 2.2681856393091948E-13 | 4.241507145508194E-11 | 224 | 319 | 0.023495617588568902 | 9606 | Homo sapiens | COL18A1;APP;COL15A1;SPARC;LAMA | P02452;P24043;P12814;P | FBN1;APP;ENO1;ACTN1;FLNC;PCOL | P01033;P09958;Q14118;P07996;P07237;P16 |  |
| **R-HSA-381426** | Regulation of Insulin-like Growth Factor (IGF) transport and uptake by Insulin-like Growth Factor Binding Proteins (IGFBPs) | 24 | 127 | 3 | 134 | 0.00889916614112536 | 2.636779683484747E-13 | 4.3770542745846797E-11 | 11 | 14 | 0.0010311556308462842 | 9606 | Homo sapiens | APP;SPARC;ITIH2;CSF1;IGFBP5;AHSG; | P01033;P24593;Q12841;P | YWHAG;GSN;GRN | P22692;P17936 | R-HSA-381461;R-HSA-381543;R-HSA-38 |
| **R-HSA-8948216** | Collagen chain trimerization | 12 | 44 | 0 | 0 | 0.0030831756709410693 | 1.8990364836213303E-12 | 2.8485547254319954E-10 | 9 | 28 | 0.0020623112616925685 | 9606 | Homo sapiens | COL1A1;COL18A1;COL3A1;COL15A1;COL1A2;COL5A1;COL4A2;COL6A2;COL6A1;COL5A2;COL12A1;COL6A3 | | | | R-HSA-8944232;R-HSA-8944216;R-HSA- |
| **R-HSA-1474290** | Collagen formation | 18 | 104 | 2 | 108 | 0.007287506131315255 | 8.887590663420042E-10 | 1.2087123302251257E-7 | 52 | 77 | 0.005671355969654563 | 9606 | Homo sapiens | COL18A1;COL15A1;MMP1;COL12A1;L | P02452;P23284;Q15149;P | COL1A1;GRN | P07237;P50454 |  |
| **R-HSA-1650814** | Collagen biosynthesis and modifying enzymes | 15 | 76 | 2 | 94 | 0.005325485249807301 | 1.531293092327246E-8 | 1.9141163654090576E-6 | 31 | 51 | 0.0037563526552257492 | 9606 | Homo sapiens | COL18A1;COL15A1;COL12A1;PCOLCE | P02452;P23284;P02461;P | COL1A1;GRN | P07237;P50454 | R-HSA-8948232;R-HSA-8944265;R-HSA- |
| **R-HSA-3371568** | Attenuation phase | 9 | 47 | 0 | 0 | 0.0032933921939597784 | 2.3393713077801692E-8 | 2.6902770039471946E-6 | 4 | 5 | 3.682698681593872E-4 | 9606 | Homo sapiens | HSP90AA1;HSP90AB1;HSPA1A |  |  |  | R-HSA-3371554;R-HSA-5324617;R-HSA- |
| **R-HSA-3371571** | HSF1-dependent transactivation | 9 | 59 | 0 | 0 | 0.004134258286034615 | 1.5731867264445754E-7 | 1.6833097972956956E-5 | 5 | 8 | 5.892317890550195E-4 | 9606 | Homo sapiens | HSP90AA1;HSP90AB1;HSPA1A |  |  |  | R-HSA-3371554;R-HSA-5324617;R-HSA- |
| **R-HSA-6798695** | Neutrophil degranulation | 23 | 480 | 0 | 0 | 0.03363464368299348 | 2.3560652651077874E-7 | 2.3560652651077874E-5 | 9 | 10 | 7.365397363187744E-4 | 9606 | Homo sapiens | GRN;HSP90AA1;GSN;HSP90AB1;ANXA2;AHSG;MMP1;HMGB1;PRDX6;EEF1A1;CST3;PKM;TIMP2;PTX3;QSOX1;CD248;ALDOA;PPIA;VCL;S100A11;H | | | | R-HSA-6798743;R-HSA-6799350;R-HSA- |
| **R-HSA-3000157** | Laminin interactions | 7 | 31 | 1 | 2 | 0.0021722374045266626 | 4.405647314742467E-7 | 4.046788849798588E-5 | 13 | 15 | 0.0011048096044781615 | 9606 | Homo sapiens | COL18A1;LAMA2;COL4A2;LAMC1;NID1 | P24043;P39060;P08572;Q | GRN | P98160 | R-HSA-4084507;R-HSA-2426530;R-HSA- |
| **R-HSA-6785807** | Interleukin-4 and Interleukin-13 signaling | 19 | 211 | 6 | 153 | 0.014785228785649219 | 4.598623692952941E-7 | 4.046788849798588E-5 | 7 | 47 | 0.0034617367606982396 | 9606 | Homo sapiens | HSP90AA1;ANXA1;COL1A2;MMP1;MM | P01033;P07900;Q16658;P | HSP90AB1;APP;HSPA5;COL1A1;ECM | P48023;P29597;P16671;P14625 | R-HSA-6785895;R-HSA-6790038;R-HSA- |
| **R-HSA-3000171** | Non-integrin membrane-ECM interactions | 16 | 61 | 19 | 204 | 0.004274402634713756 | 6.356813786467796E-7 | 5.276155442768271E-5 | 15 | 22 | 0.0016203874199013037 | 9606 | Homo sapiens | LAMA2;ACTN1;TNC;FN1;LAMC1;THBS | P02452;P24043;P02461;P | ACTB;APP;ACTN1;FLNC;TLN1;PCOLC | 18348;P07996;P12814;P01137;P24821;P090 | R-HSA-2731147;R-HSA-2328129;R-HSA- |
| **R-HSA-2243919** | Crosslinking of collagen fibrils | 6 | 24 | 0 | 0 | 0.0016817321841496743 | 1.1651079109276097E-6 | 9.087841705235356E-5 | 13 | 13 | 9.575016572144067E-4 | 9606 | Homo sapiens | COL1A1;COL1A2;BMP1;COL4A2;LOXL3;PCOLCE | |  |  | R-HSA-2395302;R-HSA-2022141;R-HSA- |
| **R-HSA-419037** | NCAM1 interactions | 8 | 44 | 3 | 27 | 0.0030831756709410693 | 6.358596668754046E-6 | 4.7689475015655347E-4 | 4 | 10 | 7.365397363187744E-4 | 9606 | Homo sapiens | COL3A1;COL5A1;COL4A2;COL6A2;CO | P12110;P02461;P05997;P | APP;APP;PRDX1 | P04156;Q02246 | R-HSA-375157;R-HSA-375155;R-HSA-37 |
| **R-HSA-1592389** | Activation of Matrix Metalloproteinases | 8 | 35 | 7 | 45 | 0.0024525261018849415 | 9.469371158643547E-6 | 6.723253522636918E-4 | 23 | 27 | 0.0019886572880606908 | 9606 | Homo sapiens | COL18A1;MMP1;MMP2;TIMP2;TIMP1 | P01033;P16035;P03956;P | APP;MMP2;APP;TIMP1;TIMP2;COL5A | P01033;P09958;P50281;P08253;P14780;P16 | R-HSA-1604731;R-HSA-1604732;R-HSA- |
| **R-HSA-4420332** | Defective B3GALT6 causes EDSP2 and SEMDJL1 | 5 | 21 | 0 | 0 | 0.001471515661130965 | 1.1714308439270127E-5 | 7.614300485525582E-4 | 1 | 1 | 7.365397363187744E-5 | 9606 | Homo sapiens | GPC1;BGN;AGRN;HSPG2;DCN |  |  |  | R-HSA-4420365 |
| **R-HSA-3560783** | Defective B4GALT7 causes EDS, progeroid type | 5 | 21 | 0 | 0 | 0.001471515661130965 | 1.1714308439270127E-5 | 7.614300485525582E-4 | 1 | 1 | 7.365397363187744E-5 | 9606 | Homo sapiens | GPC1;BGN;AGRN;HSPG2;DCN |  |  |  | R-HSA-3560804 |
| **R-HSA-3560801** | Defective B3GAT3 causes JDSSDHD | 5 | 22 | 0 | 0 | 0.0015415878354705346 | 1.4620415235366835E-5 | 9.064657445927438E-4 | 1 | 1 | 7.365397363187744E-5 | 9606 | Homo sapiens | GPC1;BGN;AGRN;HSPG2;DCN |  |  |  | R-HSA-3560802 |
| **R-HSA-6802948** | Signaling by high-kinase activity BRAF mutants | 6 | 44 | 0 | 0 | 0.0030831756709410693 | 3.532146511808776E-5 | 0.0021192879070852655 | 4 | 6 | 4.419238417912646E-4 | 9606 | Homo sapiens | FN1;PEBP1;TLN1;VCL;ACTB;ACTG1 |  |  |  | R-HSA-6802912;R-HSA-6802911;R-HSA- |
| **R-HSA-3371511** | HSF1 activation | 8 | 43 | 3 | 56 | 0.0030131034966014996 | 5.480930515122662E-5 | 0.003124130393619917 | 5 | 7 | 5.155778154231421E-4 | 9606 | Homo sapiens | EEF1A1;HSP90AA1;HSP90AB1;HSPA1A | P07900;ENSG0000020438 | HSP90AB1;HSP90AA1;YWHAZ | P68104;Q15185 | R-HSA-3371586;R-HSA-4793819;R-HSA- |
| **R-HSA-2214320** | Anchoring fibril formation | 4 | 15 | 0 | 0 | 0.0010510826150935463 | 5.8597157786111076E-5 | 0.003222843678236109 | 2 | 4 | 2.9461589452750976E-4 | 9606 | Homo sapiens | COL1A1;COL1A2;BMP1;COL4A2 |  |  |  | R-HSA-2214330;R-HSA-2396234 |
| **R-HSA-5674135** | MAP2K and MAPK activation | 6 | 49 | 0 | 0 | 0.0034335365426389182 | 6.369745323231424E-5 | 0.0033759650213126546 | 8 | 12 | 8.838476835825292E-4 | 9606 | Homo sapiens | FN1;PEBP1;TLN1;VCL;ACTB;ACTG1 |  |  |  | R-HSA-9657603;R-HSA-5672980;R-HSA- |
| **R-HSA-6802952** | Signaling by BRAF and RAF1 fusions | 7 | 73 | 0 | 0 | 0.005115268726788592 | 7.227336882342783E-5 | 0.0036859418099948194 | 5 | 5 | 3.682698681593872E-4 | 9606 | Homo sapiens | LMNA;FN1;PEBP1;TLN1;VCL;ACTB;ACTG1 | |  |  | R-HSA-6802935;R-HSA-6802934;R-HSA- |
| **R-HSA-76002** | Platelet activation, signaling and aggregation | 27 | 293 | 30 | 644 | 0.02053114708149394 | 9.159533491753802E-5 | 0.004579766745876901 | 55 | 116 | 0.008543860941297783 | 9606 | Homo sapiens | LGALS3BP;APP;ECM1;SPARC;SERPIN | P02452;Q08380;P62937;P | APP;HSPA5;ACTN1;FLNC;PEBP1;ACT | P04049;P56945;Q16539;P21333;O15530;P49 |  |
| **R-HSA-3560782** | Diseases associated with glycosaminoglycan metabolism | 6 | 55 | 0 | 1 | 0.0038539695886763365 | 1.1909831119261138E-4 | 0.005716718937245346 | 20 | 29 | 0.0021359652353244458 | 9606 | Homo sapiens | LUM;GPC1;BGN;AGRN;HSPG2;DCN |  |  |  | R-HSA-3636919;R-HSA-9035949;R-HSA- |
| **R-HSA-1971475** | A tetrasaccharide linker sequence is required for GAG synthesis | 5 | 31 | 0 | 6 | 0.0021722374045266626 | 1.668852387233244E-4 | 0.007509835742549598 | 5 | 5 | 3.682698681593872E-4 | 9606 | Homo sapiens | GPC1;BGN;AGRN;HSPG2;DCN |  |  |  | R-HSA-1889981;R-HSA-9638064;R-HSA- |
| **R-HSA-2129379** | Molecules associated with elastic fibres | 5 | 38 | 0 | 2 | 0.0026627426249036506 | 2.3848498396539242E-4 | 0.010330705414706376 | 7 | 10 | 7.365397363187744E-4 | 9606 | Homo sapiens | LTBP4;FN1;FBLN1;EMILIN1;FBN1 |  |  |  | R-HSA-2395364;R-HSA-2161282;R-HSA- |
| **R-HSA-1566948** | Elastic fibre formation | 6 | 46 | 3 | 22 | 0.0032233200196202087 | 2.45969176540628E-4 | 0.010330705414706376 | 14 | 17 | 0.0012521175517419166 | 9606 | Homo sapiens | LTBP4;LOXL3;FN1;FBLN1;EMILIN1;FBN | Q9Y6C2;P23142;Q8N2S1; | FBN1;APP;GRN | P09958;P15502;Q9UBX5 | R-HSA-2395364;R-HSA-2161282;R-HSA- |
| **R-HSA-8874081** | MET activates PTK2 signaling | 8 | 32 | 2 | 91 | 0.0022423095788662323 | 2.663388463197691E-4 | 0.010919892699110534 | 2 | 5 | 3.682698681593872E-4 | 9606 | Homo sapiens | COL1A1;COL3A1;COL1A2;LAMA2;COL | P02452;P24043;P02461;P | HSP90AA1;ACTN1 | P12931 | R-HSA-8874083;R-HSA-8874079 |
| **R-HSA-70171** | Glycolysis | 8 | 110 | 0 | 17 | 0.007707939177352673 | 3.135061641200698E-4 | 0.012540246564802793 | 5 | 24 | 0.0017676953671650585 | 9606 | Homo sapiens | PKM;PGK1;ENO1;ALDOA;GAPDH |  |  |  | R-HSA-70449;R-HSA-71670;R-HSA-7149 |
| **R-HSA-216083** | Integrin cell surface interactions | 17 | 86 | 29 | 409 | 0.006026206993202999 | 3.302405336658154E-4 | 0.012590762809700173 | 26 | 55 | 0.004050968549753259 | 9606 | Homo sapiens | COL18A1;LUM;TNC;FN1;THBS1;HSPG | P02452;P02461;P05997;P | FBN1;ENO1;PCOLCE;LASP1;DCN;HS | P07996;P02751;P16070;P35968;P24821;P98 | R-HSA-114563;R-HSA-210304;R-HSA-21 |
| **R-HSA-9706574** | RHOBTB GTPase Cycle | 5 | 36 | 1 | 7 | 0.002522598276224511 | 3.3133586341316246E-4 | 0.012590762809700173 | 4 | 4 | 2.9461589452750976E-4 | 9606 | Homo sapiens | HSP90AA1;HSP90AB1;ACTN1;VIM;ACT | P07900;P63261;P08670;P | YWHAG | O94844 | R-HSA-9018778;R-HSA-9018785;R-HSA- |
| **R-HSA-9013418** | RHOBTB2 GTPase cycle | 4 | 24 | 0 | 0 | 0.0016817321841496743 | 3.491795274924714E-4 | 0.012919642517221441 | 2 | 2 | 1.4730794726375488E-4 | 9606 | Homo sapiens | HSP90AA1;HSP90AB1;ACTN1;ACTG1 |  |  |  | R-HSA-9018785;R-HSA-9018787 |
| **R-HSA-6802955** | Paradoxical activation of RAF signaling by kinase inactive BRAF | 6 | 54 | 0 | 18 | 0.0037838974143367668 | 4.956399400914968E-4 | 0.01641394105438343 | 4 | 7 | 5.155778154231421E-4 | 9606 | Homo sapiens | FN1;PEBP1;TLN1;VCL;ACTB;ACTG1 |  |  |  | R-HSA-6802918;R-HSA-6803234;R-HSA- |
| **R-HSA-6802946** | Signaling by moderate kinase activity BRAF mutants | 6 | 54 | 0 | 18 | 0.0037838974143367668 | 4.956399400914968E-4 | 0.01641394105438343 | 4 | 7 | 5.155778154231421E-4 | 9606 | Homo sapiens | FN1;PEBP1;TLN1;VCL;ACTB;ACTG1 |  |  |  | R-HSA-6802919;R-HSA-6802914;R-HSA- |
| **R-HSA-9649948** | Signaling downstream of RAS mutants | 6 | 54 | 0 | 18 | 0.0037838974143367668 | 4.956399400914968E-4 | 0.01641394105438343 | 4 | 7 | 5.155778154231421E-4 | 9606 | Homo sapiens | FN1;PEBP1;TLN1;VCL;ACTB;ACTG1 |  |  |  | R-HSA-6803233;R-HSA-6802926;R-HSA- |
| **R-HSA-6802949** | Signaling by RAS mutants | 6 | 54 | 0 | 18 | 0.0037838974143367668 | 4.956399400914968E-4 | 0.01641394105438343 | 4 | 9 | 6.628857626868969E-4 | 9606 | Homo sapiens | FN1;PEBP1;TLN1;VCL;ACTB;ACTG1 |  |  |  | R-HSA-6803233;R-HSA-6802926;R-HSA- |
| **R-HSA-8875878** | MET promotes cell motility | 8 | 45 | 2 | 93 | 0.003153247845280639 | 4.973921531631342E-4 | 0.01641394105438343 | 2 | 12 | 8.838476835825292E-4 | 9606 | Homo sapiens | COL1A1;COL3A1;COL1A2;LAMA2;COL | P02452;P24043;P02461;P | HSP90AA1;ACTN1 | P12931 | R-HSA-8874083;R-HSA-8874079 |
| **R-HSA-9662361** | Sensory processing of sound by outer hair cells of the cochlea | 6 | 64 | 1 | 9 | 0.004484619157732465 | 5.324299831375168E-4 | 0.017037759460400537 | 4 | 8 | 5.892317890550195E-4 | 9606 | Homo sapiens | GSN;MYH9;MSN;EZR;ACTB;ACTG1 | P15311;P63261;P60709;P | BMP1 | 29108 | R-HSA-9663363;R-HSA-9662114;R-HSA- |
| **R-HSA-9656223** | Signaling by RAF1 mutants | 5 | 49 | 0 | 0 | 0.0034335365426389182 | 5.963168613621894E-4 | 0.018485822702227872 | 4 | 7 | 5.155778154231421E-4 | 9606 | Homo sapiens | FN1;TLN1;VCL;ACTB;ACTG1 |  |  |  | R-HSA-9656211;R-HSA-9656209;R-HSA- |
| **R-HSA-6802957** | Oncogenic MAPK signaling | 7 | 93 | 0 | 18 | 0.006516712213579987 | 8.820739200053884E-4 | 0.02726662871691876 | 25 | 46 | 0.0033880827870663623 | 9606 | Homo sapiens | LMNA;FN1;PEBP1;TLN1;VCL;ACTB;ACTG1 | |  |  | R-HSA-9656211;R-HSA-6802919;R-HSA- |
| **R-HSA-70263** | Gluconeogenesis | 6 | 66 | 0 | 15 | 0.004624763506411604 | 9.08887623897292E-4 | 0.02726662871691876 | 4 | 26 | 0.0019150033144288135 | 9606 | Homo sapiens | PGK1;ENO1;ALDOA;GAPDH |  |  |  | R-HSA-70482;R-HSA-71495;R-HSA-7048 |
| **R-HSA-3656253** | Defective EXT1 causes exostoses 1, TRPS2 and CHDS | 3 | 16 | 0 | 0 | 0.0011211547894331162 | 0.0014186080794453826 | 0.041139634303916095 | 4 | 4 | 2.9461589452750976E-4 | 9606 | Homo sapiens | GPC1;AGRN;HSPG2 |  |  |  | R-HSA-9036283;R-HSA-3656261;R-HSA- |
| **R-HSA-3656237** | Defective EXT2 causes exostoses 2 | 3 | 16 | 0 | 0 | 0.0011211547894331162 | 0.0014186080794453826 | 0.041139634303916095 | 4 | 4 | 2.9461589452750976E-4 | 9606 | Homo sapiens | GPC1;AGRN;HSPG2 |  |  |  | R-HSA-9036289;R-HSA-3656254;R-HSA- |

1

**REACTOME 10D**

| **Pathway identifier** | **Pathway name** | **#Entities found** | **#Entities total** | **#Interactors found** | **#Interactors total** | **Entities ratio** | **Entities pValue** | **Entities FDR** | **#Reactions found** | **#Reactions total** | **Reactions ratio** | **Species identifier** | **Species name** | **Submitted entities found** | **Mapped entities** | **Submitted entities hit interactor** | **Interacts with** | **Found reaction identifiers** |
| --- | --- | --- | --- | --- | --- | --- | --- | --- | --- | --- | --- | --- | --- | --- | --- | --- | --- | --- |
| **R-HSA-8957275** | Post-translational protein phosphorylation | 26 | 109 | 0 | 0 | 0.007637867003013103 | 1.1102230246251565E-16 | 1.887379141862766E-13 | 1 | 1 | 7.365397363187744E-5 | 9606 | Homo sapiens | APP;SPARC;ITIH2;PRKCSH;LAMC1;HSP90B1;CST3;C3;LGALS1;STC2;QSOX1;IGFBP7;TIMP1;CCN1;IGFBP5;AHSG;LAMB2;IGFBP4;IGF | | | | R-HSA-8952289 |
| **R-HSA-6798695** | Neutrophil degranulation | 51 | 480 | 0 | 0 | 0.03363464368299348 | 2.220446049250313E-16 | 1.887379141862766E-13 | 9 | 10 | 7.365397363187744E-4 | 9606 | Homo sapiens | GPI;HSP90AB1;HEXB;LGALS3;TIMP2;QSOX1;B2M;CTSD;SPTAN1;CTSB;CAP1;ACTR2;HSP90AA1;ANXA2;AHSG;MMP1;PGAM1;KRT1;M | | | | R-HSA-6798743;R-HSA-6799350;R-H |
| **R-HSA-114608** | Platelet degranulation | 25 | 141 | 5 | 45 | 0.009880176581879336 | 1.0477763101590654E-10 | 4.9980442007324655E-8 | 5 | 11 | 8.101937099506518E-4 | 9606 | Homo sapiens | LGALS3BP;APP;ECM1;SPARC; | P55145;Q08380;P62937;O0039 | APP;VIM;COL1A1;PDIA6;HSP90B1 | P11021 | R-HSA-482775;R-HSA-482772;R-HS |
| **R-HSA-1442490** | Collagen degradation | 17 | 69 | 2 | 12 | 0.004834980029430313 | 1.1760104001723448E-10 | 4.9980442007324655E-8 | 25 | 34 | 0.002504235103483833 | 9606 | Homo sapiens | MMP1;MMP2;COL12A1;MMP3; | P02452;P02461;P05997;P0733 | APP;TIMP1 | P09958;P14780 | R-HSA-1474197;R-HSA-1564179;R-H |
| **R-HSA-381426** | Regulation of Insulin-like Growth Factor (IGF) transport and uptake by Insulin-like Growth Factor Binding Proteins (IGFBPs) | 29 | 127 | 4 | 134 | 0.00889916614112536 | 2.1813872930209754E-10 | 7.416716796271317E-8 | 12 | 14 | 0.0010311556308462842 | 9606 | Homo sapiens | APP;SPARC;ITIH2;PRKCSH;LA | P55268;P14314;P11047;O0062 | YWHAG;IGFBP6;GSN;GRN | P24592;P22692;P17936 | R-HSA-381461;R-HSA-381543;R-HS |
| **R-HSA-3000178** | ECM proteoglycans | 20 | 79 | 3 | 50 | 0.005535701772826011 | 5.302485117653077E-10 | 1.500603288295821E-7 | 16 | 23 | 0.0016940413935331812 | 9606 | Homo sapiens | APP;SPARC;LAMB2;LUM;LAMA | P02452;P02461;P05997;P5526 | COL3A1;DAG1;GRN | Q14118;P09486;P98160 | R-HSA-2467716;R-HSA-4086204;R-H |
| **R-HSA-2022090** | Assembly of collagen fibrils and other multimeric structures | 15 | 67 | 0 | 14 | 0.004694835680751174 | 7.538355384362205E-9 | 1.8318203584000159E-6 | 20 | 26 | 0.0019150033144288135 | 9606 | Homo sapiens | MMP1;COL12A1;MMP3;PCOLCE;COL1A1;COL3A1;COL1A2;LOX;COL4A2;COL6A2;COL6A1;COL5A2;COL6A3;CTSB;PLEC | | | | R-HSA-2395302;R-HSA-2022141;R-H |
| **R-HSA-3371568** | Attenuation phase | 12 | 47 | 0 | 0 | 0.0032933921939597784 | 9.99419569325255E-9 | 2.1187694869695406E-6 | 4 | 5 | 3.682698681593872E-4 | 9606 | Homo sapiens | HSPA8;HSP90AA1;HSP90AB1;SERPINH1;HSPB1;HSPA1A | |  |  | R-HSA-3371554;R-HSA-5324617;R-H |
| **R-HSA-76005** | Response to elevated platelet cytosolic Ca2+ | 25 | 148 | 6 | 105 | 0.010370681802256324 | 3.896088995869462E-8 | 7.363608202193284E-6 | 6 | 14 | 0.0010311556308462842 | 9606 | Homo sapiens | LGALS3BP;APP;ECM1;SPARC; | P55145;Q08380;P62937;O0039 | STX7;APP;VIM;COL1A1;PDIA6;HSP90B1 | Q12846;P11021 | R-HSA-482775;R-HSA-482772;R-HS |
| **R-HSA-1474228** | Degradation of the extracellular matrix | 23 | 148 | 10 | 89 | 0.010370681802256324 | 9.26376237941895E-8 | 1.5748396045012214E-5 | 73 | 105 | 0.007733667231347131 | 9606 | Homo sapiens | MMP1;LAMB2;MMP2;COL12A1 | P01033;P02452;P02461;P0599 | APP;MMP2;LGALS3;APP;TIMP1;MSN;TIM | P09958;P16070;P50281;P092 |  |
| **R-HSA-3371571** | HSF1-dependent transactivation | 12 | 59 | 0 | 0 | 0.004134258286034615 | 1.1449134340590916E-7 | 1.763166688451001E-5 | 5 | 8 | 5.892317890550195E-4 | 9606 | Homo sapiens | HSPA8;HSP90AA1;HSP90AB1;SERPINH1;HSPB1;HSPA1A | |  |  | R-HSA-3371554;R-HSA-5324617;R-H |
| **R-HSA-8948216** | Collagen chain trimerization | 9 | 44 | 0 | 0 | 0.0030831756709410693 | 4.1753427533208765E-6 | 5.887233282182436E-4 | 7 | 28 | 0.0020623112616925685 | 9606 | Homo sapiens | COL1A1;COL3A1;COL1A2;COL4A2;COL6A2;COL6A1;COL5A2;COL12A1;COL6A3 | | |  | R-HSA-8944232;R-HSA-8944265;R-H |
| **R-HSA-3371497** | HSP90 chaperone cycle for steroid hormone receptors (SHR) in the presence of ligand | 14 | 72 | 5 | 50 | 0.005045196552449022 | 5.846701168765733E-6 | 7.148510960007792E-4 | 16 | 16 | 0.001178463578110039 | 9606 | Homo sapiens | CAP1;TUBA1C;STIP1;HSPA8;D | P0DMV8;P07900;P0DMV9;Q3Z | HSP90AB1;HSP90AA1;HSPB1;S100A6;C | Q13451;Q02790;P31948;Q151 | R-HSA-5618099;R-HSA-5618098;R-H |
| **R-HSA-1474290** | Collagen formation | 19 | 104 | 3 | 108 | 0.007287506131315255 | 5.907860297527101E-6 | 7.148510960007792E-4 | 49 | 77 | 0.005671355969654563 | 9606 | Homo sapiens | MMP1;COL12A1;MMP3;PLOD3 | P02452;P23284;Q15149;P0246 | COL1A1;GRN;AK2 | P07237;P50454 |  |
| **R-HSA-6785807** | Interleukin-4 and Interleukin-13 signaling | 25 | 211 | 9 | 153 | 0.014785228785649219 | 1.5507366060041328E-5 | 0.00175233236478467 | 10 | 47 | 0.0034617367606982396 | 9606 | Homo sapiens | HSPA8;HSP90AA1;ANXA1;MMP | P07900;ENSG00000135046;EN | HSP90AB1;HNRNPK;APP;LGALS3;HNRN | P48023;Q14627;P29597;P166 | R-HSA-6785895;R-HSA-6790038;R-H |
| **R-HSA-70263** | Gluconeogenesis | 11 | 66 | 1 | 15 | 0.004624763506411604 | 1.7149906173075102E-5 | 0.0018178900543459608 | 9 | 26 | 0.0019150033144288135 | 9606 | Homo sapiens | GPI;MDH1;MDH2;PGAM1;PGK1 | P06733;P06744;P07205;P1866 | HSPD1 | Q16822 | R-HSA-70482;R-HSA-71445;R-HSA- |
| **R-HSA-3560783** | Defective B4GALT7 causes EDS, progeroid type | 6 | 21 | 0 | 0 | 0.001471515661130965 | 2.7385561213399967E-5 | 0.002574242754059597 | 1 | 1 | 7.365397363187744E-5 | 9606 | Homo sapiens | GPC1;BGN;CSPG4;AGRN;HSPG2;DCN | |  |  | R-HSA-3560804 |
| **R-HSA-4420332** | Defective B3GALT6 causes EDSP2 and SEMDJL1 | 6 | 21 | 0 | 0 | 0.001471515661130965 | 2.7385561213399967E-5 | 0.002574242754059597 | 1 | 1 | 7.365397363187744E-5 | 9606 | Homo sapiens | GPC1;BGN;CSPG4;AGRN;HSPG2;DCN | |  |  | R-HSA-4420365 |
| **R-HSA-3560801** | Defective B3GAT3 causes JDSSDHD | 6 | 22 | 0 | 0 | 0.0015415878354705346 | 3.53935425285723E-5 | 0.0031500252850429344 | 1 | 1 | 7.365397363187744E-5 | 9606 | Homo sapiens | GPC1;BGN;CSPG4;AGRN;HSPG2;DCN | |  |  | R-HSA-3560802 |
| **R-HSA-9013418** | RHOBTB2 GTPase cycle | 6 | 24 | 0 | 0 | 0.0016817321841496743 | 5.702088449388576E-5 | 0.0048467751819802896 | 2 | 2 | 1.4730794726375488E-4 | 9606 | Homo sapiens | HSP90AA1;HSP90AB1;CDC37;TMOD3;RBMX;ACTG1 | |  |  | R-HSA-9018785;R-HSA-9018787 |
| **R-HSA-3560782** | Diseases associated with glycosaminoglycan metabolism | 8 | 55 | 1 | 1 | 0.0038539695886763365 | 1.503363629258292E-4 | 0.012177245396992165 | 26 | 29 | 0.0021359652353244458 | 9606 | Homo sapiens | LUM;HEXB;GPC1;BGN;CSPG4; | P35052;P21810;Q6UVK1;P0758 | HEXB | P06865 | R-HSA-3636919;R-HSA-9035976;R-H |
| **R-HSA-9706574** | RHOBTB GTPase Cycle | 7 | 36 | 1 | 7 | 0.002522598276224511 | 1.9719757564073959E-4 | 0.014672546099534944 | 4 | 4 | 2.9461589452750976E-4 | 9606 | Homo sapiens | HSP90AA1;HSP90AB1;CDC37; | Q9NYL9;P07900;P63261;P0867 | YWHAG | O94844 | R-HSA-9018778;R-HSA-9018785;R-H |
| **R-HSA-9662361** | Sensory processing of sound by outer hair cells of the cochlea | 9 | 64 | 0 | 9 | 0.004484619157732465 | 2.009937821854102E-4 | 0.014672546099534944 | 1 | 8 | 5.892317890550195E-4 | 9606 | Homo sapiens | GSN;RDX;MYH9;MSN;EZR;SPTAN1;SPTBN1;ACTB;ACTG1 | |  |  | R-HSA-9663363 |
| **R-HSA-1592389** | Activation of Matrix Metalloproteinases | 9 | 35 | 7 | 45 | 0.0024525261018849415 | 2.219140644060813E-4 | 0.015394141355455027 | 24 | 27 | 0.0019886572880606908 | 9606 | Homo sapiens | MMP1;CTSK;MMP2;MMP3;TIM | P01033;P16035;P03956;P2289 | APP;MMP2;LGALS3;APP;TIMP1;TIMP2;L | P09958;P50281;P09237;P082 | R-HSA-1604763;R-HSA-1604731;R-H |
| **R-HSA-5626467** | RHO GTPases activate IQGAPs | 7 | 36 | 1 | 9 | 0.002522598276224511 | 2.2638443169786804E-4 | 0.015394141355455027 | 5 | 5 | 3.682698681593872E-4 | 9606 | Homo sapiens | TUBA1C;IQGAP1;TUBB4B;ACT | P63261;Q3ZCM7;P60709;P469 | MYH9 | O00255 | R-HSA-5626549;R-HSA-5626469;R-H |
| **R-HSA-1650814** | Collagen biosynthesis and modifying enzymes | 14 | 76 | 3 | 94 | 0.005325485249807301 | 2.4529985217047745E-4 | 0.015944490391081034 | 29 | 51 | 0.0037563526552257492 | 9606 | Homo sapiens | COL12A1;PLOD3;PCOLCE;COL | P02452;P23284;P02461;P0599 | COL1A1;GRN;AK2 | P07237;P50454 |  |
| **R-HSA-3000157** | Laminin interactions | 6 | 31 | 1 | 2 | 0.0021722374045266626 | 3.14626979304955E-4 | 0.018979805628189794 | 13 | 15 | 0.0011048096044781615 | 9606 | Homo sapiens | COL4A2;LAMB2;LAMA4;LAMC1 | P55268;P08572;Q14112;P9816 | GRN | P98160 | R-HSA-4084507;R-HSA-2426530;R-H |
| **R-HSA-3371511** | HSF1 activation | 10 | 43 | 4 | 56 | 0.0030131034966014996 | 3.1633009380316324E-4 | 0.018979805628189794 | 6 | 7 | 5.155778154231421E-4 | 9606 | Homo sapiens | VCP;HSP90AA1;HSP90AB1;SE | P07900;ENSG00000204389;EN | HSP90AB1;BAG3;HSP90AA1;YWHAZ | P68104;Q00613;Q15185 | R-HSA-3371586;R-HSA-4793819;R-H |
| **R-HSA-2243919** | Crosslinking of collagen fibrils | 5 | 24 | 0 | 0 | 0.0016817321841496743 | 5.424628860927205E-4 | 0.030854008540557754 | 13 | 13 | 9.575016572144067E-4 | 9606 | Homo sapiens | COL1A1;COL1A2;LOX;COL4A2;PCOLCE | |  |  | R-HSA-2395302;R-HSA-2022141;R-H |
| **R-HSA-1971475** | A tetrasaccharide linker sequence is required for GAG synthesis | 6 | 31 | 0 | 6 | 0.0021722374045266626 | 5.713705285288473E-4 | 0.030854008540557754 | 5 | 5 | 3.682698681593872E-4 | 9606 | Homo sapiens | GPC1;BGN;CSPG4;AGRN;HSPG2;DCN | |  |  | R-HSA-1889981;R-HSA-9638064;R-H |
| **R-HSA-437239** | Recycling pathway of L1 | 11 | 55 | 2 | 74 | 0.0038539695886763365 | 6.246250018082655E-4 | 0.03185587509222154 | 13 | 14 | 0.0010311556308462842 | 9606 | Homo sapiens | TUBA1C;DPYSL2;RDX;MSN;CL | P15311;P63261;Q3ZCM7;P607 | APP;PSMA1 | P28482 | R-HSA-443783;R-HSA-443779;R-HS |
| **R-HSA-70171** | Glycolysis | 11 | 110 | 0 | 17 | 0.007707939177352673 | 6.246250018082655E-4 | 0.03185587509222154 | 7 | 24 | 0.0017676953671650585 | 9606 | Homo sapiens | GPI;PKM;PGAM1;PGK1;ALDOC;ENO1;ALDOA;GAPDH | |  |  | R-HSA-70449;R-HSA-70471;R-HSA- |
| **R-HSA-70326** | Glucose metabolism | 13 | 140 | 1 | 32 | 0.009810104407539766 | 7.931601774323616E-4 | 0.03965800887161808 | 16 | 50 | 0.003682698681593872 | 9606 | Homo sapiens | GPI;PKM;MDH1;MDH2;PGAM1; | P06733;P06744;P07205;P4092 | HSPD1 | Q16822 | R-HSA-71495;R-HSA-70471;R-HSA- |

REACTOME 30D

| **Pathway identifier** | **Pathway name** | **#Entities found** | **#Entities total** | **#Interactors found** | **#Interactors total** | **Entities ratio** | **Entities pValue** | **Entities FDR** | **#Reactions found** | **#Reactions total** | **Reactions ratio** | **Species identifier** | **Species name** | **Submitted entities found** | **Mapped entities** | **Submitted entities hit interactor** | **Interacts with** | **Found reaction identifiers** |
| --- | --- | --- | --- | --- | --- | --- | --- | --- | --- | --- | --- | --- | --- | --- | --- | --- | --- | --- |
| **R-HSA-1236977** | Endosomal/Vacuolar pathway | 37 | 82 | 0 | 0 | 0.00574591829584472 | 1.1102230246251565E-16 | 9.880984919163893E-14 | 4 | 4 | 2.9461589452750976E-4 | 9606 | Homo sapiens | HLA-C;HLA-A;B2M;CTSS |  |  |  | R-HSA-1236964;R-HSA-12369 |
| **R-HSA-6798695** | Neutrophil degranulation | 53 | 480 | 0 | 0 | 0.03363464368299348 | 1.1102230246251565E-16 | 9.880984919163893E-14 | 7 | 10 | 7.365397363187744E-4 | 9606 | Homo sapiens | GPI;HEXB;CTSS;LGALS3;TIMP2;COTL1;QSOX1;B2M;CTSD;SPTAN1;CTSB;CAP1;CBR1;ANXA2;AHSG;MMP1;PGA | | | | R-HSA-6798743;R-HSA-68004 |
| **R-HSA-983170** | Antigen Presentation: Folding, assembly and peptide loading of class I MHC | 38 | 103 | 11 | 164 | 0.007217433956975685 | 2.220446049250313E-16 | 1.3167245072054357E-13 | 13 | 16 | 0.001178463578110039 | 9606 | Homo sapiens | PDIA3;HSPA5;HLA-C;HLA-A;B2 | P30501;P30447;P305 | B2M;PDIA3;APP;IDH1;HLA-C;TUBB;CO | O15533;P30101;P | R-HSA-983148;R-HSA-895149 |
| **R-HSA-1236974** | ER-Phagosome pathway | 43 | 173 | 8 | 195 | 0.012122486160745568 | 4.6629367034256575E-15 | 2.0750068330244176E-12 | 7 | 10 | 7.365397363187744E-4 | 9606 | Homo sapiens | PDIA3;PSMA6;PSMA1;PSME1;H | P30501;P30447;P305 | STX7;PDIA3;APP;IDH1;TUBB;HLA-A;HS | O15533;Q12846;P | R-HSA-8863973;R-HSA-89515 |
| **R-HSA-1236975** | Antigen processing-Cross presentation | 44 | 195 | 8 | 195 | 0.013664073996216103 | 7.771561172376096E-15 | 2.76667577736589E-12 | 12 | 23 | 0.0016940413935331812 | 9606 | Homo sapiens | PDIA3;PSMA6;PSMA1;PSME1;H | P30501;P30447;P305 | STX7;PDIA3;APP;IDH1;TUBB;HLA-A;HS | O15533;Q12846;P | R-HSA-8863973;R-HSA-89515 |
| **R-HSA-8957275** | Post-translational protein phosphorylation | 24 | 109 | 0 | 0 | 0.007637867003013103 | 2.7533531010703882E-14 | 8.149925179168349E-12 | 1 | 1 | 7.365397363187744E-5 | 9606 | Homo sapiens | APP;SPARC;ITIH2;CSF1;IGFBP5;AHSG;PRKCSH;IGFBP4;IGFBP3;FN1;TNC;LAMC1;KTN1;CST3;C3;IL6;RCN1;LGA | | | | R-HSA-8952289 |
| **R-HSA-909733** | Interferon alpha/beta signaling | 37 | 186 | 8 | 215 | 0.013033424427159974 | 2.7223923115826665E-10 | 6.914876471419973E-8 | 4 | 22 | 0.0016203874199013037 | 9606 | Homo sapiens | HLA-C;HLA-A | P30501;P30447;P305 | APP;LGALS3BP;VCP;CRK;PLOD3;FLNB | O14879;Q9UHH9; | R-HSA-997311;R-HSA-997309 |
| **R-HSA-114608** | Platelet degranulation | 24 | 141 | 4 | 45 | 0.009880176581879336 | 1.2604854937592336E-9 | 2.7982777961454985E-7 | 6 | 11 | 8.101937099506518E-4 | 9606 | Homo sapiens | CAP1;LGALS3BP;APP;ECM1;SP | P55145;Q08380;P629 | APP;VIM;COL1A1;UBQLN1 | O43852;P11021 | R-HSA-482775;R-HSA-482772 |
| **R-HSA-877300** | Interferon gamma signaling | 39 | 250 | 11 | 230 | 0.01751804358489244 | 3.4404598059367686E-9 | 6.777705817695434E-7 | 3 | 16 | 0.001178463578110039 | 9606 | Homo sapiens | HLA-C;HLA-A;B2M | P30501;P30447;P305 | B2M;LMNA;FLNA;LMNA;HLA-C;HLA-A; | O75925;P63165;P | R-HSA-1031716;R-HSA-87728 |
| **R-HSA-381426** | Regulation of Insulin-like Growth Factor (IGF) transport and uptake by Insulin-like Growth Factor Binding Proteins (IGFBPs) | 26 | 127 | 6 | 134 | 0.00889916614112536 | 3.8443372596397296E-8 | 6.842920322158719E-6 | 12 | 14 | 0.0010311556308462842 | 9606 | Homo sapiens | APP;SPARC;ITIH2;CSF1;PRKCS | P09603;P14314;P110 | UBQLN1;YWHAG;SDCBP;EFEMP2;GSN | P24592;P22692;P | R-HSA-381461;R-HSA-381543 |
| **R-HSA-70263** | Gluconeogenesis | 14 | 66 | 0 | 15 | 0.004624763506411604 | 1.2370846946829772E-7 | 1.9917063584395933E-5 | 10 | 26 | 0.0019150033144288135 | 9606 | Homo sapiens | GPI;TPI1;MDH1;MDH2;PGAM1;GOT2;PGK1;ALDOC;ENO1;ALDOA;ENO2;GAPDH | | |  | R-HSA-70481;R-HSA-70482;R |
| **R-HSA-76005** | Response to elevated platelet cytosolic Ca2+ | 24 | 148 | 5 | 105 | 0.010370681802256324 | 3.23271968971639E-7 | 4.7844251407802574E-5 | 7 | 14 | 0.0010311556308462842 | 9606 | Homo sapiens | CAP1;LGALS3BP;APP;ECM1;SP | P55145;Q08380;P629 | STX7;APP;VIM;COL1A1;UBQLN1 | O43852;Q12846;P | R-HSA-482775;R-HSA-482772 |
| **R-HSA-1442490** | Collagen degradation | 13 | 69 | 1 | 12 | 0.004834980029430313 | 5.965813283381749E-7 | 8.113506065399179E-5 | 20 | 34 | 0.002504235103483833 | 9606 | Homo sapiens | COL1A1;COL4A2;MMP1;CTSK;C | P02452;P07339;P121 | APP | P09958 | R-HSA-2471621;R-HSA-14741 |
| **R-HSA-983169** | Class I MHC mediated antigen processing & presentation | 45 | 474 | 12 | 269 | 0.033214210636956065 | 8.257208168327423E-7 | 1.0486654373775828E-4 | 31 | 48 | 0.003535390734330117 | 9606 | Homo sapiens | PDIA3;PSMA6;PSMA1;HSPA5;P | P30501;P30447;P305 | B2M;STX7;PDIA3;APP;IDH1;HLA-C;TUB | O15533;P30101;P |  |
| **R-HSA-2022090** | Assembly of collagen fibrils and other multimeric structures | 12 | 67 | 0 | 14 | 0.004694835680751174 | 3.604124217404525E-6 | 4.2528665765373397E-4 | 20 | 26 | 0.0019150033144288135 | 9606 | Homo sapiens | COL1A1;LOX;COL4A2;MMP1;COL6A2;COL6A1;MMP3;COL6A3;PCOLCE;CTSS;CTSB;PLEC | | |  | R-HSA-2395302;R-HSA-20221 |
| **R-HSA-3000178** | ECM proteoglycans | 15 | 79 | 5 | 50 | 0.005535701772826011 | 4.369261608183628E-6 | 4.849880385083827E-4 | 17 | 23 | 0.0016940413935331812 | 9606 | Homo sapiens | APP;SPARC;LUM;SERPINE1;TN | P02452;P12109;Q141 | UBQLN1;FN1;UBQLN1;DAG1;GRN | Q14118;P05121;P | R-HSA-2467716;R-HSA-40862 |
| **R-HSA-1474228** | Degradation of the extracellular matrix | 20 | 148 | 10 | 89 | 0.010370681802256324 | 9.295664496478118E-6 | 9.648643929756062E-4 | 71 | 105 | 0.007733667231347131 | 9606 | Homo sapiens | CAST;MMP1;MMP2;MMP3;FN1; | P02452;P07339;P121 | APP;MMP2;LGALS3;APP;FN1;MSN;TIM | P09958;P16070;P |  |
| **R-HSA-3371568** | Attenuation phase | 9 | 47 | 0 | 0 | 0.0032933921939597784 | 9.845555030363329E-6 | 9.648643929756062E-4 | 3 | 5 | 3.682698681593872E-4 | 9606 | Homo sapiens | HSPA8;HSPB1;HSPA1A |  |  |  | R-HSA-3371554;R-HSA-50823 |
| **R-HSA-70326** | Glucose metabolism | 16 | 140 | 0 | 32 | 0.009810104407539766 | 3.1930061813234545E-5 | 0.0029694957486308127 | 18 | 50 | 0.003682698681593872 | 9606 | Homo sapiens | GPI;TPI1;MDH1;MDH2;PGAM1;GOT2;ENO1;ENO2;PKM;PGK1;ALDOC;ALDOA;GAPDH | | |  | R-HSA-71495;R-HSA-70471;R |
| **R-HSA-198933** | Immunoregulatory interactions between a Lymphoid and a non-Lymphoid cell | 38 | 316 | 29 | 361 | 0.022142807091304045 | 3.745459848580879E-5 | 0.003333459265236982 | 18 | 44 | 0.0032407748398026073 | 9606 | Homo sapiens | COL1A1;C3;HLA-C;HLA-A;B2M | P02452;P30501;P304 | ENO1;MDH2;STIP1;NPM1;EMILIN1;YW | P19320;P12830;Q | R-HSA-199043;R-HSA-199587 |
| **R-HSA-3371571** | HSF1-dependent transactivation | 9 | 59 | 0 | 0 | 0.004134258286034615 | 5.6743789506041864E-5 | 0.004766478318507517 | 4 | 8 | 5.892317890550195E-4 | 9606 | Homo sapiens | HSPA8;HSPB1;HSPA1A |  |  |  | R-HSA-3371554;R-HSA-50823 |
| **R-HSA-70171** | Glycolysis | 13 | 110 | 0 | 17 | 0.007707939177352673 | 6.212573651687148E-5 | 0.004970058921349718 | 8 | 24 | 0.0017676953671650585 | 9606 | Homo sapiens | GPI;PKM;TPI1;PGAM1;PGK1;ALDOC;ENO1;ALDOA;ENO2;GAPDH | | |  | R-HSA-70449;R-HSA-70471;R |
| **R-HSA-437239** | Recycling pathway of L1 | 12 | 55 | 2 | 74 | 0.0038539695886763365 | 2.4290663459691775E-4 | 0.018703810863962667 | 14 | 14 | 0.0010311556308462842 | 9606 | Homo sapiens | TUBA1C;TUBB;DPYSL2;RDX;MS | P15311;P63261;Q3ZC | APP;PSMA1 | P28482 | R-HSA-443783;R-HSA-443779 |
| **R-HSA-6802948** | Signaling by high-kinase activity BRAF mutants | 7 | 44 | 0 | 0 | 0.0030831756709410693 | 2.918123596865607E-4 | 0.021594114616805493 | 4 | 6 | 4.419238417912646E-4 | 9606 | Homo sapiens | YWHAB;FN1;PEBP1;TLN1;VCL;ACTB;ACTG1 | |  |  | R-HSA-6802912;R-HSA-68029 |
| **R-HSA-5674135** | MAP2K and MAPK activation | 7 | 49 | 0 | 0 | 0.0034335365426389182 | 5.501993785832493E-4 | 0.0390641558794107 | 8 | 12 | 8.838476835825292E-4 | 9606 | Homo sapiens | YWHAB;FN1;PEBP1;TLN1;VCL;ACTB;ACTG1 | |  |  | R-HSA-9657603;R-HSA-56729 |
| **R-HSA-3371497** | HSP90 chaperone cycle for steroid hormone receptors (SHR) in the presence of ligand | 11 | 72 | 2 | 50 | 0.005045196552449022 | 6.301361660903115E-4 | 0.042849259294141184 | 11 | 16 | 0.001178463578110039 | 9606 | Homo sapiens | CAP1;TUBA1C;STIP1;HSPA8;DY | P11142;P0DMV8;P0D | HSPB1;S100A6 | Q02790 | R-HSA-5618085;R-HSA-56180 |

REACTOME 60D

| **Pathway identifier** | **Pathway name** | **#Entities found** | **#Entities total** | **#Interactors found** | **#Interactors total** | **Entities ratio** | **Entities pValue** | **Entities FDR** | **#Reactions found** | **#Reactions total** | **Reactions ratio** | **Species identifier** | **Species name** | **Submitted entities found** | **Mapped entities** | **Submitted entities hit interactor** | **Interacts with** | **Found reaction identifiers** |
| --- | --- | --- | --- | --- | --- | --- | --- | --- | --- | --- | --- | --- | --- | --- | --- | --- | --- | --- |
| **R-HSA-1236977** | Endosomal/Vacuolar pathway | 36 | 82 | 0 | 0 | 0.00574591829584472 | 1.1102230246251565E-16 | 5.917488721252084E-14 | 4 | 4 | 2.9461589452750976E-4 | 9606 | Homo sapiens | HLA-C;HLA-A;CTSS |  |  |  | R-HSA-1236948;R-HSA-12369 |
| **R-HSA-6798695** | Neutrophil degranulation | 47 | 480 | 0 | 0 | 0.03363464368299348 | 1.1102230246251565E-16 | 5.917488721252084E-14 | 10 | 10 | 7.365397363187744E-4 | 9606 | Homo sapiens | GPI;CSTB;GRN;GSTP1;HEXB;PXN;CTSS;CST3;HEBP2;LGALS3;SDCBP;ANPEP;TIMP2;TOM1;STOM;QSOX1;CTSD | | | | R-HSA-6798743;R-HSA-67993 |
| **R-HSA-983170** | Antigen Presentation: Folding, assembly and peptide loading of class I MHC | 37 | 103 | 8 | 164 | 0.007217433956975685 | 1.1102230246251565E-16 | 5.917488721252084E-14 | 13 | 16 | 0.001178463578110039 | 9606 | Homo sapiens | PDIA3;HSPA5;HLA-C;HLA-A | P30501;P30447;P305 | PDIA3;IDH1;HLA-C;COL1A1;HLA-A;HLA | O15533;P30101;P | R-HSA-983148;R-HSA-895149 |
| **R-HSA-1236974** | ER-Phagosome pathway | 37 | 173 | 7 | 195 | 0.012122486160745568 | 4.285460875053104E-14 | 1.673328142715036E-11 | 6 | 10 | 7.365397363187744E-4 | 9606 | Homo sapiens | PDIA3;HLA-C;HLA-A;SEC22B | P30501;P30447;P305 | STX7;PDIA3;IDH1;SEC22B;HLA-A;HSPB | O15533;Q12846;P | R-HSA-8863973;R-HSA-89515 |
| **R-HSA-1236975** | Antigen processing-Cross presentation | 38 | 195 | 7 | 195 | 0.013664073996216103 | 5.229150445984487E-14 | 1.673328142715036E-11 | 10 | 23 | 0.0016940413935331812 | 9606 | Homo sapiens | PDIA3;HLA-C;HLA-A;SEC22B;CT | P30501;P30447;P305 | STX7;PDIA3;IDH1;SEC22B;HLA-A;HSPB | O15533;Q12846;P | R-HSA-8863973;R-HSA-89515 |
| **R-HSA-909733** | Interferon alpha/beta signaling | 37 | 186 | 6 | 215 | 0.013033424427159974 | 4.910516437917067E-13 | 1.30619737248594E-10 | 4 | 22 | 0.0016203874199013037 | 9606 | Homo sapiens | HLA-C;HLA-A | P30501;P30447;P305 | PXN;TPM3;CRK;CAT;EEF1A1;TPM3 | P18031;O14879;Q | R-HSA-997311;R-HSA-997309 |
| **R-HSA-877300** | Interferon gamma signaling | 37 | 250 | 11 | 230 | 0.01751804358489244 | 9.089973218578962E-11 | 2.0725138938360033E-8 | 3 | 16 | 0.001178463578110039 | 9606 | Homo sapiens | HLA-C;HLA-A | P30501;P30447;P305 | LMNA;FLNA;LMNA;HLA-C;HLA-A;ALB;N | O75925;P63165;P | R-HSA-1031716;R-HSA-87728 |
| **R-HSA-983169** | Class I MHC mediated antigen processing & presentation | 42 | 474 | 10 | 269 | 0.033214210636956065 | 3.38588324044764E-8 | 6.7717664808952804E-6 | 28 | 48 | 0.003535390734330117 | 9606 | Homo sapiens | PDIA3;HSPA5;TOM1;HLA-C;UBE | P30501;P30447;P305 | STX7;PDIA3;IDH1;HLA-C;COL1A1;HLA- | O15533;P30101;P | R-HSA-8951499;R-HSA-12369 |
| **R-HSA-70263** | Gluconeogenesis | 13 | 66 | 1 | 15 | 0.004624763506411604 | 6.592928170068291E-8 | 1.1669482861020875E-5 | 11 | 26 | 0.0019150033144288135 | 9606 | Homo sapiens | GPI;TPI1;MDH1;MDH2;PGAM1;G | P06733;P06744;P072 | HSPD1 | Q16822 | R-HSA-70481;R-HSA-70482;R |
| **R-HSA-198933** | Immunoregulatory interactions between a Lymphoid and a non-Lymphoid cell | 37 | 316 | 23 | 361 | 0.022142807091304045 | 6.295471896011762E-7 | 1.0072755033618819E-4 | 12 | 44 | 0.0032407748398026073 | 9606 | Homo sapiens | COL1A1;COL1A2;HLA-C;HLA-A | P02452;P30501;P304 | ANXA2;UBQLN1;FSCN1;ZYX;ENO1;MD | P19320;P12830;Q | R-HSA-199043;R-HSA-199587 |
| **R-HSA-3371568** | Attenuation phase | 9 | 47 | 0 | 0 | 0.0032933921939597784 | 1.6915170131248658E-6 | 2.4526996690310554E-4 | 3 | 5 | 3.682698681593872E-4 | 9606 | Homo sapiens | HSPA8;HSPB1;HSPA1A |  |  |  | R-HSA-3371554;R-HSA-50823 |
| **R-HSA-70326** | Glucose metabolism | 15 | 140 | 1 | 32 | 0.009810104407539766 | 9.421381282681551E-6 | 0.0011781235065537743 | 19 | 50 | 0.003682698681593872 | 9606 | Homo sapiens | GPI;PKM;TPI1;MDH1;MDH2;PGA | P06744;P06733;P072 | HSPD1 | Q16822 | R-HSA-71495;R-HSA-70471;R |
| **R-HSA-8957275** | Post-translational protein phosphorylation | 12 | 109 | 0 | 0 | 0.007637867003013103 | 9.739868583991118E-6 | 0.0011781235065537743 | 1 | 1 | 7.365397363187744E-5 | 9606 | Homo sapiens | CST3;RCN1;ITIH2;LGALS1;IGFBP5;AHSG;PRKCSH;IGFBP4;ALB;IGFBP7;QSOX1;PDIA6 | | |  | R-HSA-8952289 |
| **R-HSA-3371571** | HSF1-dependent transactivation | 9 | 59 | 0 | 0 | 0.004134258286034615 | 1.0334416724155915E-5 | 0.0011781235065537743 | 4 | 8 | 5.892317890550195E-4 | 9606 | Homo sapiens | HSPA8;HSPB1;HSPA1A |  |  |  | R-HSA-3371554;R-HSA-50823 |
| **R-HSA-70171** | Glycolysis | 12 | 110 | 0 | 17 | 0.007707939177352673 | 3.1358327504005246E-5 | 0.003323982715424556 | 8 | 24 | 0.0017676953671650585 | 9606 | Homo sapiens | GPI;PKM;TPI1;PGAM1;PGK1;ALDOC;ENO1;ALDOA;GAPDH | | |  | R-HSA-70449;R-HSA-70471;R |
| **R-HSA-114608** | Platelet degranulation | 14 | 141 | 5 | 45 | 0.009880176581879336 | 1.0641406022215971E-4 | 0.010641406022215971 | 7 | 11 | 8.101937099506518E-4 | 9606 | Homo sapiens | ECM1;HSPA5;AHSG;SERPINE1; | P08758;Q96C24;P629 | VIM;COL1A1;PDIA6;UBE2V1;UBQLN1 | O43852;P11021 | R-HSA-482775;R-HSA-482772 |
| **R-HSA-1442490** | Collagen degradation | 8 | 69 | 0 | 12 | 0.004834980029430313 | 5.055598282120988E-4 | 0.047522623851937285 | 18 | 34 | 0.002504235103483833 | 9606 | Homo sapiens | COL1A1;COL1A2;MMP1;MMP2;MMP3;CTSD | |  |  | R-HSA-2471621;R-HSA-14741 |
| **R-HSA-196025** | Formation of annular gap junctions | 4 | 11 | 0 | 7 | 7.707939177352673E-4 | 8.097172742060899E-4 | 0.07125512013013591 | 2 | 2 | 1.4730794726375488E-4 | 9606 | Homo sapiens | CLTB;CLTA;ACTB;ACTG1 |  |  |  | R-HSA-196017;R-HSA-196026 |

**Venn Analysis**

| **Names** | **total** | **elements** |
| --- | --- | --- |
| **C10 IR10 IR30 IR60** | 8 | Collagen degradation |
|  |  | Attenuation phase |
|  |  | HSF1-dependent transactivation |
|  |  | Post-translational protein phosphorylation |
|  |  | Neutrophil degranulation |
|  |  | Platelet degranulation |
|  |  | Gluconeogenesis |
|  |  | Glycolysis |
| **C10 IR10 IR30** | 5 | ECM proteoglycans |
|  |  | Regulation of Insulin-like Growth Factor (IGF) transport and uptake by Insulin-like Growth Factor Binding Proteins (IGFBPs) |
|  |  | Response to elevated platelet cytosolic Ca2+ |
|  |  | Assembly of collagen fibrils and other multimeric structures |
|  |  | Degradation of the extracellular matrix |
| **IR10 IR30 IR60** | 1 | Glucose metabolism |
| **C10 IR10** | 16 | RHOBTB GTPase Cycle |
|  |  | A tetrasaccharide linker sequence is required for GAG synthesis |
|  |  | Sensory processing of sound by outer hair cells of the cochlea |
|  |  | HSF1 activation |
|  |  | Collagen formation |
|  |  | Interleukin-4 and Interleukin-13 signaling |
|  |  | Defective B3GALT6 causes EDSP2 and SEMDJL1 |
|  |  | Collagen chain trimerization |
|  |  | Crosslinking of collagen fibrils |
|  |  | Defective B3GAT3 causes JDSSDHD |
|  |  | Laminin interactions |
|  |  | Activation of Matrix Metalloproteinases |
|  |  | RHOBTB2 GTPase cycle |
|  |  | Diseases associated with glycosaminoglycan metabolism |
|  |  | Collagen biosynthesis and modifying enzymes |
|  |  | Defective B4GALT7 causes EDS, progeroid type |
| **C10 IR30** | 2 | MAP2K and MAPK activation |
|  |  | Signaling by high-kinase activity BRAF mutants |
| **IR10 IR30** | 2 | HSP90 chaperone cycle for steroid hormone receptors (SHR) in the presence of ligand |

|  | | Recycling pathway of L1 |
| --- | --- | --- |
| **IR30 IR60** | 8 | ER-Phagosome pathway |
|  |  | Antigen processing-Cross presentation |
|  |  | Immunoregulatory interactions between a Lymphoid and a non-Lymphoid cell |
|  |  | Interferon gamma signaling |
|  |  | Class I MHC mediated antigen processing & presentation |
|  |  | Endosomal/Vacuolar pathway |
|  |  | Antigen Presentation: Folding, assembly and peptide loading of class I MHC |
|  |  | Interferon alpha/beta signaling |
| **C10** | 19 | Signaling by RAF1 mutants |
|  |  | Paradoxical activation of RAF signaling by kinase inactive BRAF |
|  |  | Extracellular matrix organization |
|  |  | Signaling downstream of RAS mutants |
|  |  | Platelet activation, signaling and aggregation |
|  |  | NCAM1 interactions |
|  |  | MET promotes cell motility |
|  |  | MET activates PTK2 signaling |
|  |  | Signaling by moderate kinase activity BRAF mutants |
|  |  | Signaling by BRAF and RAF1 fusions |
|  |  | Defective EXT2 causes exostoses 2 |
|  |  | Elastic fibre formation |
|  |  | Signaling by RAS mutants |
|  |  | Defective EXT1 causes exostoses 1, TRPS2 and CHDS |
|  |  | Molecules associated with elastic fibres |
|  |  | Anchoring fibril formation |
|  |  | Oncogenic MAPK signaling |
|  |  | Non-integrin membrane-ECM interactions |
|  |  | Integrin cell surface interactions |
| **IR10** | 1 | RHO GTPases activate IQGAPs |
