# Supplementary material for: Progression of irradiated mesenchymal stromal cells from early to late senescence: Changes in SASP composition and anti‐tumour properties
Source: Cell Prolif. 2023 Mar 22;56(6):e13401. doi: 10.1111/cpr.13401 (PMC10280137; doi:10.1111/cpr.13401)
Supplement: Supplementary file 4 — Supplementary file S4. Network analysis. The results of Minimum IMEx Interactome Networks are reported. The analysis was performed on secretomes obtained 10, 30 and 60 days (10D, 30D, 60D) post‐x‐ray treatment of MSCs and from unirradiated MSCs (CT). Venn diagram evaluation was performed on nodes having a degree higher than 20 and a betweenness higher than 200. The Venn analysis allowed identification of common and specific nodes among the several experimental conditions. [file CPR-56-e13401-s001.docx]

**Supplementary file 4 – Network analysis**

The results of Minimum IMEx Interactome Networks are reported. The analysis was performed on secretomes obtained 10, 30, and 60 days (10D, 30D, 60D) post-X-ray treatment of MSCs and from unirradiated MSCs (CT). Venn diagram evaluation was performed on nodes having a degree higher than 20 and a betweenness higher than 200. The Venn analysis allowed identification of common and specific nodes among the several experimental conditions.

Network CT Network 10D Network 30D Network 60D Venn Analysis

node_table

| **Id** | **Label** | **Degree** | **Betweenness** | **Expression** | **Id** | **Label** | **Degree** | **Betweenn** | **Expressio** | **Id** | **Label** | **Degree** | **Betweenn** | **Expressio** | **Id** | **Label** | **Degree** | **Betweenn** | **Expressio** |
| --- | --- | --- | --- | --- | --- | --- | --- | --- | --- | --- | --- | --- | --- | --- | --- | --- | --- | --- | --- |
| **7316** | UBC | 100 | 9620.95 | 0 | **7316** | UBC | 257 | 44530.88 | 0 | **7316** | UBC | 265 | 49412.94 | 0 | **7316** | UBC | 225 | 38925.6 | 0 |
| **2335** | FN1 | 92 | 6323 | 0 | **2335** | FN1 | 118 | 5198.82 | 0 | **2335** | FN1 | 157 | 14539.95 | 0 | **2335** | FN1 | 88 | 2675.06 | 0 |
| **7534** | YWHAZ | 69 | 2255.33 | 0 | **7534** | YWHAZ | 116 | 5925.6 | 0 | **351** | APP | 118 | 12358.84 | 0 | **7534** | YWHAZ | 78 | 3214.95 | 0 |
| **351** | APP | 68 | 5027.46 | 0 | **351** | APP | 111 | 11706.83 | 0 | **7534** | YWHAZ | 106 | 5331.06 | 0 | **9768** | KIAA0101 | 76 | 2525.4 | 0 |
| **3320** | HSP90AA1 | 53 | 1775.51 | 0 | **1994** | ELAVL1 | 111 | 11402.56 | 0 | **9768** | KIAA0101 | 100 | 3462.95 | 0 | **1017** | CDK2 | 76 | 1985.29 | 0 |
| **1017** | CDK2 | 48 | 794.39 | 0 | **9768** | KIAA0101 | 97 | 3398.25 | 0 | **1017** | CDK2 | 92 | 2724.08 | 0 | **2099** | ESR1 | 60 | 1243.34 | 0 |
| **3326** | HSP90AB1 | 47 | 1179.04 | 0 | **1017** | CDK2 | 97 | 2607.8 | 0 | **2099** | ESR1 | 80 | 1923.62 | 0 | **351** | APP | 52 | 1292.52 | 0 |
| **9768** | KIAA0101 | 42 | 946.9 | 0 | **2099** | ESR1 | 92 | 2486.25 | 0 | **10971** | YWHAQ | 68 | 2337.95 | 0 | **3312** | HSPA8 | 46 | 1452.34 | 0 |
| **2597** | GAPDH | 40 | 717.81 | 0 | **7412** | VCAM1 | 85 | 1392.96 | 0 | **1994** | ELAVL1 | 66 | 3054.16 | 0 | **1994** | ELAVL1 | 45 | 1702.02 | 0 |
| **60** | ACTB | 38 | 1328.41 | 0 | **3676** | ITGA4 | 82 | 1107.55 | 0 | **3312** | HSPA8 | 63 | 3284.21 | 0 | **2597** | GAPDH | 41 | 860.69 | 0 |
| **3309** | HSPA5 | 37 | 1051.37 | 0 | **3320** | HSP90AA1 | 81 | 3098.93 | 0 | **2597** | GAPDH | 58 | 1392.47 | 0 | **3309** | HSPA5 | 39 | 1024.59 | 0 |
| **2099** | ESR1 | 37 | 337.11 | 0 | **10971** | YWHAQ | 76 | 3063.8 | 0 | **7415** | VCP | 56 | 2181.11 | 0 | **55832** | CAND1 | 39 | 498.79 | 0 |
| **2023** | ENO1 | 33 | 796.12 | 0 | **3326** | HSP90AB | 74 | 2089.85 | 0 | **55832** | CAND1 | 54 | 745 | 0 | **5829** | PXN | 38 | 1049.98 | 0 |
| **7414** | VCL | 33 | 511.31 | 0 | **6613** | SUMO2 | 73 | 1513.69 | 0 | **3309** | HSPA5 | 51 | 1556.47 | 0 | **3172** | HNF4A | 38 | 1036.17 | 0 |
| **1915** | EEF1A1 | 30 | 1215.57 | 0 | **3312** | HSPA8 | 69 | 2075.42 | 0 | **7414** | VCL | 51 | 1326.06 | 0 | **7428** | VHL | 37 | 655.84 | 0 |
| **7532** | YWHAG | 30 | 607.29 | 0 | **3309** | HSPA5 | 65 | 2402.51 | 0 | **7428** | VHL | 50 | 1373.68 | 0 | **1386** | ATF2 | 36 | 568.59 | 0 |
| **2** | A2M | 29 | 1352.34 | 0 | **7415** | VCP | 61 | 2191.48 | 0 | **60** | ACTB | 49 | 2523.79 | 0 | **7414** | VCL | 36 | 515.15 | 0 |
| **3303** | HSPA1A | 29 | 632.86 | 0 | **60** | ACTB | 58 | 2763.31 | 0 | **2885** | GRB2 | 47 | 1066.45 | 0 | **2885** | GRB2 | 35 | 682.4 | 0 |
| **7431** | VIM | 27 | 565.52 | 0 | **2597** | GAPDH | 58 | 1455.54 | 0 | **1915** | EEF1A1 | 46 | 2255.42 | 0 | **1915** | EEF1A1 | 33 | 1251.07 | 0 |
| **5315** | PKM | 27 | 432.99 | 0 | **9924** | PAN2 | 57 | 1021.3 | 0 | **3172** | HNF4A | 43 | 1231.18 | 0 | **1956** | EGFR | 33 | 493.37 | 0 |
| **1386** | ATF2 | 27 | 320.92 | 0 | **7414** | VCL | 53 | 936.81 | 0 | **4904** | YBX1 | 43 | 1090.35 | 0 | **60** | ACTB | 30 | 1220.81 | 0 |
| **10971** | YWHAQ | 27 | 148.71 | 0 | **2885** | GRB2 | 52 | 1541.31 | 0 | **1386** | ATF2 | 43 | 667.19 | 0 | **3178** | HNRNPA1 | 30 | 872.56 | 0 |
| **2885** | GRB2 | 24 | 315.87 | 0 | **7428** | VHL | 52 | 1263.85 | 0 | **7532** | YWHAG | 41 | 1337.23 | 0 | **3329** | HSPD1 | 30 | 438.82 | 0 |
| **1994** | ELAVL1 | 23 | 441.73 | 0 | **55832** | CAND1 | 52 | 711.73 | 0 | **3190** | HNRNPK | 41 | 1155.07 | 0 | **3303** | HSPA1A | 28 | 1264.84 | 0 |
| **3939** | LDHA | 23 | 303.05 | 0 | **3178** | HNRNPA1 | 51 | 1703.3 | 0 | **1956** | EGFR | 39 | 687.06 | 0 | **4869** | NPM1 | 28 | 901.82 | 0 |
| **3014** | H2AFX | 23 | 246.36 | 0 | **7431** | VIM | 46 | 1438.68 | 0 | **3303** | HSPA1A | 38 | 1703 | 0 | **7013** | TERF1 | 28 | 315.89 | 0 |
| **5052** | PRDX1 | 23 | 156.28 | 0 | **3329** | HSPD1 | 46 | 1117.32 | 0 | **9657** | IQCB1 | 38 | 702.61 | 0 | **1398** | CRK | 27 | 921.94 | 0 |
| **2923** | PDIA3 | 21 | 531.25 | 0 | **1386** | ATF2 | 46 | 739.42 | 0 | **5829** | PXN | 37 | 806.33 | 0 | **7529** | YWHAB | 27 | 611.29 | 0 |
| **6667** | SP1 | 21 | 434.5 | 0 | **3303** | HSPA1A | 45 | 1325.59 | 0 | **7431** | VIM | 36 | 899.42 | 0 | **7431** | VIM | 26 | 720.13 | 0 |
| **5829** | PXN | 21 | 359.59 | 0 | **3308** | HSPA4 | 44 | 1404.41 | 0 | **203068** | TUBB | 36 | 819.85 | 0 | **5093** | PCBP1 | 26 | 407.34 | 0 |
| **226** | ALDOA | 21 | 169.28 | 0 | **9657** | IQCB1 | 43 | 1034.13 | 0 | **2023** | ENO1 | 35 | 1441.05 | 0 | **4609** | MYC | 26 | 395.39 | 0 |
| **213** | ALB | 20 | 1318.84 | 0 | **2023** | ENO1 | 41 | 1170 | 0 | **7529** | YWHAB | 35 | 804.82 | 0 | **4627** | MYH9 | 25 | 805.55 | 0 |
| **7428** | VHL | 20 | 304.44 | 0 | **3190** | HNRNPK | 41 | 1041.8 | 0 | **3184** | HNRNPD | 34 | 801.52 | 0 | **2697** | GJA1 | 25 | 313.18 | 0 |
| **3945** | LDHB | 20 | 283.26 | 0 | **3106** | HLA-B | 41 | 523.21 | 0 | **5315** | PKM | 34 | 567.68 | 0 | **3320** | HSP90AA1 | 25 | 210.35 | 0 |
| **1072** | CFL1 | 20 | 217.07 | 0 | **4869** | NPM1 | 40 | 1482.28 | 0 | **4869** | NPM1 | 33 | 1744.89 | 0 | **2023** | ENO1 | 24 | 512.85 | 0 |
| **2697** | GJA1 | 20 | 215.51 | 0 | **4904** | YBX1 | 40 | 663.62 | 0 | **4609** | MYC | 33 | 692.58 | 0 | **5052** | PRDX1 | 24 | 384.03 | 0 |
| **4627** | MYH9 | 20 | 201.87 | 0 | **7532** | YWHAG | 39 | 906.2 | 0 | **2316** | FLNA | 33 | 667.65 | 0 | **25913** | POT1 | 24 | 313.46 | 0 |
| **7057** | THBS1 | 19 | 972.16 | 0 | **2316** | FLNA | 39 | 816.79 | 0 | **1398** | CRK | 33 | 585.58 | 0 | **3939** | LDHA | 24 | 254.62 | 0 |
| **7094** | TLN1 | 19 | 215.27 | 0 | **3181** | HNRNPA2 | 39 | 600.75 | 0 | **2697** | GJA1 | 33 | 508.98 | 0 | **5684** | PSMA3 | 24 | 224.67 | 0 |
| **302** | ANXA2 | 19 | 210.23 | 0 | **1956** | EGFR | 39 | 576.5 | 0 | **3181** | HNRNPA2 | 33 | 479.49 | 0 | **5315** | PKM | 24 | 164.34 | 0 |
| **4904** | YBX1 | 19 | 154.98 | 0 | **3172** | HNF4A | 38 | 1481.02 | 0 | **3320** | HSP90AA1 | 33 | 403.47 | 0 | **3958** | LGALS3 | 23 | 1134.75 | 0 |
| **1956** | EGFR | 19 | 127.15 | 0 | **3184** | HNRNPD | 38 | 560.05 | 0 | **8450** | CUL4B | 33 | 365.43 | 0 | **6667** | SP1 | 23 | 291.62 | 0 |
| **4653** | MYOC | 18 | 589.25 | 0 | **2697** | GJA1 | 38 | 556.82 | 0 | **7157** | TP53 | 32 | 639.07 | 0 | **2130** | EWSR1 | 22 | 1040.9 | 0 |
| **3181** | HNRNPA2B1 | 18 | 223.02 | 0 | **4609** | MYC | 37 | 706.86 | 0 | **5684** | PSMA3 | 32 | 286.2 | 0 | **3159** | HMGA1 | 22 | 590.12 | 0 |
| **55832** | CAND1 | 18 | 115.13 | 0 | **5315** | PKM | 37 | 648.16 | 0 | **3939** | LDHA | 31 | 460.69 | 0 | **2316** | FLNA | 22 | 339.39 | 0 |
| **207** | AKT1 | 18 | 109.09 | 0 | **3921** | RPSA | 37 | 605.98 | 0 | **26986** | PABPC1 | 31 | 320.26 | 0 | **226** | ALDOA | 22 | 318.99 | 0 |
| **4313** | MMP2 | 17 | 477.84 | 0 | **7157** | TP53 | 36 | 943.09 | 0 | **25913** | POT1 | 30 | 395.09 | 0 | **7157** | TP53 | 22 | 313.04 | 0 |
| **71** | ACTG1 | 17 | 381.09 | 0 | **5684** | PSMA3 | 36 | 367.1 | 0 | **207** | AKT1 | 30 | 346.9 | 0 | **5725** | PTBP1 | 22 | 191.01 | 0 |
| **7533** | YWHAH | 17 | 281.04 | 0 | **4670** | HNRNPM | 35 | 451.64 | 0 | **3958** | LGALS3 | 29 | 1529.88 | 0 | **3956** | LGALS1 | 21 | 636.85 | 0 |
| **5478** | PPIA | 17 | 144.48 | 0 | **4841** | NONO | 34 | 1174.76 | 0 | **5052** | PRDX1 | 29 | 961.89 | 0 | **302** | ANXA2 | 21 | 378.28 | 0 |
| **3172** | HNF4A | 16 | 480.52 | 0 | **5829** | PXN | 34 | 710.07 | 0 | **3313** | HSPA9 | 29 | 358.38 | 0 | **3945** | LDHB | 20 | 409.63 | 0 |
| **1277** | COL1A1 | 16 | 427.31 | 0 | **5052** | PRDX1 | 34 | 519.89 | 0 | **2130** | EWSR1 | 28 | 1348.41 | 0 | **1499** | CTNNB1 | 20 | 380.23 | 0 |
| **7430** | EZR | 16 | 399.28 | 0 | **3939** | LDHA | 34 | 390.15 | 0 | **6667** | SP1 | 28 | 669.17 | 0 | **867** | CBL | 20 | 202.13 | 0 |
| **8450** | CUL4B | 16 | 135.59 | 0 | **8450** | CUL4B | 34 | 362.1 | 0 | **4841** | NONO | 27 | 937.31 | 0 | **207** | AKT1 | 20 | 178.12 | 0 |
| **4000** | LMNA | 16 | 122.3 | 0 | **6667** | SP1 | 33 | 881.3 | 0 | **5093** | PCBP1 | 27 | 283.22 | 0 | **4313** | MMP2 | 19 | 932.54 | 0 |
| **5684** | PSMA3 | 16 | 113.03 | 0 | **10627** | MYL12A | 33 | 334.01 | 0 | **5725** | PTBP1 | 27 | 184.51 | 0 | **3181** | HNRNPA2 | 19 | 500.46 | 0 |
| **6927** | HNF1A | 15 | 283.69 | 0 | **1938** | EEF2 | 33 | 314.35 | 0 | **4627** | MYH9 | 26 | 659.3 | 0 | **3476** | IGBP1 | 19 | 219.73 | 0 |
| **6678** | SPARC | 15 | 277.01 | 0 | **3958** | LGALS3 | 32 | 2171.56 | 0 | **3159** | HMGA1 | 26 | 553.21 | 0 | **7167** | TPI1 | 19 | 108.12 | 0 |
| **87** | ACTN1 | 15 | 170.82 | 0 | **302** | ANXA2 | 31 | 642.77 | 0 | **3945** | LDHB | 26 | 465.68 | 0 | **2896** | GRN | 18 | 989.1 | 0 |
| **81** | ACTN4 | 15 | 114.1 | 0 | **4627** | MYH9 | 31 | 607.45 | 0 | **7094** | TLN1 | 26 | 359.39 | 0 | **7791** | ZYX | 18 | 386.67 | 0 |
| **25913** | POT1 | 15 | 99.46 | 0 | **7430** | EZR | 30 | 1280.28 | 0 | **506** | ATP5B | 26 | 351.32 | 0 | **6927** | HNF1A | 18 | 327.8 | 0 |
| **5230** | PGK 1,00 | 15 | 44.51 | 0 | **1499** | CTNNB1 | 30 | 1152.11 | 0 | **6709** | SPTAN1 | 25 | 490.35 | 0 | **2923** | PDIA3 | 18 | 257.39 | 0 |
| **2896** | GRN | 14 | 463.4 | 0 | **207** | AKT1 | 30 | 255.32 | 0 | **1072** | CFL1 | 25 | 396.07 | 0 | **25824** | PRDX5 | 17 | 698.97 | 0 |
| **2318** | FLNC | 14 | 433.23 | 0 | **51377** | UCHL5 | 30 | 193.5 | 0 | **71** | ACTG1 | 24 | 924.47 | 0 | **10174** | SORBS3 | 17 | 534.32 | 0 |
| **7157** | TP53 | 14 | 355.38 | 0 | **226** | ALDOA | 29 | 412.24 | 0 | **2923** | PDIA3 | 24 | 789.08 | 0 | **30011** | SH3KBP1 | 17 | 423.9 | 0 |
| **4924** | NUCB1 | 14 | 148.18 | 0 | **1072** | CFL1 | 29 | 353.46 | 0 | **302** | ANXA2 | 24 | 374.16 | 0 | **3315** | HSPB1 | 17 | 290.56 | 0 |
| **5216** | PFN1 | 14 | 49.81 | 0 | **2923** | PDIA3 | 28 | 1156.68 | 0 | **226** | ALDOA | 24 | 300.33 | 0 | **5110** | PCMT1 | 16 | 593.35 | 0 |
| **7170** | TPM3 | 13 | 86.51 | 0 | **7184** | HSP90B1 | 28 | 564.83 | 0 | **3178** | HNRNPA1 | 24 | 256.94 | 0 | **6227** | RPS21 | 16 | 304.4 | 0 |
| **4609** | MYC | 13 | 65.87 | 0 | **3945** | LDHB | 28 | 414.98 | 0 | **3956** | LGALS1 | 23 | 813.02 | 0 | **27316** | RBMX | 16 | 279.78 | 0 |
| **2317** | FLNB | 12 | 385.21 | 0 | **71** | ACTG1 | 27 | 833.63 | 0 | **30008** | EFEMP2 | 23 | 694.38 | 0 | **328** | APEX1 | 16 | 243.11 | 0 |
| **2192** | FBLN1 | 12 | 366.5 | 0 | **5034** | P4HB | 27 | 523.85 | 0 | **5478** | PPIA | 23 | 280.2 | 0 | **5062** | PAK2 | 16 | 162.24 | 0 |
| **4478** | MSN | 12 | 275.44 | 0 | **6709** | SPTAN1 | 27 | 411.81 | 0 | **5034** | P4HB | 23 | 255.59 | 0 | **5478** | PPIA | 16 | 138.66 | 0 |
| **5479** | PPIB | 12 | 264.42 | 0 | **7094** | TLN1 | 27 | 302.8 | 0 | **7167** | TPI1 | 23 | 185.24 | 0 | **2521** | FUS | 16 | 89.51 | 0 |
| **9601** | PDIA4 | 12 | 208.25 | 0 | **5683** | PSMA2 | 27 | 164.6 | 0 | **10383** | TUBB4B | 23 | 114.15 | 0 | **7001** | PRDX2 | 16 | 72.45 | 0 |
| **1499** | CTNNB1 | 12 | 190.17 | 0 | **213** | ALB | 26 | 2031.67 | 0 | **4313** | MMP2 | 22 | 1009 | 0 | **213** | ALB | 15 | 671.19 | 0 |
| **197** | AHSG | 12 | 156.9 | 0 | **3956** | LGALS1 | 26 | 725.84 | 0 | **7430** | EZR | 22 | 942.75 | 0 | **197** | AHSG | 15 | 421.89 | 0 |
| **3336** | HSPE1 | 12 | 111.17 | 0 | **5686** | PSMA5 | 26 | 457.25 | 0 | **567** | B2M | 22 | 530.1 | 0 | **5887** | RAD23B | 15 | 383.22 | 0 |
| **8407** | TAGLN2 | 12 | 96.35 | 0 | **506** | ATP5B | 26 | 391.66 | 0 | **1499** | CTNNB1 | 22 | 438.13 | 0 | **10949** | HNRNPA0 | 15 | 368.56 | 0 |
| **301** | ANXA1 | 12 | 38.19 | 0 | **5688** | PSMA7 | 26 | 281.46 | 0 | **10146** | G3BP1 | 22 | 317.2 | 0 | **6206** | RPS12 | 15 | 343.27 | 0 |
| **3486** | IGFBP3 | 11 | 540.19 | 0 | **4736** | RPL10A | 26 | 250.93 | 0 | **672** | BRCA1 | 22 | 184.85 | 0 | **1080** | CFTR | 15 | 141.16 | 0 |
| **5054** | SERPINE1 | 11 | 433.18 | 0 | **10289** | EIF1B | 26 | 205.74 | 0 | **2896** | GRN | 21 | 1145.69 | 0 | **3190** | HNRNPK | 15 | 104.13 | 0 |
| **3959** | LGALS3BP | 11 | 306.13 | 0 | **10383** | TUBB4B | 26 | 130.06 | 0 | **3315** | HSPB1 | 21 | 349.49 | 0 | **5296** | PIK3R2 | 15 | 89.43 | 0 |
| **7077** | TIMP2 | 11 | 286.15 | 0 | **3848** | KRT1 | 25 | 815.62 | 0 | **5111** | PCNA | 21 | 208.91 | 0 | **29979** | UBQLN1 | 14 | 794.5 | 0 |
| **3688** | ITGB1 | 11 | 167.97 | 0 | **25913** | POT1 | 25 | 261.54 | 0 | **29979** | UBQLN1 | 20 | 1183.42 | 0 | **81** | ACTN4 | 14 | 617.63 | 0 |
| **7083** | TK1 | 11 | 109.11 | 0 | **672** | BRCA1 | 25 | 228.07 | 0 | **87** | ACTN1 | 20 | 338.23 | 0 | **7077** | TIMP2 | 14 | 582.7 | 0 |
| **4088** | SMAD3 | 11 | 108.64 | 0 | **5687** | PSMA6 | 25 | 219.48 | 0 | **3981** | LIG4 | 20 | 335.59 | 0 | **1399** | CRKL | 14 | 418.46 | 0 |
| **1605** | DAG1 | 11 | 91.46 | 0 | **4478** | MSN | 24 | 565.36 | 0 | **5687** | PSMA6 | 20 | 252.76 | 0 | **4830** | NME1 | 14 | 322.56 | 0 |
| **2934** | GSN | 11 | 76.64 | 0 | **6711** | SPTBN1 | 24 | 542.95 | 0 | **3476** | IGBP1 | 20 | 201.62 | 0 | **6130** | RPL7A | 14 | 266.87 | 0 |
| **7086** | TKT | 11 | 24.6 | 0 | **871** | SERPINH1 | 24 | 492.93 | 0 | **7791** | ZYX | 19 | 496.28 | 0 | **8880** | FUBP1 | 14 | 264.7 | 0 |
| **3146** | HMGB1 | 10 | 377.61 | 0 | **6136** | RPL12 | 24 | 461.07 | 0 | **3688** | ITGB1 | 19 | 430.19 | 0 | **3848** | KRT1 | 14 | 179.87 | 0 |
| **7171** | TPM4 | 10 | 191.03 | 0 | **8826** | IQGAP1 | 24 | 325.41 | 0 | **5688** | PSMA7 | 19 | 297.02 | 0 | **8570** | KHSRP | 14 | 171.21 | 0 |
| **3339** | HSPG2 | 10 | 170.19 | 0 | **7001** | PRDX2 | 24 | 265.53 | 0 | **328** | APEX1 | 19 | 243.57 | 0 | **4653** | MYOC | 14 | 88.83 | 0 |
| **6732** | SRPK1 | 10 | 144.47 | 0 | **3315** | HSPB1 | 24 | 257.12 | 0 | **5296** | PIK3R2 | 19 | 184.85 | 0 | **6204** | RPS10 | 14 | 58.65 | 0 |
| **26270** | FBXO6 | 10 | 116.93 | 0 | **3858** | KRT10 | 24 | 233.63 | 0 | **11034** | DSTN | 18 | 986.29 | 0 | **3417** | IDH1 | 13 | 707.35 | 0 |
| **3725** | JUN | 10 | 112.34 | 0 | **5478** | PPIA | 24 | 198.08 | 0 | **3848** | KRT1 | 18 | 702.69 | 0 | **7879** | RAB7A | 13 | 555.67 | 0 |
| **4811** | NID1 | 10 | 109 | 0 | **6927** | HNF1A | 23 | 607.82 | 0 | **10949** | HNRNPA0 | 18 | 506.06 | 0 | **5216** | PFN1 | 13 | 505.6 | 0 |
| **1634** | DCN | 10 | 98.02 | 0 | **7791** | ZYX | 23 | 517.67 | 0 | **5887** | RAD23B | 18 | 380.04 | 0 | **7332** | UBE2L3 | 13 | 498.21 | 0 |
| **9588** | PRDX6 | 10 | 82.36 | 0 | **10146** | G3BP1 | 23 | 495.61 | 0 | **5682** | PSMA1 | 18 | 376.67 | 0 | **4478** | MSN | 13 | 360.97 | 0 |
| **1080** | CFTR | 10 | 81.27 | 0 | **8724** | SNX3 | 23 | 350.5 | 0 | **81** | ACTN4 | 18 | 332.08 | 0 | **7171** | TPM4 | 13 | 251.99 | 0 |
| **25** | ABL1 | 10 | 59.34 | 0 | **1080** | CFTR | 23 | 304.07 | 0 | **7001** | PRDX2 | 18 | 286.22 | 0 | **5479** | PPIB | 13 | 172.15 | 0 |
| **5339** | PLEC | 10 | 53.56 | 0 | **26270** | FBXO6 | 23 | 292.55 | 0 | **8570** | KHSRP | 18 | 279.54 | 0 | **5094** | PCBP2 | 13 | 103.47 | 0 |
| **672** | BRCA1 | 10 | 47.62 | 0 | **3159** | HMGA1 | 23 | 279.45 | 0 | **3858** | KRT10 | 18 | 164.97 | 0 | **2539** | G6PD | 13 | 91.13 | 0 |
| **5591** | PRKDC | 10 | 47 | 0 | **5682** | PSMA1 | 23 | 247.41 | 0 | **25** | ABL1 | 18 | 137.71 | 0 | **1153** | CIRBP | 13 | 88.08 | 0 |
| **79026** | AHNAK | 10 | 19.47 | 0 | **4000** | LMNA | 23 | 139.54 | 0 | **4000** | LMNA | 18 | 110.75 | 0 | **3688** | ITGB1 | 13 | 72.19 | 0 |
| **5309** | PITX3 | 9 | 215.4 | 0 | **10980** | COPS6 | 23 | 96.12 | 0 | **7057** | THBS1 | 17 | 1425.76 | 0 | **7086** | TKT | 13 | 64.45 | 0 |
| **3915** | LAMC1 | 9 | 199.63 | 0 | **1191** | CLU | 22 | 701.63 | 0 | **25824** | PRDX5 | 17 | 507.79 | 0 | **9588** | PRDX6 | 13 | 59.6 | 0 |
| **1398** | CRK | 9 | 112.44 | 0 | **7533** | YWHAH | 22 | 382.71 | 0 | **6927** | HNF1A | 17 | 412.99 | 0 | **4000** | LMNA | 13 | 54.79 | 0 |
| **7052** | TGM2 | 9 | 91.25 | 0 | **4790** | NFKB1 | 22 | 295.38 | 0 | **4478** | MSN | 17 | 366.04 | 0 | **1277** | COL1A1 | 12 | 604.13 | 0 |
| **3068** | HDGF | 9 | 76.45 | 0 | **3981** | LIG4 | 22 | 295.3 | 0 | **1080** | CFTR | 17 | 150.62 | 0 | **7345** | UCHL1 | 12 | 437.2 | 0 |
| **2316** | FLNA | 9 | 34.7 | 0 | **6204** | RPS10 | 22 | 191.88 | 0 | **6204** | RPS10 | 17 | 133.73 | 0 | **396** | ARHGDIA | 12 | 337.83 | 0 |
| **7345** | UCHL1 | 9 | 34.07 | 0 | **7170** | TPM3 | 22 | 185.73 | 0 | **5094** | PCBP2 | 17 | 107.05 | 0 | **10130** | PDIA6 | 12 | 306.19 | 0 |
| **6624** | FSCN1 | 8 | 236.87 | 0 | **6227** | RPS21 | 22 | 118.18 | 0 | **84790** | TUBA1C | 17 | 64.9 | 0 | **7335** | UBE2V1 | 12 | 272.82 | 0 |
| **3488** | IGFBP5 | 8 | 184.9 | 0 | **7057** | THBS1 | 21 | 2772.86 | 0 | **220988** | HNRNPA3 | 17 | 31.72 | 0 | **1958** | EGR1 | 12 | 104.36 | 0 |
| **4312** | MMP1 | 8 | 180.72 | 0 | **9368** | SLC9A3R1 | 21 | 1594.5 | 0 | **1277** | COL1A1 | 16 | 584.87 | 0 | **25** | ABL1 | 12 | 99.1 | 0 |
| **1284** | COL4A2 | 8 | 149.07 | 0 | **4313** | MMP2 | 21 | 1301.47 | 0 | **2192** | FBLN1 | 16 | 569.36 | 0 | **4190** | MDH1 | 12 | 88.31 | 0 |
| **1291** | COL6A1 | 8 | 143.32 | 0 | **5887** | RAD23B | 21 | 534.48 | 0 | **2317** | FLNB | 16 | 449.48 | 0 | **3336** | HSPE1 | 12 | 43.13 | 0 |
| **7040** | TGFB1 | 8 | 141.69 | 0 | **5216** | PFN1 | 21 | 414.37 | 0 | **4830** | NME1 | 16 | 387.41 | 0 | **301** | ANXA1 | 12 | 40.89 | 0 |
| **841** | CASP8 | 8 | 102.17 | 0 | **6206** | RPS12 | 21 | 320.6 | 0 | **5479** | PPIB | 16 | 335.19 | 0 | **6234** | RPS28 | 12 | 40.16 | 0 |
| **1278** | COL1A2 | 8 | 101.66 | 0 | **7171** | TPM4 | 21 | 270.85 | 0 | **396** | ARHGDIA | 16 | 320.66 | 0 | **5230** | PGK 1,00 | 12 | 17.69 | 0 |
| **716** | C1S | 8 | 93.82 | 0 | **301** | ANXA1 | 21 | 249.81 | 0 | **8880** | FUBP1 | 16 | 302.07 | 0 | **2017** | CTTN | 11 | 563.6 | 0 |
| **7273** | TTN | 8 | 93.39 | 0 | **1398** | CRK | 21 | 213.12 | 0 | **4924** | NUCB1 | 16 | 261.97 | 0 | **2271** | FH | 11 | 354.85 | 0 |
| **5296** | PIK3R2 | 8 | 66.86 | 0 | **1915** | EEF1A1 | 21 | 105.14 | 0 | **8724** | SNX3 | 16 | 185.18 | 0 | **5339** | PLEC | 11 | 246.6 | 0 |
| **7276** | TTR | 8 | 58.68 | 0 | **10963** | STIP1 | 21 | 103.54 | 0 | **5216** | PFN1 | 16 | 180.74 | 0 | **6310** | ATXN1 | 11 | 121.6 | 0 |
| **1822** | ATN1 | 8 | 47.12 | 0 | **2896** | GRN | 20 | 1308.52 | 0 | **26270** | FBXO6 | 16 | 108.46 | 0 | **10413** | YAP1 | 11 | 104.07 | 0 |
| **7001** | PRDX2 | 8 | 38.72 | 0 | **1277** | COL1A1 | 20 | 928.5 | 0 | **3336** | HSPE1 | 16 | 105.57 | 0 | **4191** | MDH2 | 11 | 73.56 | 0 |
| **22976** | PAXIP1 | 8 | 31.76 | 0 | **81** | ACTN4 | 20 | 443.85 | 0 | **5230** | PGK 1,00 | 16 | 67.51 | 0 | **7052** | TGM2 | 11 | 67.12 | 0 |
| **5536** | PPP5C | 8 | 29.99 | 0 | **8407** | TAGLN2 | 20 | 319.39 | 0 | **8407** | TAGLN2 | 15 | 404.23 | 0 | **1207** | CLNS1A | 11 | 56.97 | 0 |
| **7514** | XPO1 | 8 | 29.69 | 0 | **328** | APEX1 | 20 | 234.88 | 0 | **7171** | TPM4 | 15 | 285.11 | 0 | **54386** | TERF2IP | 11 | 53.47 | 0 |
| **9759** | HDAC4 | 7 | 128.23 | 0 | **3476** | IGBP1 | 20 | 232.93 | 0 | **7052** | TGM2 | 15 | 213.03 | 0 | **7014** | TERF2 | 11 | 49.64 | 0 |
| **6314** | ATXN7 | 7 | 78.54 | 0 | **5111** | PCNA | 20 | 154.85 | 0 | **4790** | NFKB1 | 15 | 182.5 | 0 | **10189** | ALYREF | 10 | 403.29 | 0 |

| **Names** | **total** | **elements** |
| --- | --- | --- |
| **CT 10D 30D 6D0** | 25 | ACTB |
|  |  | ENO1 |
|  |  | HSPA5 |
|  |  | VCL |
|  |  | MYH9 |
|  |  | ATF2 |
|  |  | GAPDH |
|  |  | PXN |
|  |  | KIAA0101 |
|  |  | LDHB |
|  |  | VIM |
|  |  | GJA1 |
|  |  | ELAVL1 |
|  |  | LDHA |
|  |  | APP |
|  |  | VHL |
|  |  | SP1 |
|  |  | CDK2 |
|  |  | FN1 |
|  |  | UBC |
|  |  | HSP90AA1 |
|  |  | YWHAZ |
|  |  | ESR1 |
|  |  | HSPA1A |
|  |  | GRB2 |
| **C10 IR10 IR30** | 4 | CFL1 |
|  |  | YWHAG |
|  |  | PKM |
|  |  | PDIA3 |
| **C10 IR30 IR60** | 1 | EEF1A1 |
| **IR10 IR30 IR60** | 19 | ANXA2 |
|  |  | LGALS3 |
|  |  | TP53 |
|  |  | NPM1 |
|  |  | HMGA1 |
|  |  | MYC |
|  |  | CRK |
|  |  | PRDX1 |
|  |  | ALDOA |
|  |  | PSMA3 |
|  |  | FLNA |
|  |  | CAND1 |
|  |  | HNRNPA1 |
|  |  | HNF4A |
|  |  | HSPA8 |
|  |  | CTNNB1 |
|  |  | EGFR |
|  |  | LGALS1 |
|  |  | POT1 |
| **C10 IR10** | 2 | ALB |
|  |  | HSP90AB1 |
| **IR10 IR30** | 24 | LIG4 |
|  |  | MMP2 |
|  |  | YBX1 |
|  |  | PSMA6 |
|  |  | HSPB1 |
|  |  | YWHAQ |
|  |  | G3BP1 |
|  |  | CUL4B |
|  |  | TLN1 |
|  |  | ATP5B |
|  |  | HNRNPD |
|  |  | ACTG1 |
|  |  | NONO |
|  |  | EZR |
|  |  | VCP |
|  |  | BRCA1 |
|  |  | SPTAN1 |
|  |  | IGBP1 |
|  |  | AKT1 |
|  |  | IQCB1 |
|  |  | HNRNPA2B1 |
|  |  | GRN |
|  |  | HNRNPK |
|  |  | P4HB |
| **IR10 IR60** | 1 | HSPD1 |
| **IR30 IR60** | 3 | EWSR1 |
|  |  | PCBP1 |
|  |  | YWHAB |
| **C10** | 2 | H2AFX |
|  |  | A2M |
| **IR10** | 43 | ACTN4 |
|  |  | IQGAP1 |
|  |  | RPL12 |
|  |  | RPL10A |
|  |  | VCAM1 |
|  |  | RPS12 |
|  |  | YWHAH |
|  |  | CLU |
|  |  | ANXA1 |
|  |  | FBXO6 |
|  |  | EEF2 |
|  |  | HNF1A |
|  |  | HNRNPM |
|  |  | APEX1 |
|  |  | SPTBN1 |
|  |  | ZYX |
|  |  | THBS1 |
|  |  | SERPINH1 |
|  |  | MSN |
|  |  | HLA-B |
|  |  | PSMA7 |
|  |  | EIF1B |
|  |  | TPM4 |
|  |  | HSP90B1 |
|  |  | PSMA1 |
|  |  | KRT1 |
|  |  | HSPA4 |
|  |  | SUMO2 |
|  |  | PAN2 |
|  |  | PSMA5 |
|  |  | COL1A1 |
|  |  | KRT10 |
|  |  | RPSA |
|  |  | SLC9A3R1 |
|  |  | SNX3 |
|  |  | CFTR |
|  |  | TAGLN2 |
|  |  | NFKB1 |
|  |  | PFN1 |
|  |  | RAD23B |
|  |  | MYL12A |
|  |  | PRDX2 |
|  |  | ITGA4 |
| **IR30** | 10 | TUBB |
|  |  | UBQLN1 |
|  |  | PPIA |
|  |  | ACTN1 |
|  |  | EFEMP2 |
|  |  | PCNA |

| **6310** | ATXN1 | 7 | 68.57 | 0 | **27316** | RBMX | 20 | 148.89 | 0 | **27316** | RBMX | 15 | 179.41 | 0 | **5037** | PEBP1 | 10 | 261.91 | 0 |
| --- | --- | --- | --- | --- | --- | --- | --- | --- | --- | --- | --- | --- | --- | --- | --- | --- | --- | --- | --- |
| **5340** | PLG | 7 | 55.88 | 0 | **6234** | RPS28 | 20 | 80.27 | 0 | **2033** | EP300 | 15 | 145.52 | 0 | **30851** | TAX1BP3 | 10 | 173.23 | 0 |
| **11315** | PARK7 | 7 | 55.15 | 0 | **3336** | HSPE1 | 19 | 274.76 | 0 | **4088** | SMAD3 | 15 | 128.41 | 0 | **7295** | TXN | 10 | 126.88 | 0 |
| **1281** | COL3A1 | 7 | 39.69 | 0 | **3068** | HDGF | 19 | 251.05 | 0 | **1153** | CIRBP | 15 | 113.94 | 0 | **5935** | RBM3 | 10 | 123.71 | 0 |
| **6418** | SET | 7 | 37.28 | 0 | **8570** | KHSRP | 19 | 240.67 | 0 | **10963** | STIP1 | 15 | 99.72 | 0 | **308** | ANXA5 | 10 | 98.07 | 0 |
| **3065** | HDAC1 | 7 | 35.61 | 0 | **2317** | FLNB | 19 | 191.68 | 0 | **1207** | CLNS1A | 15 | 87.71 | 0 | **3849** | KRT2 | 10 | 89.87 | 0 |
| **773** | CACNA1A | 7 | 26.51 | 0 | **7168** | TPM1 | 19 | 161.9 | 0 | **22976** | PAXIP1 | 15 | 86.59 | 0 | **3857** | KRT9 | 10 | 83.52 | 0 |
| **5037** | PEBP1 | 7 | 23.07 | 0 | **11140** | CDC37 | 18 | 253.04 | 0 | **6234** | RPS28 | 15 | 66.16 | 0 | **26270** | FBXO6 | 10 | 83.04 | 0 |
| **4350** | MPG | 7 | 21.28 | 0 | **10097** | ACTR2 | 18 | 222.36 | 0 | **6227** | RPS21 | 15 | 64.12 | 0 | **580** | BARD1 | 10 | 72.8 | 0 |
| **6278** | S100A7 | 7 | 19.53 | 0 | **4088** | SMAD3 | 18 | 217.63 | 0 | **3182** | HNRNPAB | 15 | 33.27 | 0 | **4282** | MIF | 10 | 66.19 | 0 |
| **5501** | PPP1CC | 6 | 143.04 | 0 | **25** | ABL1 | 18 | 191.66 | 0 | **5054** | SERPINE1 | 14 | 953.87 | 0 | **2934** | GSN | 10 | 50.93 | 0 |
| **2006** | ELN | 6 | 100.98 | 0 | **10487** | CAP1 | 18 | 185.73 | 0 | **3417** | IDH1 | 14 | 610.31 | 0 | **71** | ACTG1 | 10 | 42.67 | 0 |
| **4082** | MARCKS | 6 | 63.49 | 0 | **5339** | PLEC | 18 | 185.47 | 0 | **5339** | PLEC | 14 | 312.26 | 0 | **7170** | TPM3 | 10 | 39.23 | 0 |
| **55729** | ATF7IP | 6 | 58.02 | 0 | **4653** | MYOC | 18 | 172.78 | 0 | **197** | AHSG | 14 | 235.86 | 0 | **2318** | FLNC | 10 | 38.17 | 0 |
| **8425** | LTBP4 | 6 | 52.66 | 0 | **5296** | PIK3R2 | 18 | 166.65 | 0 | **6206** | RPS12 | 14 | 217.94 | 0 | **5054** | SERPINE1 | 9 | 856.37 | 0 |
| **4E+05** | AGRN | 6 | 48.38 | 0 | **22976** | PAXIP1 | 18 | 116.44 | 0 | **3569** | IL6 | 14 | 170.12 | 0 | **1476** | CSTB | 9 | 411.22 | 0 |
| **2547** | XRCC6 | 6 | 37.84 | 0 | **3857** | KRT9 | 18 | 115.34 | 0 | **301** | ANXA1 | 14 | 110.83 | 0 | **5309** | PITX3 | 9 | 400.56 | 0 |
| **4035** | LRP1 | 6 | 36.5 | 0 | **25824** | PRDX5 | 17 | 496.94 | 0 | **7514** | XPO1 | 14 | 109.6 | 0 | **1278** | COL1A2 | 9 | 284.44 | 0 |
| **22795** | NID2 | 6 | 33.03 | 0 | **2017** | CTTN | 17 | 479.19 | 0 | **9588** | PRDX6 | 14 | 59.88 | 0 | **2950** | GSTP1 | 9 | 209.36 | 0 |
| **11167** | FSTL1 | 6 | 24.68 | 0 | **3039** | HBA1 | 17 | 379.16 | 0 | **4653** | MYOC | 14 | 49.79 | 0 | **84305** | WIBG | 9 | 124.8 | 0 |
| **5887** | RAD23B | 6 | 22.54 | 0 | **8880** | FUBP1 | 17 | 304.5 | 0 | **3486** | IGFBP3 | 13 | 846.77 | 0 | **54205** | CYCS | 9 | 109.39 | 0 |
| **2773** | GNAI3 | 6 | 20.78 | 0 | **567** | B2M | 17 | 227.97 | 0 | **1958** | EGR1 | 13 | 591.63 | 0 | **4134** | MAP4 | 9 | 85.79 | 0 |
| **10409** | BASP1 | 6 | 18.05 | 0 | **1025** | CDK9 | 17 | 94.08 | 0 | **7345** | UCHL1 | 13 | 528.78 | 0 | **3725** | JUN | 9 | 68.34 | 0 |
| **558** | AXL | 6 | 15.85 | 0 | **3849** | KRT2 | 17 | 92.25 | 0 | **5110** | PCMT1 | 13 | 425.21 | 0 | **5594** | MAPK1 | 9 | 61.3 | 0 |
| **1289** | COL5A1 | 5 | 292.81 | 0 | **5230** | PGK 1,00 | 17 | 55.73 | 0 | **6624** | FSCN1 | 13 | 387.32 | 0 | **5536** | PPP5C | 9 | 35.14 | 0 |
| **3927** | LASP1 | 5 | 203.74 | 0 | **84790** | TUBA1C | 17 | 44.54 | 0 | **5501** | PPP1CC | 13 | 339.33 | 0 | **79026** | AHNAK | 9 | 27.46 | 0 |
| **3490** | IGFBP7 | 5 | 197.25 | 0 | **3182** | HNRNPAB | 17 | 36.99 | 0 | **6310** | ATXN1 | 13 | 254.82 | 0 | **6275** | S100A4 | 8 | 551.74 | 0 |
| **5499** | PPP1CA | 5 | 146.62 | 0 | **5309** | PITX3 | 16 | 553.63 | 0 | **6732** | SRPK1 | 13 | 223.87 | 0 | **6281** | S100A10 | 8 | 477.27 | 0 |
| **5270** | SERPINE2 | 5 | 68.19 | 0 | **2318** | FLNC | 16 | 476.18 | 0 | **2318** | FLNC | 13 | 205.57 | 0 | **8148** | TAF15 | 8 | 230.29 | 0 |
| **633** | BGN | 5 | 65.69 | 0 | **3569** | IL6 | 16 | 435.61 | 0 | **3725** | JUN | 13 | 162.47 | 0 | **5270** | SERPINE2 | 8 | 214.74 | 0 |
| **9869** | SETDB1 | 5 | 62.99 | 0 | **6624** | FSCN1 | 16 | 426.12 | 0 | **2934** | GSN | 13 | 144.54 | 0 | **2280** | FKBP1A | 8 | 214.1 | 0 |
| **4099** | MAG | 5 | 52.85 | 0 | **5479** | PPIB | 16 | 363.01 | 0 | **10487** | CAP1 | 13 | 130.66 | 0 | **3488** | IGFBP5 | 8 | 141.45 | 0 |
| **6498** | SKIL | 5 | 47.56 | 0 | **23193** | GANAB | 16 | 230.15 | 0 | **26135** | SERBP1 | 13 | 124.28 | 0 | **3006** | HIST1H1C | 8 | 127.46 | 0 |
| **1471** | CST3 | 5 | 45.62 | 0 | **2271** | FH | 16 | 168.52 | 0 | **2026** | ENO2 | 13 | 116.83 | 0 | **7360** | UGP2 | 8 | 125.13 | 0 |
| **5687** | PSMA6 | 5 | 41.7 | 0 | **3925** | STMN1 | 16 | 164.06 | 0 | **3849** | KRT2 | 13 | 78.4 | 0 | **9255** | AIMP1 | 8 | 114.69 | 0 |
| **1894** | ECT2 | 5 | 41.12 | 0 | **3688** | ITGB1 | 16 | 148.18 | 0 | **4191** | MDH2 | 13 | 69.48 | 0 | **5954** | RCN1 | 8 | 57.97 | 0 |
| **7422** | VEGFA | 5 | 40 | 0 | **829** | CAPZA1 | 16 | 126.05 | 0 | **580** | BARD1 | 13 | 66 | 0 | **2353** | FOS | 8 | 47.25 | 0 |
| **51150** | SDF4 | 5 | 33.11 | 0 | **4282** | MIF | 16 | 108.92 | 0 | **4350** | MPG | 13 | 61.65 | 0 | **1211** | CLTA | 8 | 40.48 | 0 |
| **2353** | FOS | 5 | 20.92 | 0 | **4191** | MDH2 | 16 | 88.71 | 0 | **7086** | TKT | 13 | 54.49 | 0 | **7528** | YY1 | 8 | 35.13 | 0 |
| **8626** | TP63 | 5 | 16.64 | 0 | **9588** | PRDX6 | 16 | 56.12 | 0 | **5309** | PITX3 | 12 | 324.95 | 0 | **5049** | PAFAH1B2 | 8 | 27.36 | 0 |
| **6462** | SHBG | 5 | 16.5 | 0 | **5054** | SERPINE1 | 15 | 989.62 | 0 | **7077** | TIMP2 | 12 | 274.69 | 0 | **9531** | BAG3 | 7 | 413.11 | 0 |
| **4846** | NOS3 | 5 | 11.24 | 0 | **2192** | FBLN1 | 15 | 914.4 | 0 | **1399** | CRKL | 12 | 259.1 | 0 | **203** | AK1 | 7 | 404.63 | 0 |
| **6282** | S100A11 | 5 | 5.64 | 0 | **1958** | EGR1 | 15 | 622.8 | 0 | **3006** | HIST1H1C | 12 | 248.5 | 0 | **2078** | ERG | 7 | 248.7 | 0 |
| **712** | C1QA | 4 | 213.43 | 0 | **6678** | SPARC | 15 | 348.77 | 0 | **1634** | DCN | 12 | 176.6 | 0 | **3927** | LASP1 | 7 | 221.25 | 0 |
| **3481** | IGF2 | 4 | 205.18 | 0 | **9601** | PDIA4 | 15 | 285.47 | 0 | **56893** | UBQLN4 | 12 | 146.49 | 0 | **2287** | FKBP3 | 7 | 128.99 | 0 |
| **5589** | PRKCSH | 4 | 194.57 | 0 | **7052** | TGM2 | 15 | 230.6 | 0 | **829** | CAPZA1 | 12 | 143.84 | 0 | **7458** | EIF4H | 7 | 93.03 | 0 |
| **2817** | GPC1 | 4 | 166.46 | 0 | **23603** | CORO1C | 15 | 221.99 | 0 | **367** | AR | 12 | 97.51 | 0 | **11315** | PARK7 | 7 | 86.14 | 0 |
| **7076** | TIMP1 | 4 | 166 | 0 | **4924** | NUCB1 | 15 | 220.71 | 0 | **7083** | TK1 | 12 | 95.02 | 0 | **558** | AXL | 7 | 76.71 | 0 |
| **6442** | SGCA | 4 | 54.85 | 0 | **7514** | XPO1 | 15 | 100.4 | 0 | **310** | ANXA7 | 12 | 87.79 | 0 | **2869** | GRK5 | 7 | 64.21 | 0 |
| **7048** | TGFBR2 | 4 | 54.46 | 0 | **2869** | GRK5 | 15 | 97.23 | 0 | **9601** | PDIA4 | 12 | 79.61 | 0 | **11100** | HNRNPUL | 7 | 50.59 | 0 |
| **5864** | RAB3A | 4 | 54.16 | 0 | **1207** | CLNS1A | 15 | 83.95 | 0 | **5591** | PRKDC | 12 | 75.42 | 0 | **10521** | DDX17 | 7 | 48.36 | 0 |
| **4926** | NUMA1 | 4 | 44.13 | 0 | **5536** | PPP5C | 15 | 77.78 | 0 | **5049** | PAFAH1B2 | 12 | 74.21 | 0 | **10935** | PRDX3 | 7 | 36.32 | 0 |
| **2199** | FBLN2 | 4 | 43.61 | 0 | **10130** | PDIA6 | 14 | 653.19 | 0 | **5720** | PSME1 | 12 | 53.63 | 0 | **10226** | PLIN3 | 7 | 33.15 | 0 |
| **57761** | TRIB3 | 4 | 33.93 | 0 | **197** | AHSG | 14 | 399.33 | 0 | **5935** | RBM3 | 12 | 19.89 | 0 | **81567** | TXNDC5 | 7 | 27.49 | 0 |
| **2243** | FGA | 4 | 26.8 | 0 | **7345** | UCHL1 | 14 | 287.5 | 0 | **4312** | MMP1 | 11 | 595.57 | 0 | **6624** | FSCN1 | 7 | 11.52 | 0 |
| **5155** | PDGFB | 4 | 26.43 | 0 | **7077** | TIMP2 | 14 | 270.93 | 0 | **1476** | CSTB | 11 | 467.18 | 0 | **38** | ACAT1 | 6 | 681.53 | 0 |
| **3337** | DNAJB1 | 4 | 25.26 | 0 | **3910** | LAMA4 | 14 | 213.05 | 0 | **7453** | WARS | 11 | 244.62 | 0 | **4312** | MMP1 | 6 | 653.13 | 0 |
| **8291** | DYSF | 4 | 24.41 | 0 | **3725** | JUN | 14 | 153.68 | 0 | **47** | ACLY | 11 | 232.66 | 0 | **6434** | TRA2B | 6 | 308.16 | 0 |
| **1051** | CEBPB | 4 | 23.32 | 0 | **396** | ARHGDIA | 14 | 146.43 | 0 | **6347** | CCL2 | 11 | 184.47 | 0 | **9260** | PDLIM7 | 6 | 297.34 | 0 |
| **10540** | DCTN2 | 4 | 21.03 | 0 | **2547** | XRCC6 | 14 | 110.49 | 0 | **1284** | COL4A2 | 11 | 176.71 | 0 | **25890** | ABI3BP | 6 | 112.38 | 0 |
| **1786** | DNMT1 | 4 | 20.79 | 0 | **4350** | MPG | 14 | 72.21 | 0 | **7295** | TXN | 11 | 130.35 | 0 | **2113** | ETS1 | 6 | 107.83 | 0 |
| **10726** | NUDC | 4 | 18.79 | 0 | **3486** | IGFBP3 | 13 | 858.1 | 0 | **9948** | WDR1 | 11 | 87.89 | 0 | **4082** | MARCKS | 6 | 100.26 | 0 |
| **5806** | PTX3 | 4 | 17.43 | 0 | **2950** | GSTP1 | 13 | 431.97 | 0 | **2869** | GRK5 | 11 | 75.31 | 0 | **8721** | EDF1 | 6 | 97.21 | 0 |
| **89953** | KLC4 | 4 | 16.88 | 0 | **6347** | CCL2 | 13 | 250.46 | 0 | **4190** | MDH1 | 11 | 75.29 | 0 | **79180** | EFHD2 | 6 | 75.5 | 0 |
| **1009** | CDH11 | 4 | 16.49 | 0 | **9961** | MVP | 13 | 247.3 | 0 | **10189** | ALYREF | 11 | 70.74 | 0 | **6648** | SOD2 | 6 | 64.98 | 0 |
| **1958** | EGR1 | 4 | 15.23 | 0 | **4735** | SEPT2 | 13 | 218.53 | 0 | **4282** | MIF | 11 | 69.28 | 0 | **6902** | TBCA | 6 | 64.23 | 0 |
| **1808** | DPYSL2 | 4 | 14.78 | 0 | **1278** | COL1A2 | 13 | 201.41 | 0 | **81567** | TXNDC5 | 11 | 63.36 | 0 | **2810** | SFN | 6 | 59.73 | 0 |
| **338** | APOB | 4 | 14.09 | 0 | **10801** | SEPT9 | 13 | 135 | 0 | **58** | ACTA1 | 11 | 57.18 | 0 | **4314** | MMP3 | 6 | 57.61 | 0 |
| **2200** | FBN1 | 4 | 11.58 | 0 | **5902** | RANBP1 | 13 | 134.09 | 0 | **6606** | SMN1 | 11 | 56.6 | 0 | **1845** | DUSP3 | 6 | 51.24 | 0 |
| **2771** | GNAI2 | 4 | 11.21 | 0 | **55748** | CNDP2 | 13 | 131.83 | 0 | **79026** | AHNAK | 11 | 26.07 | 0 | **847** | CAT | 6 | 49.23 | 0 |
| **1435** | CSF1 | 4 | 10.74 | 0 | **4190** | MDH1 | 13 | 117.95 | 0 | **2950** | GSTP1 | 10 | 320.96 | 0 | **10043** | TOM1 | 6 | 48.8 | 0 |
| **3371** | TNC | 4 | 9.59 | 0 | **7408** | VASP | 13 | 101.04 | 0 | **5037** | PEBP1 | 10 | 240.1 | 0 | **1509** | CTSD | 6 | 46.85 | 0 |
| **8764** | TNFRSF14 | 4 | 8.67 | 0 | **3065** | HDAC1 | 13 | 97.8 | 0 | **7311** | UBA52 | 10 | 214.67 | 0 | **4093** | SMAD9 | 6 | 38.7 | 0 |
| **11117** | EMILIN1 | 4 | 7.28 | 0 | **2033** | EP300 | 13 | 74.04 | 0 | **6678** | SPARC | 10 | 196.71 | 0 | **800** | CALD1 | 6 | 35.66 | 0 |
| **6434** | TRA2B | 3 | 186.56 | 0 | **87** | ACTN1 | 13 | 71.41 | 0 | **2287** | FKBP3 | 10 | 156.21 | 0 | **204** | AK2 | 6 | 35.42 | 0 |
| **509** | ATP5C1 | 3 | 128.48 | 0 | **5591** | PRKDC | 13 | 65.39 | 0 | **4134** | MAP4 | 10 | 154.79 | 0 | **9124** | PDLIM1 | 6 | 34.83 | 0 |
| **4323** | MMP14 | 3 | 99.66 | 0 | **6195** | RPS6KA1 | 13 | 65.21 | 0 | **841** | CASP8 | 10 | 134.34 | 0 | **10409** | BASP1 | 6 | 30.39 | 0 |
| **2247** | FGF2 | 3 | 71.54 | 0 | **5597** | MAPK6 | 13 | 57.73 | 0 | **4089** | SMAD4 | 10 | 121.45 | 0 | **355** | FAS | 6 | 29.23 | 0 |
| **7917** | BAG6 | 3 | 41.83 | 0 | **7086** | TKT | 13 | 54.18 | 0 | **84305** | WIBG | 10 | 121.12 | 0 | **5879** | RAC1 | 6 | 27.24 | 0 |
| **5688** | PSMA7 | 3 | 38.68 | 0 | **3959** | LGALS3B | 12 | 583.73 | 0 | **6888** | TALDO1 | 10 | 114.68 | 0 | **5223** | PGAM1 | 6 | 27.22 | 0 |
| **94121** | SYTL4 | 3 | 33.26 | 0 | **1605** | DAG1 | 12 | 557.81 | 0 | **1894** | ECT2 | 10 | 102.73 | 0 | **95** | ACY1 | 6 | 26.01 | 0 |
| **1508** | CTSB | 3 | 30.72 | 0 | **4312** | MMP1 | 12 | 483.66 | 0 | **11315** | PARK7 | 10 | 97.61 | 0 | **6282** | S100A11 | 6 | 21.65 | 0 |
| **1303** | COL12A1 | 3 | 26.84 | 0 | **5501** | PPP1CC | 12 | 430.2 | 0 | **55748** | CNDP2 | 10 | 95.72 | 0 | **6195** | RPS6KA1 | 6 | 18.81 | 0 |
| **6696** | SPP1 | 3 | 24.5 | 0 | **1476** | CSTB | 12 | 359.72 | 0 | **7528** | YY1 | 10 | 91.82 | 0 | **1738** | DLD | 6 | 10.64 | 0 |
| **30846** | EHD2 | 3 | 24.11 | 0 | **7169** | TPM2 | 12 | 296.18 | 0 | **6418** | SET | 10 | 70.51 | 0 | **284119** | PTRF | 5 | 392.75 | 0 |
| **649** | BMP1 | 3 | 22.98 | 0 | **4830** | NME1 | 12 | 171.2 | 0 | **3959** | LGALS3B | 10 | 54.28 | 0 | **3659** | IRF1 | 5 | 391.73 | 0 |
| **93** | ACVR2B | 3 | 21.87 | 0 | **5962** | RDX | 12 | 144.41 | 0 | **5536** | PPP5C | 10 | 53.11 | 0 | **8655** | DYNLL1 | 5 | 93.13 | 0 |
| **2118** | ETV4 | 3 | 21.18 | 0 | **2934** | GSN | 12 | 92.71 | 0 | **6195** | RPS6KA1 | 10 | 47.9 | 0 | **5701** | PSMC2 | 5 | 85.43 | 0 |
| **22883** | CLSTN1 | 3 | 21.1 | 0 | **54205** | CYCS | 12 | 90.5 | 0 | **1984** | EIF5A | 10 | 41.08 | 0 | **9446** | GSTO1 | 5 | 71.83 | 0 |
| **7453** | WARS | 3 | 20.27 | 0 | **1211** | CLTA | 12 | 40.19 | 0 | **7296** | TXNRD1 | 10 | 32.53 | 0 | **10493** | VAT1 | 5 | 69.23 | 0 |
| **11100** | HNRNPUL1 | 3 | 17.48 | 0 | **79026** | AHNAK | 12 | 27.05 | 0 | **1508** | CTSB | 9 | 503.94 | 0 | **8417** | STX7 | 5 | 52.02 | 0 |
| **1893** | ECM1 | 3 | 17 | 0 | **56893** | UBQLN4 | 11 | 554.12 | 0 | **3915** | LAMC1 | 9 | 296.56 | 0 | **7322** | UBE2D2 | 5 | 44.53 | 0 |
| **29979** | UBQLN1 | 3 | 16.86 | 0 | **1634** | DCN | 11 | 541.51 | 0 | **2078** | ERG | 9 | 273.57 | 0 | **2936** | GSR | 5 | 44.44 | 0 |
| **1991** | ELANE | 3 | 15.77 | 0 | **2273** | FHL1 | 11 | 361.2 | 0 | **3488** | IGFBP5 | 9 | 251.62 | 0 | **6238** | RRBP1 | 5 | 44.34 | 0 |
| **10053** | AP1M2 | 3 | 15.05 | 0 | **9124** | PDLIM1 | 11 | 176.69 | 0 | **9531** | BAG3 | 9 | 225.43 | 0 | **1471** | CST3 | 5 | 39.57 | 0 |
| **710** | SERPING1 | 3 | 14.84 | 0 | **841** | CASP8 | 11 | 162.55 | 0 | **8148** | TAF15 | 9 | 144.52 | 0 | **136319** | MTPN | 5 | 38.86 | 0 |
| **3175** | ONECUT1 | 3 | 14.79 | 0 | **2908** | NR3C1 | 11 | 160.82 | 0 | **5962** | RDX | 9 | 132.86 | 0 | **25796** | PGLS | 5 | 33.39 | 0 |
| **7373** | COL14A1 | 3 | 13.83 | 0 | **10549** | PRDX4 | 11 | 157.86 | 0 | **2** | A2M | 9 | 104.04 | 0 | **3151** | HMGN2 | 5 | 26.53 | 0 |
| **9520** | NPEPPS | 3 | 10.86 | 0 | **5037** | PEBP1 | 11 | 145.96 | 0 | **5970** | RELA | 9 | 94.82 | 0 | **1822** | ATN1 | 5 | 25.7 | 0 |
| **5933** | RBL1 | 3 | 10.51 | 0 | **9531** | BAG3 | 11 | 142.53 | 0 | **5954** | RCN1 | 9 | 90 | 0 | **1508** | CTSB | 5 | 21.4 | 0 |
| **1000** | CDH2 | 3 | 10.35 | 0 | **4134** | MAP4 | 11 | 139.66 | 0 | **54205** | CYCS | 9 | 81.1 | 0 | **81488** | GCOM1 | 5 | 19.96 | 0 |
| **720** | C4A | 3 | 9.33 | 0 | **7528** | YY1 | 11 | 136.71 | 0 | **7020** | TFAP2A | 9 | 72.91 | 0 | **6652** | SORD | 5 | 19.36 | 0 |
| **1293** | COL6A3 | 3 | 8.29 | 0 | **6310** | ATXN1 | 11 | 122.4 | 0 | **23327** | NEDD4L | 9 | 70.55 | 0 | **23410** | SIRT3 | 5 | 18.95 | 0 |
| **79001** | VKORC1 | 3 | 6.68 | 0 | **11315** | PARK7 | 11 | 111.04 | 0 | **30851** | TAX1BP3 | 9 | 69.3 | 0 | **231** | AKR1B1 | 5 | 18.66 | 0 |
| **4192** | MDK | 3 | 6.47 | 0 | **6772** | STAT1 | 11 | 106.24 | 0 | **2547** | XRCC6 | 9 | 60.13 | 0 | **2969** | GTF2I | 5 | 17.48 | 0 |
| **3096** | HIVEP1 | 3 | 5.98 | 0 | **7295** | TXN | 11 | 104.58 | 0 | **5223** | PGAM1 | 9 | 56.12 | 0 | **6670** | SP3 | 5 | 16.43 | 0 |
| **3698** | ITIH2 | 3 | 5.43 | 0 | **2534** | FYN | 11 | 68.24 | 0 | **2773** | GNAI3 | 9 | 55.91 | 0 | **10473** | HMGN4 | 5 | 15.15 | 0 |
| **10944** | C11orf58 | 3 | 4.82 | 0 | **580** | BARD1 | 11 | 64.1 | 0 | **2353** | FOS | 9 | 52.74 | 0 | **7020** | TFAP2A | 5 | 13.6 | 0 |
| **2E+05** | USP12 | 3 | 3.98 | 0 | **81567** | TXNDC5 | 11 | 60.83 | 0 | **10010** | TANK | 9 | 47.06 | 0 | **3643** | INSR | 5 | 12.8 | 0 |
| **1292** | COL6A2 | 3 | 3.55 | 0 | **292** | SLC25A5 | 11 | 53.21 | 0 | **10226** | PLIN3 | 9 | 45.1 | 0 | **2806** | GOT2 | 5 | 11.43 | 0 |
| **8614** | STC2 | 3 | 3.08 | 0 | **1192** | CLIC1 | 11 | 40.66 | 0 | **3925** | STMN1 | 9 | 39.46 | 0 | **3925** | STMN1 | 5 | 5.32 | 0 |
| **6876** | TAGLN | 3 | 2.87 | 0 | **3488** | IGFBP5 | 10 | 353 | 0 | **1605** | DAG1 | 9 | 38.59 | 0 | **522** | ATP5J | 4 | 568.69 | 0 |
| **3101** | HK3 | 2 | 89.67 | 0 | **9759** | HDAC4 | 10 | 315.18 | 0 | **1211** | CLTA | 9 | 34.05 | 0 | **3490** | IGFBP7 | 4 | 563.04 | 0 |
| **2274** | FHL2 | 2 | 45.75 | 0 | **3339** | HSPG2 | 10 | 275.99 | 0 | **718** | C3 | 8 | 584.14 | 0 | **9319** | TRIP13 | 4 | 255.67 | 0 |
| **10653** | SPINT2 | 2 | 35.13 | 0 | **7453** | WARS | 10 | 199.28 | 0 | **1291** | COL6A1 | 8 | 419.41 | 0 | **51631** | LUC7L2 | 4 | 211.18 | 0 |
| **56931** | DUS3L | 2 | 31.94 | 0 | **1284** | COL4A2 | 10 | 181.58 | 0 | **716** | C1S | 8 | 316.77 | 0 | **57562** | KIAA1377 | 4 | 144.65 | 0 |
| **3075** | CFH | 2 | 31.61 | 0 | **2287** | FKBP3 | 10 | 165.78 | 0 | **9759** | HDAC4 | 8 | 241.63 | 0 | **23474** | ETHE1 | 4 | 134.66 | 0 |
| **8600** | TNFSF11 | 2 | 27.4 | 0 | **2078** | ERG | 10 | 148.09 | 0 | **5499** | PPP1CA | 8 | 237.97 | 0 | **84817** | TXNDC17 | 4 | 117.66 | 0 |
| **5768** | QSOX1 | 2 | 26.71 | 0 | **1894** | ECT2 | 10 | 120.33 | 0 | **213** | ALB | 8 | 185.77 | 0 | **2821** | GPI | 4 | 104.33 | 0 |
| **2669** | GEM | 2 | 25.98 | 0 | **55729** | ATF7IP | 10 | 97.26 | 0 | **8985** | PLOD3 | 8 | 162.04 | 0 | **10802** | SEC24A | 4 | 102.72 | 0 |
| **6403** | SELP | 2 | 24.74 | 0 | **10540** | DCTN2 | 10 | 90.2 | 0 | **10611** | PDLIM5 | 8 | 145.33 | 0 | **7917** | BAG6 | 4 | 48.9 | 0 |
| **3211** | HOXB1 | 2 | 18.7 | 0 | **5970** | RELA | 10 | 82.84 | 0 | **6652** | SORD | 8 | 93.73 | 0 | **6696** | SPP1 | 4 | 42.04 | 0 |
| **2273** | FHL1 | 2 | 12.18 | 0 | **310** | ANXA7 | 10 | 77.86 | 0 | **5359** | PLSCR1 | 8 | 83.99 | 0 | **8091** | HMGA2 | 4 | 39.74 | 0 |
| **148** | ADRA1A | 2 | 11.81 | 0 | **6418** | SET | 10 | 72.45 | 0 | **6238** | RRBP1 | 8 | 81.82 | 0 | **2623** | GATA1 | 4 | 36.22 | 0 |
| **57596** | BEGAIN | 2 | 11.79 | 0 | **5358** | PLS3 | 10 | 61.65 | 0 | **2280** | FKBP1A | 8 | 76.28 | 0 | **6844** | VAMP2 | 4 | 33.14 | 0 |
| **1E+05** | VASN | 2 | 11.79 | 0 | **2773** | GNAI3 | 10 | 57.08 | 0 | **1822** | ATN1 | 8 | 68.83 | 0 | **10449** | ACAA2 | 4 | 27.65 | 0 |
| **3487** | IGFBP4 | 2 | 10.24 | 0 | **23410** | SIRT3 | 10 | 56.37 | 0 | **773** | CACNA1A | 8 | 58.35 | 0 | **290** | ANPEP | 4 | 25.77 | 0 |
| **5754** | PTK7 | 2 | 6.96 | 0 | **58** | ACTA1 | 10 | 54.88 | 0 | **4093** | SMAD9 | 8 | 57.85 | 0 | **56902** | PNO1 | 4 | 22.94 | 0 |
| **4982** | TNFRSF11B | 2 | 6.67 | 0 | **9255** | AIMP1 | 10 | 44.24 | 0 | **5879** | RAC1 | 8 | 56.07 | 0 | **327** | APEH | 4 | 19.46 | 0 |
| **1476** | CSTB | 2 | 6.43 | 0 | **5049** | PAFAH1B2 | 10 | 34.49 | 0 | **355** | FAS | 8 | 31.71 | 0 | **4686** | NCBP1 | 4 | 17.66 | 0 |
| **3576** | IL8 | 2 | 6.43 | 0 | **8531** | CSDA | 10 | 28.01 | 0 | **1738** | DLD | 8 | 24.16 | 0 | **1892** | ECHS1 | 4 | 16.68 | 0 |
| **5328** | PLAU | 2 | 6 | 0 | **324** | APC | 10 | 23.87 | 0 | **3490** | IGFBP7 | 7 | 911.59 | 0 | **356** | FASLG | 4 | 16.34 | 0 |
| **1290** | COL5A2 | 2 | 4.93 | 0 | **6275** | S100A4 | 9 | 421.32 | 0 | **9260** | PDLIM7 | 7 | 286.24 | 0 | **10726** | NUDC | 4 | 15.07 | 0 |
| **5176** | SERPINF1 | 2 | 4.69 | 0 | **1291** | COL6A1 | 9 | 318.23 | 0 | **3659** | IRF1 | 7 | 272.21 | 0 | **6625** | SNRNP70 | 4 | 13.69 | 0 |
| **6997** | TDGF1 | 2 | 3.57 | 0 | **7040** | TGFB1 | 9 | 175.34 | 0 | **2006** | ELN | 7 | 230.96 | 0 | **6159** | RPL29 | 4 | 13.44 | 0 |

PABPC1 B2M HSPA9 TPI1

**IR60** 3 TERF1 PTBP1

CBL

| **6623** | SNCG | 2 | 3.4 | 0 | **6902** | TBCA | 9 | 147.55 | 0 | **5270** | SERPINE2 | 7 | 217.81 | 0 | **4125** | MAN2B1 | 4 | 13.31 | 0 |
| --- | --- | --- | --- | --- | --- | --- | --- | --- | --- | --- | --- | --- | --- | --- | --- | --- | --- | --- | --- |
| **4314** | MMP3 | 2 | 3.31 | 0 | **10092** | ARPC5 | 9 | 106.71 | 0 | **3927** | LASP1 | 7 | 212.97 | 0 | **6717** | SRI | 4 | 11.67 | 0 |
| **23193** | GANAB | 2 | 2.6 | 0 | **2** | A2M | 9 | 101.35 | 0 | **10092** | ARPC5 | 7 | 164.82 | 0 | **773** | CACNA1A | 4 | 11.51 | 0 |
| **3483** | IGFALS | 2 | 2.23 | 0 | **4089** | SMAD4 | 9 | 92.9 | 0 | **7917** | BAG6 | 7 | 163.85 | 0 | **5688** | PSMA7 | 4 | 11.23 | 0 |
| **56944** | OLFML3 | 2 | 2.14 | 0 | **5966** | REL | 9 | 82.29 | 0 | **1509** | CTSD | 7 | 136.17 | 0 | **1809** | DPYSL3 | 4 | 11.03 | 0 |
| **9802** | DAZAP2 | 2 | 1.85 | 0 | **5223** | PGAM1 | 9 | 55.53 | 0 | **375790** | AGRN | 7 | 122.6 | 0 | **842** | CASP9 | 4 | 10.94 | 0 |
| **2778** | GNAS | 2 | 1.83 | 0 | **10226** | PLIN3 | 9 | 52.02 | 0 | **6902** | TBCA | 7 | 112.97 | 0 | **1465** | CSRP1 | 4 | 9.68 | 0 |
| **4060** | LUM | 2 | 1.62 | 0 | **4690** | NCK1 | 9 | 39.11 | 0 | **6277** | S100A6 | 7 | 108.86 | 0 | **5788** | PTPRC | 4 | 9.62 | 0 |
| **1306** | COL15A1 | 2 | 1.58 | 0 | **10726** | NUDC | 9 | 38.3 | 0 | **8721** | EDF1 | 7 | 83.85 | 0 | **55573** | CDV3 | 4 | 8.68 | 0 |
| **7058** | THBS2 | 2 | 1.54 | 0 | **6606** | SMN1 | 9 | 29.84 | 0 | **4172** | MCM3 | 7 | 81.92 | 0 | **79001** | VKORC1 | 4 | 8.67 | 0 |
| **5118** | PCOLCE | 2 | 1.1 | 0 | **7248** | TSC1 | 9 | 19.95 | 0 | **7040** | TGFB1 | 7 | 73.88 | 0 | **1073** | CFL2 | 4 | 7.52 | 0 |
| **6422** | SFRP1 | 2 | 0.55 | 0 | **8985** | PLOD3 | 9 | 15.56 | 0 | **3175** | ONECUT1 | 7 | 68.25 | 0 | **1429** | CRYZ | 4 | 6.77 | 0 |
| **3908** | LAMA2 | 2 | 0 | 0 | **7076** | TIMP1 | 8 | 614.75 | 0 | **7124** | TNF | 7 | 59.01 | 0 | **230** | ALDOC | 4 | 2.73 | 0 |
| **57124** | CD248 | 1 | 0 | 0 | **9260** | PDLIM7 | 8 | 421.5 | 0 | **8417** | STX7 | 7 | 51.7 | 0 | **3897** | L1CAM | 3 | 393.75 | 0 |
| **54587** | MXRA8 | 1 | 0 | 0 | **6434** | TRA2B | 8 | 412.49 | 0 | **11100** | HNRNPUL | 7 | 47.39 | 0 | **2739** | GLO1 | 3 | 305.4 | 0 |
| **25878** | MXRA5 | 1 | 0 | 0 | **51631** | LUC7L2 | 8 | 287.85 | 0 | **8328** | GFI1B | 7 | 45.53 | 0 | **23549** | DNPEP | 3 | 269.6 | 0 |
| **6320** | CLEC11A | 1 | 0 | 0 | **3915** | LAMC1 | 8 | 279.12 | 0 | **6648** | SOD2 | 7 | 45.26 | 0 | **94121** | SYTL4 | 3 | 263.94 | 0 |
|  | | | | | **718** | C3 | 8 | 251 | 0 | **7114** | TMSB4X | 7 | 43.27 | 0 | **3481** | IGF2 | 3 | 217.09 | 0 |
|  |  |  |  |  | **3913** | LAMB2 | 8 | 249.03 | 0 | **1460** | CSNK2B | 7 | 41.12 | 0 | **1466** | CSRP2 | 3 | 207.28 | 0 |
|  |  |  |  |  | **716** | C1S | 8 | 221.2 | 0 | **2969** | GTF2I | 7 | 36.93 | 0 | **114990** | VASN | 3 | 91.02 | 0 |
|  |  |  |  |  | **5270** | SERPINE2 | 8 | 217.22 | 0 | **2806** | GOT2 | 7 | 30.33 | 0 | **4323** | MMP14 | 3 | 89.94 | 0 |
|  |  |  |  |  | **1508** | CTSB | 8 | 209.73 | 0 | **23410** | SIRT3 | 7 | 29.62 | 0 | **2286** | FKBP2 | 3 | 72.94 | 0 |
|  |  |  |  |  | **6277** | S100A6 | 8 | 197.08 | 0 | **10409** | BASP1 | 7 | 26.49 | 0 | **25870** | SUMF2 | 3 | 65.9 | 0 |
|  |  |  |  |  | **5499** | PPP1CA | 8 | 140.59 | 0 | **6625** | SNRNP70 | 7 | 23.53 | 0 | **5589** | PRKCSH | 3 | 62.49 | 0 |
|  |  |  |  |  | **2113** | ETS1 | 8 | 138.7 | 0 | **9124** | PDLIM1 | 7 | 22.59 | 0 | **4824** | NKX3-1 | 3 | 56.32 | 0 |
|  |  |  |  |  | **7422** | VEGFA | 8 | 119.78 | 0 | **831** | CAST | 7 | 19.26 | 0 | **9441** | MED26 | 3 | 51.94 | 0 |
|  |  |  |  |  | **1051** | CEBPB | 8 | 109.98 | 0 | **171024** | SYNPO2 | 7 | 17.78 | 0 | **6678** | SPARC | 3 | 49 | 0 |
|  |  |  |  |  | **1509** | CTSD | 8 | 105.53 | 0 | **1429** | CRYZ | 7 | 10.59 | 0 | **63827** | BCAN | 3 | 42.24 | 0 |
|  |  |  |  |  | **7124** | TNF | 8 | 104.32 | 0 | **23564** | DDAH2 | 6 | 332.24 | 0 | **7057** | THBS1 | 3 | 31.4 | 0 |
|  |  |  |  |  | **55752** | SEPT11 | 8 | 97.11 | 0 | **3009** | HIST1H1B | 6 | 148.78 | 0 | **80279** | CDK5RAP | 3 | 29.26 | 0 |
|  |  |  |  |  | **7917** | BAG6 | 8 | 92.16 | 0 | **25890** | ABI3BP | 6 | 126.12 | 0 | **10327** | AKR1A1 | 3 | 20.72 | 0 |
|  |  |  |  |  | **8417** | STX7 | 8 | 86.73 | 0 | **2113** | ETS1 | 6 | 114.28 | 0 | **10211** | FLOT1 | 3 | 18.17 | 0 |
|  |  |  |  |  | **6238** | RRBP1 | 8 | 76.14 | 0 | **10802** | SEC24A | 6 | 112.15 | 0 | **51606** | ATP6V1H | 3 | 17.19 | 0 |
|  |  |  |  |  | **3175** | ONECUT1 | 8 | 71.97 | 0 | **4314** | MMP3 | 6 | 75.89 | 0 | **157378** | TMEM65 | 3 | 16.67 | 0 |
|  |  |  |  |  | **2623** | GATA1 | 8 | 67.52 | 0 | **847** | CAT | 6 | 73.43 | 0 | **5004** | ORM1 | 3 | 16.3 | 0 |
|  |  |  |  |  | **1281** | COL3A1 | 8 | 66.26 | 0 | **4082** | MARCKS | 6 | 63.52 | 0 | **3107** | HLA-C | 3 | 16.12 | 0 |
|  |  |  |  |  | **338** | APOB | 8 | 66.04 | 0 | **6772** | STAT1 | 6 | 51.39 | 0 | **57510** | XPO5 | 3 | 13.99 | 0 |
|  |  |  |  |  | **9948** | WDR1 | 8 | 57.56 | 0 | **2908** | NR3C1 | 6 | 50.31 | 0 | **79869** | CPSF7 | 3 | 12.83 | 0 |
|  |  |  |  |  | **4172** | MCM3 | 8 | 56.68 | 0 | **22795** | NID2 | 6 | 48.02 | 0 | **596** | BCL2 | 3 | 12.52 | 0 |
|  |  |  |  |  | **2280** | FKBP1A | 8 | 43.5 | 0 | **6908** | TBP | 6 | 46.98 | 0 | **3685** | ITGAV | 3 | 11.33 | 0 |
|  |  |  |  |  | **2353** | FOS | 8 | 40.28 | 0 | **6314** | ATXN7 | 6 | 46.35 | 0 | **26472** | PPP1R14B | 3 | 9.77 | 0 |
|  |  |  |  |  | **4801** | NFYB | 8 | 25.15 | 0 | **11117** | EMILIN1 | 6 | 45.82 | 0 | **6623** | SNCG | 3 | 9.03 | 0 |
|  |  |  |  |  | **1738** | DLD | 8 | 18.61 | 0 | **558** | AXL | 6 | 45.45 | 0 | **998** | CDC42 | 3 | 8.94 | 0 |
|  |  |  |  |  | **3009** | HIST1H1B | 8 | 7.15 | 0 | **1051** | CEBPB | 6 | 44.77 | 0 | **10015** | PDCD6IP | 3 | 8.34 | 0 |
|  |  |  |  |  | **29766** | TMOD3 | 7 | 534.93 | 0 | **5340** | PLG | 6 | 43.4 | 0 | **112464** | PRKCDBP | 3 | 7.74 | 0 |
|  |  |  |  |  | **25932** | CLIC4 | 7 | 383.57 | 0 | **5430** | POLR2A | 6 | 31.07 | 0 | **6284** | S100A13 | 3 | 7.64 | 0 |
|  |  |  |  |  | **375790** | AGRN | 7 | 200.93 | 0 | **6449** | SGTA | 6 | 30.83 | 0 | **7040** | TGFB1 | 3 | 7.27 | 0 |
|  |  |  |  |  | **2006** | ELN | 7 | 154.82 | 0 | **2936** | GSR | 6 | 29.31 | 0 | **1632** | ECI1 | 3 | 6.96 | 0 |
|  |  |  |  |  | **10611** | PDLIM5 | 7 | 103.67 | 0 | **55573** | CDV3 | 6 | 28.37 | 0 | **9520** | NPEPPS | 3 | 6.9 | 0 |
|  |  |  |  |  | **3927** | LASP1 | 7 | 96.62 | 0 | **6670** | SP3 | 6 | 28.17 | 0 | **1808** | DPYSL2 | 3 | 6.68 | 0 |
|  |  |  |  |  | **2261** | FGFR3 | 7 | 94.18 | 0 | **3107** | HLA-C | 6 | 27.88 | 0 | **873** | CBR1 | 3 | 5.97 | 0 |
|  |  |  |  |  | **847** | CAT | 7 | 93.31 | 0 | **55729** | ATF7IP | 6 | 22.26 | 0 | **2040** | STOM | 3 | 5.75 | 0 |
|  |  |  |  |  | **4314** | MMP3 | 7 | 65.27 | 0 | **89953** | KLC4 | 6 | 22.13 | 0 | **83638** | C11orf68 | 3 | 5.17 | 0 |
|  |  |  |  |  | **4093** | SMAD9 | 7 | 64.93 | 0 | **6282** | S100A11 | 6 | 15.43 | 0 | **83442** | SH3BGRL | 3 | 4.87 | 0 |
|  |  |  |  |  | **55968** | NSFL1C | 7 | 53.87 | 0 | **1783** | DYNC1LI2 | 6 | 11.04 | 0 | **10961** | ERP29 | 3 | 4.07 | 0 |
|  |  |  |  |  | **29979** | UBQLN1 | 7 | 53.08 | 0 | **1265** | CNN2 | 6 | 7.69 | 0 | **3008** | HIST1H1E | 3 | 3.44 | 0 |
|  |  |  |  |  | **1822** | ATN1 | 7 | 51.04 | 0 | **712** | C1QA | 5 | 483.78 | 0 | **3101** | HK3 | 2 | 181.15 | 0 |
|  |  |  |  |  | **10493** | VAT1 | 7 | 49.33 | 0 | **284119** | PTRF | 5 | 478.03 | 0 | **8161** | COIL | 2 | 136.55 | 0 |
|  |  |  |  |  | **960** | CD44 | 7 | 48.07 | 0 | **6434** | TRA2B | 5 | 385.3 | 0 | **645** | BLVRB | 2 | 93.4 | 0 |
|  |  |  |  |  | **1786** | DNMT1 | 7 | 44.93 | 0 | **1471** | CST3 | 5 | 283.34 | 0 | **50808** | AK3 | 2 | 49.21 | 0 |
|  |  |  |  |  | **558** | AXL | 7 | 40.18 | 0 | **51631** | LUC7L2 | 5 | 265.94 | 0 | **5768** | QSOX1 | 2 | 47.23 | 0 |
|  |  |  |  |  | **30851** | TAX1BP3 | 7 | 34.04 | 0 | **4925** | NUCB2 | 5 | 138.56 | 0 | **22955** | SCMH1 | 2 | 32.76 | 0 |
|  |  |  |  |  | **823** | CAPN1 | 7 | 33.49 | 0 | **4926** | NUMA1 | 5 | 88.12 | 0 | **10533** | ATG7 | 2 | 27.99 | 0 |
|  |  |  |  |  | **10409** | BASP1 | 7 | 32.18 | 0 | **4176** | MCM7 | 5 | 85.64 | 0 | **1893** | ECM1 | 2 | 27.46 | 0 |
|  |  |  |  |  | **2936** | GSR | 7 | 27.47 | 0 | **203** | AK1 | 5 | 83.01 | 0 | **509** | ATP5C1 | 2 | 19.6 | 0 |
|  |  |  |  |  | **57175** | CORO1B | 7 | 26.5 | 0 | **3895** | KTN1 | 5 | 81.16 | 0 | **1978** | EIF4EBP1 | 2 | 17.61 | 0 |
|  |  |  |  |  | **2771** | GNAI2 | 7 | 21.82 | 0 | **4154** | MBNL1 | 5 | 68.78 | 0 | **7707** | ZNF148 | 2 | 13.66 | 0 |
|  |  |  |  |  | **6282** | S100A11 | 7 | 18.89 | 0 | **4171** | MCM2 | 5 | 62 | 0 | **2273** | FHL1 | 2 | 12.23 | 0 |
|  |  |  |  |  | **4234** | METTL1 | 7 | 15.96 | 0 | **2821** | GPI | 5 | 61.57 | 0 | **3698** | ITIH2 | 2 | 12.13 | 0 |
|  |  |  |  |  | **51474** | LIMA1 | 7 | 13.04 | 0 | **3094** | HINT1 | 5 | 54.7 | 0 | **5328** | PLAU | 2 | 10.83 | 0 |
|  |  |  |  |  | **1783** | DYNC1LI2 | 7 | 12.41 | 0 | **10449** | ACAA2 | 5 | 54.41 | 0 | **2805** | GOT1 | 2 | 10.15 | 0 |
|  |  |  |  |  | **171024** | SYNPO2 | 7 | 10.73 | 0 | **6696** | SPP1 | 5 | 44.62 | 0 | **2118** | ETV4 | 2 | 9.18 | 0 |
|  |  |  |  |  | **3490** | IGFBP7 | 6 | 830.49 | 0 | **2623** | GATA1 | 5 | 44.13 | 0 | **513** | ATP5D | 2 | 9.12 | 0 |
|  |  |  |  |  | **4082** | MARCKS | 6 | 174.24 | 0 | **5806** | PTX3 | 5 | 33.41 | 0 | **3487** | IGFBP4 | 2 | 8.77 | 0 |
|  |  |  |  |  | **633** | BGN | 6 | 167.96 | 0 | **6844** | VAMP2 | 5 | 33.33 | 0 | **5176** | SERPINF1 | 2 | 8.19 | 0 |
|  |  |  |  |  | **4925** | NUCB2 | 6 | 146.42 | 0 | **56902** | PNO1 | 5 | 31.95 | 0 | **2274** | FHL2 | 2 | 8.04 | 0 |
|  |  |  |  |  | **5589** | PRKCSH | 6 | 90.06 | 0 | **4125** | MAN2B1 | 5 | 29.66 | 0 | **10452** | TOMM40 | 2 | 7.83 | 0 |
|  |  |  |  |  | **22795** | NID2 | 6 | 84.51 | 0 | **1786** | DNMT1 | 5 | 28.35 | 0 | **4154** | MBNL1 | 2 | 7.66 | 0 |
|  |  |  |  |  | **10095** | ARPC1B | 6 | 57.59 | 0 | **6778** | STAT6 | 5 | 26.77 | 0 | **6386** | SDCBP | 2 | 7.26 | 0 |
|  |  |  |  |  | **112464** | PRKCDBP | 6 | 53.53 | 0 | **79001** | VKORC1 | 5 | 26.56 | 0 | **5902** | RANBP1 | 2 | 7.25 | 0 |
|  |  |  |  |  | **7458** | EIF4H | 6 | 47.46 | 0 | **1513** | CTSK | 5 | 19.79 | 0 | **6900** | CNTN2 | 2 | 6.44 | 0 |
|  |  |  |  |  | **1471** | CST3 | 6 | 44.51 | 0 | **204** | AK2 | 5 | 19.13 | 0 | **1856** | DVL2 | 2 | 5.48 | 0 |
|  |  |  |  |  | **5340** | PLG | 6 | 43.79 | 0 | **1991** | ELANE | 5 | 17.53 | 0 | **50** | ACO2 | 2 | 5.18 | 0 |
|  |  |  |  |  | **5430** | POLR2A | 6 | 40.32 | 0 | **1845** | DUSP3 | 5 | 16.63 | 0 | **8540** | AGPS | 2 | 4.89 | 0 |
|  |  |  |  |  | **5359** | PLSCR1 | 6 | 40.19 | 0 | **231** | AKR1B1 | 5 | 15.13 | 0 | **5864** | RAB3A | 2 | 4.58 | 0 |
|  |  |  |  |  | **998** | CDC42 | 6 | 39.95 | 0 | **10726** | NUDC | 5 | 14.09 | 0 | **54507** | ADAMTSL | 2 | 4.51 | 0 |
|  |  |  |  |  | **3094** | HINT1 | 6 | 38.76 | 0 | **3151** | HMGN2 | 5 | 12.53 | 0 | **2029** | ENSA | 2 | 4.49 | 0 |
|  |  |  |  |  | **5728** | PTEN | 6 | 36.51 | 0 | **4234** | METTL1 | 5 | 11.45 | 0 | **23643** | LY96 | 2 | 4.48 | 0 |
|  |  |  |  |  | **8328** | GFI1B | 6 | 31.8 | 0 | **1465** | CSRP1 | 5 | 10.81 | 0 | **1,01E+08** | RNF115 | 2 | 4.39 | 0 |
|  |  |  |  |  | **773** | CACNA1A | 6 | 31.65 | 0 | **338** | APOB | 5 | 10.23 | 0 | **9231** | DLG5 | 2 | 4.28 | 0 |
|  |  |  |  |  | **8841** | HDAC3 | 6 | 28.83 | 0 | **55968** | NSFL1C | 5 | 6.62 | 0 | **10951** | CBX1 | 2 | 4.26 | 0 |
|  |  |  |  |  | **2969** | GTF2I | 6 | 28.29 | 0 | **10493** | VAT1 | 5 | 5.14 | 0 | **5754** | PTK7 | 2 | 4.18 | 0 |
|  |  |  |  |  | **3008** | HIST1H1E | 6 | 27.9 | 0 | **230** | ALDOC | 5 | 3.52 | 0 | **3198** | HOXA1 | 2 | 4.14 | 0 |
|  |  |  |  |  | **1465** | CSRP1 | 6 | 25.77 | 0 | **11212** | PROSC | 5 | 3.23 | 0 | **29968** | PSAT1 | 2 | 3.43 | 0 |
|  |  |  |  |  | **55573** | CDV3 | 6 | 24.42 | 0 | **3481** | IGF2 | 4 | 361.01 | 0 | **178** | AGL | 2 | 3.4 | 0 |
|  |  |  |  |  | **204** | AK2 | 6 | 23.64 | 0 | **3660** | IRF2 | 4 | 261.12 | 0 | **26232** | FBXO2 | 2 | 2.99 | 0 |
|  |  |  |  |  | **1460** | CSNK2B | 6 | 23.1 | 0 | **509** | ATP5C1 | 4 | 251.44 | 0 | **10278** | EFS | 2 | 2.86 | 0 |
|  |  |  |  |  | **5954** | RCN1 | 6 | 22.91 | 0 | **4286** | MITF | 4 | 136.52 | 0 | **51700** | CYB5R2 | 2 | 2.63 | 0 |
|  |  |  |  |  | **1892** | ECHS1 | 6 | 18.6 | 0 | **5589** | PRKCSH | 4 | 120.33 | 0 | **4060** | LUM | 2 | 2.2 | 0 |
|  |  |  |  |  | **800** | CALD1 | 6 | 18.15 | 0 | **6498** | SKIL | 4 | 48.29 | 0 | **10209** | EIF1 | 2 | 1.52 | 0 |
|  |  |  |  |  | **355** | FAS | 6 | 16.23 | 0 | **4015** | LOX | 4 | 36.74 | 0 | **84681** | HINT2 | 2 | 1.52 | 0 |
|  |  |  |  |  | **6159** | RPL29 | 6 | 9.64 | 0 | **10539** | GLRX3 | 4 | 35.34 | 0 | **83657** | DYNLRB2 | 2 | 1.52 | 0 |
|  |  |  |  |  | **3481** | IGF2 | 5 | 411.83 | 0 | **4846** | NOS3 | 4 | 29.5 | 0 | **23593** | HEBP2 | 2 | 1.22 | 0 |
|  |  |  |  |  | **4286** | MITF | 5 | 186.5 | 0 | **5933** | RBL1 | 4 | 29.28 | 0 | **1212** | CLTB | 2 | 0.82 | 0 |
|  |  |  |  |  | **51726** | DNAJB11 | 5 | 119.09 | 0 | **23406** | COTL1 | 4 | 24.37 | 0 | **3074** | HEXB | 2 | 0 | 0 |
|  |  |  |  |  | **4130** | MAP1A | 5 | 112.94 | 0 | **25796** | PGLS | 4 | 24.16 | 0 | **3105** | HLA-A | 2 | 0 | 0 |
|  |  |  |  |  | **1464** | CSPG4 | 5 | 76.16 | 0 | **9111** | NMI | 4 | 21.92 | 0 | **221496** | LEMD2 | 2 | 0 | 0 |
|  |  |  |  |  | **7448** | VTN | 5 | 66.97 | 0 | **2029** | ENSA | 4 | 21.72 | 0 | **8341** | HIST1H2B | 2 | 0 | 0 |
|  |  |  |  |  | **284119** | PTRF | 5 | 57.13 | 0 | **26472** | PPP1R14B | 4 | 20.31 | 0 | **113146** | AHNAK2 | 1 | 0 | 0 |
|  |  |  |  |  | **10449** | ACAA2 | 5 | 56.13 | 0 | **3074** | HEXB | 4 | 19.86 | 0 | **1E+08** | DDTL | 1 | 0 | 0 |
|  |  |  |  |  | **4176** | MCM7 | 5 | 46.65 | 0 | **1809** | DPYSL3 | 4 | 17.96 | 0 | **90861** | HN1L | 1 | 0 | 0 |
|  |  |  |  |  | **10961** | ERP29 | 5 | 34.78 | 0 | **10961** | ERP29 | 4 | 17.75 | 0 | **51155** | HN1 | 1 | 0 | 0 |
|  |  |  |  |  | **9802** | DAZAP2 | 5 | 33.89 | 0 | **3685** | ITGAV | 4 | 16.61 | 0 | **1329** | COX5B | 1 | 0 | 0 |
|  |  |  |  |  | **7048** | TGFBR2 | 5 | 33.43 | 0 | **7422** | VEGFA | 4 | 16.39 | 0 | **30846** | EHD2 | 1 | 0 | 0 |
|  |  |  |  |  | **51060** | TXNDC12 | 5 | 32.21 | 0 | **8061** | FOSL1 | 4 | 15.83 | 0 | **1992** | SERPINB1 | 1 | 0 | 0 |
|  |  |  |  |  | **25890** | ABI3BP | 5 | 30.69 | 0 | **6876** | TAGLN | 4 | 15.74 | 0 | **1611** | DAP | 1 | 0 | 0 |
|  |  |  |  |  | **6696** | SPP1 | 5 | 29.71 | 0 | **6880** | TAF9 | 4 | 15.51 | 0 | **1340** | COX6B1 | 1 | 0 | 0 |
|  |  |  |  |  | **2670** | GFAP | 5 | 29.09 | 0 | **3096** | HIVEP1 | 4 | 13.92 | 0 | **23558** | WBP2 | 1 | 0 | 0 |
|  |  |  |  |  | **79001** | VKORC1 | 5 | 28.76 | 0 | **596** | BCL2 | 4 | 12.88 | 0 | **1520** | CTSS | 1 | 0 | 0 |
|  |  |  |  |  | **5806** | PTX3 | 5 | 28.46 | 0 | **3371** | TNC | 4 | 12.55 | 0 | **10763** | NES | 1 | 0 | 0 |
|  |  |  |  |  | **80279** | CDK5RAP | 5 | 27.99 | 0 | **55163** | PNPO | 4 | 12.05 | 0 | **10457** | GPNMB | 1 | 0 | 0 |
|  |  |  |  |  | **29968** | PSAT1 | 5 | 25.66 | 0 | **7099** | TLR4 | 4 | 11.85 | 0 | **51020** | HDDC2 | 1 | 0 | 0 |
|  |  |  |  |  | **10452** | TOMM40 | 5 | 23.6 | 0 | **1435** | CSF1 | 4 | 10.83 | 0 | **84836** | ABHD14B | 1 | 0 | 0 |
|  |  |  |  |  | **6844** | VAMP2 | 5 | 22.36 | 0 | **10015** | PDCD6IP | 4 | 10.29 | 0 | **50865** | HEBP1 | 1 | 0 | 0 |
|  |  |  |  |  | **89953** | KLC4 | 5 | 20.86 | 0 | **1808** | DPYSL2 | 4 | 10.23 | 0 | **54936** | ADPRHL2 | 1 | 0 | 0 |
|  |  |  |  |  | **11244** | ZHX1 | 5 | 20.28 | 0 | **9802** | DAZAP2 | 4 | 8.18 | 0 |  | | | | |
|  |  |  |  |  | **6314** | ATXN7 | 5 | 19.97 | 0 | **9520** | NPEPPS | 4 | 8.08 | 0 |  |  |  |  |  |
|  |  |  |  |  | **4125** | MAN2B1 | 5 | 18.18 | 0 | **9446** | GSTO1 | 4 | 7.88 | 0 |  |  |  |  |  |
|  |  |  |  |  | **1513** | CTSK | 5 | 18.16 | 0 | **842** | CASP9 | 4 | 6.71 | 0 |  |  |  |  |  |
|  |  |  |  |  | **1000** | CDH2 | 5 | 17.65 | 0 | **800** | CALD1 | 4 | 6.35 | 0 |  |  |  |  |  |
|  |  |  |  |  | **4686** | NCBP1 | 5 | 16.99 | 0 | **1073** | CFL2 | 4 | 6.05 | 0 |  |  |  |  |  |
|  |  |  |  |  | **596** | BCL2 | 5 | 15.89 | 0 | **873** | CBR1 | 4 | 5.41 | 0 |  |  |  |  |  |
|  |  |  |  |  | **231** | AKR1B1 | 5 | 14.93 | 0 | **23644** | EDC4 | 4 | 5.38 | 0 |  |  |  |  |  |
|  |  |  |  |  | **6625** | SNRNP70 | 5 | 14.76 | 0 | **112464** | PRKCDBP | 4 | 5.18 | 0 |  |  |  |  |  |
|  |  |  |  |  | **11100** | HNRNPUL | 5 | 13.95 | 0 | **1466** | CSRP2 | 3 | 198.5 | 0 |  |  |  |  |  |

| **1073** | CFL2 | 5 | 13.68 | 0 | **23549** | DNPEP | 3 | 142.04 | 0 |
| --- | --- | --- | --- | --- | --- | --- | --- | --- | --- |
| **8482** | SEMA7A | 5 | 10.68 | 0 | **5768** | QSOX1 | 3 | 135.5 | 0 |
| **9520** | NPEPPS | 5 | 9.81 | 0 | **4323** | MMP14 | 3 | 130.15 | 0 |
| **9094** | UNC119 | 5 | 9.28 | 0 | **10533** | ATG7 | 3 | 101.08 | 0 |
| **230** | ALDOC | 5 | 3.25 | 0 | **2118** | ETV4 | 3 | 64.94 | 0 |
| **1052** | CEBPD | 4 | 328.62 | 0 | **4772** | NFATC1 | 3 | 64.56 | 0 |
| **3491** | CYR61 | 4 | 132.43 | 0 | **2274** | FHL2 | 3 | 63.98 | 0 |
| **8614** | STC2 | 4 | 104.36 | 0 | **1329** | COX5B | 3 | 58.94 | 0 |
| **10533** | ATG7 | 4 | 84.3 | 0 | **2261** | FGFR3 | 3 | 53.02 | 0 |
| **7070** | THY1 | 4 | 67.03 | 0 | **6386** | SDCBP | 3 | 49.07 | 0 |
| **4772** | NFATC1 | 4 | 66.94 | 0 | **3339** | HSPG2 | 3 | 47.82 | 0 |
| **7707** | ZNF148 | 4 | 49.65 | 0 | **25870** | SUMF2 | 3 | 40.1 | 0 |
| **4824** | NKX3-1 | 4 | 49.06 | 0 | **4824** | NKX3-1 | 3 | 40.09 | 0 |
| **3516** | RBPJ | 4 | 43.51 | 0 | **6919** | TCEA2 | 3 | 38.75 | 0 |
| **2821** | GPI | 4 | 41.69 | 0 | **1893** | ECM1 | 3 | 35.02 | 0 |
| **10802** | SEC24A | 4 | 35.89 | 0 | **3897** | L1CAM | 3 | 33.53 | 0 |
| **4171** | MCM2 | 4 | 35.16 | 0 | **6456** | SH3GL2 | 3 | 30.22 | 0 |
| **4099** | MAG | 4 | 34.97 | 0 | **51726** | DNAJB11 | 3 | 28.26 | 0 |
| **4154** | MBNL1 | 4 | 34.34 | 0 | **896** | CCND3 | 3 | 26.26 | 0 |
| **4015** | LOX | 4 | 33.42 | 0 | **64236** | PDLIM2 | 3 | 25.26 | 0 |
| **8349** | HIST2H2B | 4 | 31.36 | 0 | **80279** | CDK5RAP | 3 | 24.23 | 0 |
| **56902** | PNO1 | 4 | 26.93 | 0 | **8291** | DYSF | 3 | 23.52 | 0 |
| **3074** | HEXB | 4 | 26.27 | 0 | **63827** | BCAN | 3 | 23.23 | 0 |
| **10015** | PDCD6IP | 4 | 25.52 | 0 | **29968** | PSAT1 | 3 | 22.4 | 0 |
| **6498** | SKIL | 4 | 23.98 | 0 | **7373** | COL14A1 | 3 | 18.03 | 0 |
| **3033** | HADH | 4 | 21.87 | 0 | **7322** | UBE2D2 | 3 | 16.94 | 0 |
| **8061** | FOSL1 | 4 | 19.53 | 0 | **5902** | RANBP1 | 3 | 16.45 | 0 |
| **6908** | TBP | 4 | 18.79 | 0 | **5004** | ORM1 | 3 | 14.56 | 0 |
| **1991** | ELANE | 4 | 16.51 | 0 | **10540** | DCTN2 | 3 | 13.74 | 0 |
| **1009** | CDH11 | 4 | 15.3 | 0 | **10452** | TOMM40 | 3 | 11.57 | 0 |
| **6876** | TAGLN | 4 | 14.08 | 0 | **3198** | HOXA1 | 3 | 11.52 | 0 |
| **1809** | DPYSL3 | 4 | 12.86 | 0 | **327** | APEH | 3 | 10.41 | 0 |
| **5788** | PTPRC | 4 | 11.32 | 0 | **84528** | RHOXF2 | 3 | 8.87 | 0 |
| **842** | CASP9 | 4 | 8.75 | 0 | **1293** | COL6A3 | 3 | 8.74 | 0 |
| **197131** | UBR1 | 4 | 8.2 | 0 | **3698** | ITIH2 | 3 | 7.46 | 0 |
| **5764** | PTN | 4 | 8.06 | 0 | **998** | CDC42 | 3 | 7.36 | 0 |
| **1808** | DPYSL2 | 4 | 3.95 | 0 | **8614** | STC2 | 3 | 6.96 | 0 |
| **2817** | GPC1 | 3 | 246.46 | 0 | **54507** | ADAMTSL | 3 | 6.68 | 0 |
| **1466** | CSRP2 | 3 | 187.21 | 0 | **5788** | PTPRC | 3 | 6.27 | 0 |
| **3075** | CFH | 3 | 159.26 | 0 | **6284** | S100A13 | 3 | 6.08 | 0 |
| **1303** | COL12A1 | 3 | 138.49 | 0 | **2771** | GNAI2 | 3 | 5.79 | 0 |
| **1289** | COL5A1 | 3 | 125.51 | 0 | **1292** | COL6A2 | 3 | 5.02 | 0 |
| **509** | ATP5C1 | 3 | 115.72 | 0 | **6623** | SNCG | 3 | 4.74 | 0 |
| **4323** | MMP14 | 3 | 107.11 | 0 | **2778** | GNAS | 3 | 3.59 | 0 |
| **25870** | SUMF2 | 3 | 77.68 | 0 | **1854** | DUT | 3 | 1.67 | 0 |
| **1266** | CNN3 | 3 | 66.9 | 0 | **3105** | HLA-A | 3 | 0.99 | 0 |
| **4982** | TNFRSF11 | 3 | 62.43 | 0 | **3101** | HK3 | 2 | 166.17 | 0 |
| **1,01E+08** | RNF115 | 3 | 60.68 | 0 | **645** | BLVRB | 2 | 94.74 | 0 |
| **712** | C1QA | 3 | 50.32 | 0 | **10653** | SPINT2 | 2 | 72.28 | 0 |
| **2274** | FHL2 | 3 | 40.68 | 0 | **1266** | CNN3 | 2 | 57 | 0 |
| **2118** | ETV4 | 3 | 35.07 | 0 | **50** | ACO2 | 2 | 56.07 | 0 |
| **25796** | PGLS | 3 | 28.8 | 0 | **6403** | SELP | 2 | 46.45 | 0 |
| **56931** | DUS3L | 3 | 27.15 | 0 | **56931** | DUS3L | 2 | 46.07 | 0 |
| **7373** | COL14A1 | 3 | 24.06 | 0 | **2247** | FGF2 | 2 | 41.05 | 0 |
| **1293** | COL6A3 | 3 | 20.97 | 0 | **4153** | MBL2 | 2 | 37.63 | 0 |
| **3727** | JUND | 3 | 18.49 | 0 | **3075** | CFH | 2 | 24.71 | 0 |
| **710** | SERPING1 | 3 | 17.78 | 0 | **114990** | VASN | 2 | 21.25 | 0 |
| **5004** | ORM1 | 3 | 17.3 | 0 | **6688** | SPI1 | 2 | 16.97 | 0 |
| **5328** | PLAU | 3 | 16.94 | 0 | **50808** | AK3 | 2 | 16.09 | 0 |
| **114990** | VASN | 3 | 14 | 0 | **66036** | MTMR9 | 2 | 13.45 | 0 |
| **5155** | PDGFB | 3 | 13.47 | 0 | **3024** | HIST1H1A | 2 | 13.38 | 0 |
| **2243** | FGA | 3 | 12.73 | 0 | **23558** | WBP2 | 2 | 13.2 | 0 |
| **5933** | RBL1 | 3 | 12.45 | 0 | **710** | SERPING1 | 2 | 12.78 | 0 |
| **63827** | BCAN | 3 | 11.68 | 0 | **84572** | GNPTG | 2 | 10.83 | 0 |
| **720** | C4A | 3 | 11.11 | 0 | **2273** | FHL1 | 2 | 10.75 | 0 |
| **3698** | ITIH2 | 3 | 9.7 | 0 | **3487** | IGFBP4 | 2 | 9.62 | 0 |
| **6880** | TAF9 | 3 | 8.9 | 0 | **6672** | SP100 | 2 | 9.34 | 0 |
| **4060** | LUM | 3 | 8.34 | 0 | **10457** | GPNMB | 2 | 9.09 | 0 |
| **6778** | STAT6 | 3 | 8.21 | 0 | **4192** | MDK | 2 | 8.42 | 0 |
| **3918** | LAMC2 | 3 | 8.17 | 0 | **5328** | PLAU | 2 | 8.12 | 0 |
| **3685** | ITGAV | 3 | 7.45 | 0 | **7707** | ZNF148 | 2 | 8.07 | 0 |
| **1234** | CCR5 | 3 | 7.09 | 0 | **7704** | ZBTB16 | 2 | 7.34 | 0 |
| **8291** | DYSF | 3 | 6.9 | 0 | **1978** | EIF4EBP1 | 2 | 7.12 | 0 |
| **6284** | S100A13 | 3 | 5.91 | 0 | **720** | C4A | 2 | 7.03 | 0 |
| **327** | APEH | 3 | 5.9 | 0 | **81889** | FAHD1 | 2 | 6.79 | 0 |
| **10951** | CBX1 | 3 | 5.64 | 0 | **1E+08** | DDTL | 2 | 6.15 | 0 |
| **22943** | DKK 1,00 | 3 | 5.3 | 0 | **2805** | GOT1 | 2 | 5.53 | 0 |
| **23644** | EDC4 | 3 | 5.22 | 0 | **2739** | GLO1 | 2 | 5.49 | 0 |
| **1292** | COL6A2 | 3 | 4.31 | 0 | **8540** | AGPS | 2 | 4.94 | 0 |
| **6623** | SNCG | 3 | 4.19 | 0 | **1295** | COL8A1 | 2 | 4.88 | 0 |
| **2778** | GNAS | 3 | 3.51 | 0 | **6352** | CCL5 | 2 | 4.48 | 0 |
| **1212** | CLTB | 3 | 1.57 | 0 | **55367** | PIDD | 2 | 4.34 | 0 |
| **440689** | HIST2H2B | 3 | 0.67 | 0 | **3603** | IL16 | 2 | 4.27 | 0 |
| **5089** | PBX2 | 2 | 404.75 | 0 | **6695** | SPOCK1 | 2 | 3.62 | 0 |
| **5156** | PDGFRA | 2 | 213.93 | 0 | **7058** | THBS2 | 2 | 3.57 | 0 |
| **3101** | HK3 | 2 | 184.19 | 0 | **9518** | GDF15 | 2 | 3.27 | 0 |
| **6403** | SELP | 2 | 137.56 | 0 | **5754** | PTK7 | 2 | 3.27 | 0 |
| **4153** | MBL2 | 2 | 107.22 | 0 | **27332** | ZNF638 | 2 | 3.05 | 0 |
| **2247** | FGF2 | 2 | 99.02 | 0 | **10951** | CBX1 | 2 | 2.7 | 0 |
| **645** | BLVRB | 2 | 93.22 | 0 | **3483** | IGFALS | 2 | 2.07 | 0 |
| **10653** | SPINT2 | 2 | 82.22 | 0 | **3008** | HIST1H1E | 2 | 2.01 | 0 |
| **860** | RUNX2 | 2 | 32.97 | 0 | **4060** | LUM | 2 | 1.58 | 0 |
| **2776** | GNAQ | 2 | 30.9 | 0 | **5118** | PCOLCE | 2 | 0.79 | 0 |
| **1893** | ECM1 | 2 | 24.81 | 0 | **1520** | CTSS | 2 | 0.58 | 0 |
| **5768** | QSOX1 | 2 | 19.81 | 0 | **6422** | SFRP1 | 2 | 0.26 | 0 |
| **10457** | GPNMB | 2 | 14.09 | 0 | **1212** | CLTB | 2 | 0 | 0 |
| **3487** | IGFBP4 | 2 | 11.72 | 0 | **2745** | GLRX | 2 | 0 | 0 |
| **6688** | SPI1 | 2 | 11.25 | 0 | **84681** | HINT2 | 2 | 0 | 0 |
| **3489** | IGFBP6 | 2 | 10.74 | 0 | **9550** | ATP6V1G1 | 2 | 0 | 0 |
| **3024** | HIST1H1A | 2 | 9.59 | 0 | **30846** | EHD2 | 2 | 0 | 0 |
| **4192** | MDK | 2 | 9.4 | 0 | **22883** | CLSTN1 | 2 | 0 | 0 |
| **8600** | TNFSF11 | 2 | 8.43 | 0 | **440689** | HIST2H2B | 2 | 0 | 0 |
| **3198** | HOXA1 | 2 | 6.19 | 0 | **51155** | HN1 | 1 | 0 | 0 |
| **8540** | AGPS | 2 | 5.86 | 0 | **6781** | STC1 | 1 | 0 | 0 |
| **5754** | PTK7 | 2 | 5.56 | 0 | **1611** | DAP | 1 | 0 | 0 |
| **80328** | ULBP2 | 2 | 5.52 | 0 | **7873** | MANF | 1 | 0 | 0 |
| **2739** | GLO1 | 2 | 5.02 | 0 | **389898** | UBE2NL | 1 | 0 | 0 |
| **6352** | CCL5 | 2 | 4.99 | 0 | **10763** | NES | 1 | 0 | 0 |
| **7058** | THBS2 | 2 | 4.18 | 0 | **51020** | HDDC2 | 1 | 0 | 0 |
| **7704** | ZBTB16 | 2 | 4.14 | 0 | **84836** | ABHD14B | 1 | 0 | 0 |
| **6997** | TDGF1 | 2 | 3.98 | 0 | **54587** | MXRA8 | 1 | 0 | 0 |
| **57510** | XPO5 | 2 | 3.39 | 0 | **6320** | CLEC11A | 1 | 0 | 0 |
| **140901** | STK35 | 2 | 3.28 | 0 |  | | | | |
| **25878** | MXRA5 | 2 | 3.13 | 0 |  |  |  |  |  |
| **6442** | SGCA | 2 | 3.12 | 0 |  |  |  |  |  |
| **3483** | IGFALS | 2 | 2.29 | 0 |  |  |  |  |  |
| **23643** | LY96 | 2 | 2.19 | 0 |  |  |  |  |  |
| **1290** | COL5A2 | 2 | 1.26 | 0 |  |  |  |  |  |
| **5118** | PCOLCE | 2 | 0.75 | 0 |  |  |  |  |  |
| **6422** | SFRP1 | 2 | 0.21 | 0 |  |  |  |  |  |
| **51155** | HN1 | 2 | 0 | 0 |  |  |  |  |  |
| **6695** | SPOCK1 | 2 | 0 | 0 |  |  |  |  |  |
| **83657** | DYNLRB2 | 2 | 0 | 0 |  |  |  |  |  |
| **30846** | EHD2 | 2 | 0 | 0 |  |  |  |  |  |
| **22883** | CLSTN1 | 2 | 0 | 0 |  |  |  |  |  |
| **6781** | STC1 | 1 | 0 | 0 |  |  |  |  |  |
| **1611** | DAP | 1 | 0 | 0 |  |  |  |  |  |
| **57124** | CD248 | 1 | 0 | 0 |  |  |  |  |  |
| **7873** | MANF | 1 | 0 | 0 |  |  |  |  |  |
| **84572** | GNPTG | 1 | 0 | 0 |  |  |  |  |  |
| **10763** | NES | 1 | 0 | 0 |  |  |  |  |  |
| **54587** | MXRA8 | 1 | 0 | 0 |  |  |  |  |  |
| **6320** | CLEC11A | 1 | 0 | 0 |  |  |  |  |  |
