# Supplementary figures and images for: Progression of irradiated mesenchymal stromal cells from early to late senescence: Changes in SASP composition and anti‐tumour properties
Source: Cell Prolif. 2023 Mar 22;56(6):e13401. doi: 10.1111/cpr.13401 (PMC10280137; doi:10.1111/cpr.13401)

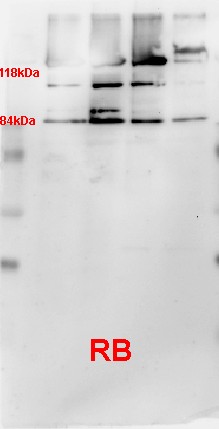

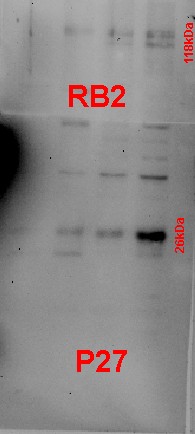


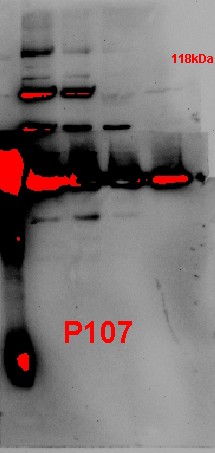

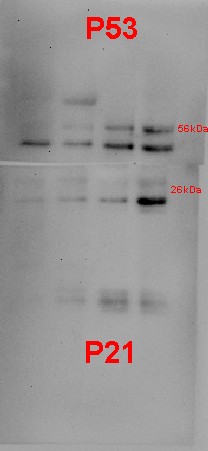


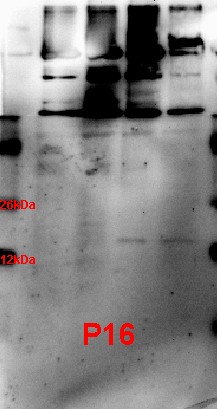

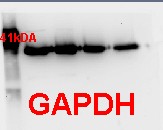

Supplement: Supplementary file 5 — Supplementary file S5. Western blot raw data. Uncropped western blot bands reported in Figure 2B. [file CPR-56-e13401-s002.docx]
